# Comprehensive Metabolic Profiling of Euphorbiasteroid in Rats by Integrating UPLC-Q/TOF-MS and NMR as Well as Microbial Biotransformation

**DOI:** 10.3390/metabo12090830

**Published:** 2022-09-02

**Authors:** Sijia Xiao, Xike Xu, Xintong Wei, Jiayun Xin, Shanshan Li, Yanhui Lv, Wei Chen, Wenlin Yuan, Bin Xie, Xianpeng Zu, Yunheng Shen

**Affiliations:** 1 School of Pharmacy, Naval Medical University, Shanghai 200433, China; 2 School of Pharmacy, Shandong University of Traditional Chinese Medicine, Jinan 250355, China; 3 School of Pharmaceutical Sciences and Yunnan Key Laboratory of Pharmacology for Natural Products, Kunming Medical University, Kunming 650500, China

**Keywords:** euphorbiasteroid, metabolic pathway in vivo, UPLC-Q/TOF-MS, *Cunninghamella elegans*, microbial biotransformation

## Abstract

Euphorbiasteroid, a lathyrane-type diterpene from *Euphorbiae semen* (the seeds of *Euphorbia lathyris* L.), has been shown to have a variety of pharmacological effects such as anti-tumor and anti-obesity. This study aims to investigate the metabolic profiles of euphorbiasteroid in rats and rat liver microsomes (RLMs) and *Cunninghamella elegans* bio-110930 by integrating ultra-performance liquid chromatography-quadrupole time-of-flight-mass spectrometry (UPLC-Q/TOF-MS), UNIFI software, and NMR techniques. A total of 31 metabolites were identified in rats. Twelve metabolites (**M1**–**M5**, **M8**, **M12**–**M13**, **M16**, **M24**–**M25**, and **M29**) were matched to the metabolites obtained by RLMs incubation and the microbial transformation of *C. elegans* bio-110930 and their structures were exactly determined through analysis of NMR spectroscopic data. In addition, the metabolic pathways of euphorbiasteroid were then clarified, mainly including hydroxylation, hydrolysis, oxygenation, sulfonation, and glycosylation. Finally, three metabolites, **M3** (20-hydroxyl euphorbiasteroid), **M24** (epoxylathyrol) and **M25** (15-deacetyl euphorbiasteroid), showed significant cytotoxicity against four human cell lines with IC_50_ values from 3.60 μM to 40.74 μM. This is the first systematic investigation into the in vivo metabolic pathways of euphorbiasteroid and the cytotoxicity of its metabolites, which will be beneficial for better predicting the metabolism profile of euphorbiasteroid in humans and understanding its possible toxic material basis.

## 1. Introduction

**Figure 1:**
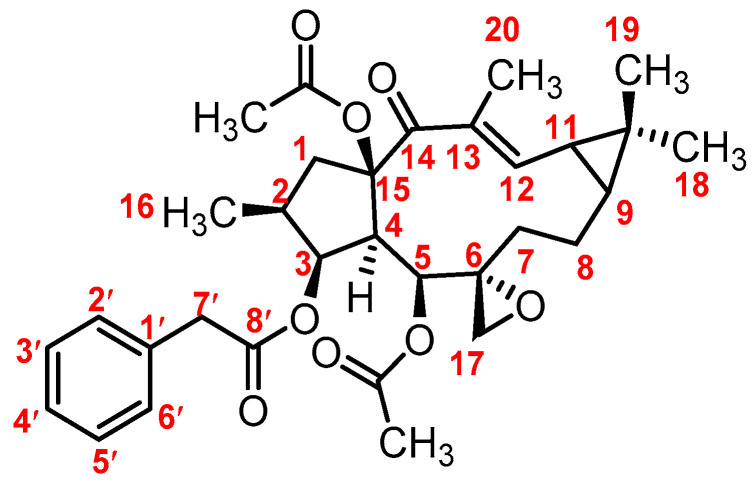
The chemical structure of euphorbiasteroid (**M0**).

*Euphorbiae semen*, known as “Qian-Jin-Zi” in China, is the dried and ripe seeds of *Euphorbia lathyris* L. According to the 2020 edition of the Chinese Pharmacopoeia, *Euphorbiae semen* could be clinically used either alone or in combination with other herbal medicines as remedies for reducing water and phlegm retention, promoting blood circulation, removing blood stasis, curing tinea and scabies, and treating amenorrhea, snakebites, terminal schistosomiasis, anuria, and constipation. It is worth noting that *Euphorbiae semen* was listed as one of the 28 toxic Chinese herbal medicines in the Chinese Pharmacopoeia [1]. It has been proven to exhibit a strong stimulating effect on the gastrointestinal tract, and its intensity is three times that of croton oil. The main toxic components of Euphorbiae semen are diterpenoids, identified by means of fractionation. Euphorbiasteroid (**M0**, Figure 1), a natural lathyrane-type diterpenoid, was deemed as one of the intestinal tract stimulant constituents to induce diarrhea [2]. Moreover, euphorbiasteroid has the potential to reverse resistance to anticancer drugs in MES-SA/Dx5 cells [3], and also exhibits anti-obesity [4], and anti-tumor activities [5], meaning that it has broad biomedical research prospects. Previous studies have shown that euphorbiasteroid is one of the main toxic and active components of *Euphorbiae semen*, and has also been used as a quantitative index for quality control in Chinese pharmacopoeia [6]. However, there is still a lack of research on the metabolism of diterpenoids in euphorbiasteroid, which brings difficulties to the clinically safe use of this toxic Chinese medicine. Thus, in order to further understand the toxic and active mechanisms of euphorbiasteroid, a systematic study of its metabolic profiles is of great significance.

The in-depth study of drug metabolism can clarify the metabolic pathways as well as the toxic and active mechanisms of drugs so as to ensure their safety and lay a theoretical foundation for clinical application and toxic and side effect detection [7]. Therefore, it is of great significance and value to establish a sensitive and reliable analytical method for the identification of drug metabolites. Liquid chromatography (LC) is one of the main methods used to analyze active pharmaceutical ingredients (APIs) in American and European Pharmacopoeia, and has been combined with a variety of detectors, such as fluorescence detectors, electrochemical detectors or flame photometric detectors. However, these detectors cannot clearly identify compounds separated by LC. In contrast, high performance liquid chromatography-mass spectrometry (HPLC-MS), with liquid chromatography as the separation system and mass spectrometry as the detection system, combines the high separation capability of HPLC for complex samples with the high selectivity and sensitivity of mass spectrometry and its ability to provide relative molecular mass and structural information, perfectly compensating for this deficiency [8,9]. Especially, ultra-performance liquid chromatography-quadrupole time-of-flight-mass spectrometry (UPLC-Q/TOF-MS) combined with the computer-aided identification platform Waters UNIFI, has become a powerful analytical tool, which has the advantages of accurate mass measurement, efficient separation technology, and rapid identification of metabolites, and has been widely applied in the metabolism research of traditional Chinese medicine in recent years [10]. However, the main disadvantage of mass spectrometry analysis is that the molecular weight of structural characterization depends on the collision-induced dissociation of protonated molecular ions of target compounds, so it cannot provide accurate metabolite structure in most cases [11]. Nuclear magnetic resonance (NMR) is a complementary analytical method, which can characterize metabolites in more detail. The advantages of the combination of UPLC-Q/TOF-MS and NMR have been verified in several reports in recent years [12,13,14]. However, the sensitivity of NMR is not as good as that of mass spectrometry, and the presence of impurities in the sample has great influence on NMR signal. Therefore, it is necessary to separate the analyte from the impurity well and obtain enough quantity for structural identification by NMR. Generally, supplementary methods can be used to obtain a sufficient sample amount of metabolites, including chemical methods [11] and small experimental animal models [15], microsomal preparations [16,17], enzyme-catalyzed reactions [18,19], microbial transformation [11,20] and so on. Compared with other methods, microbial transformation is more convenient and economical, especially with the advantages of in vitro large-scale preparation [20,21,22,23,24]. In particular, the filamentous zygote fungus *C. elegans* has been shown to possess a human-like cytochrome P450 monooxygenase system, including the CYP509A1 isoenzyme that is similar to the CYP51 family, thus producing similar metabolic profiles to mammalian animals [25,26].Sufficient samples for structural characterization based on NMR techniques as well as the evaluation of bioactivity and toxicity can thus be acquired. Therefore, a combination of in vivo animal experiments and in vitro microbial transformation will contribute to accurately elucidating the structures of metabolites.

The current work aimed to identify the metabolites of euphorbiasteroid in rats and in vitro models (RLMs and *C. elegans* bio-110930), by using UPLC-Q/TOF-MS and the UNIFI platform as well as NMR technique, and to rationalize the elimination mechanism of euphorbiasteroid, in which the biotransformation based on the fungus *C. elegans* bio-110930 and chemical hydrolysis were applied to prepare the samples of potential metabolites. This method has been used in the previous research of our research group and is reasonable [27]. Finally, a total of 31 metabolites in vivo, including four phase II metabolites, were detected and identified. Additionally, the structures of 12 metabolites were accurately characterized and confirmed by structural elucidation based on NMR technique and by comparing the chromatography retention times and mass spectra with those of standard compounds from the biotransformation products of *C. elegans* bio-110930. Then, the metabolic pathway of euphorbiasteroid in rats was rationally elucidated on the basis of the study of metabolism in vivo and in vitro. The cytotoxic assay showed that three metabolites of euphorbiasteroid (**M3**, **M24**-**M25**) have cytotoxicity on four strains of human cells (SH-SY5Y, LO2, AC-16, and HK-2) with the IC_50_ values from 3.60 μM to 40.74 μM, while euphorbiasteroid did not show cytotoxicity on the same cell lines (IC_50_ > 50 μM). Therefore, our research findings will provide new insights into the metabolism mechanism of euphorbiasteroid and the possible toxicity of the metabolites, be beneficial for understanding the in vivo elimination process of euphorbiasteroid, and provide data support and reference for safe, reasonable and controllable clinical application of Euphorbiae semen.

## 2. Materials and Methods

### 2.1. Chemicals and Reagents

Euphorbiasteroid was purchased from Chengdu Herbpurify Co., Ltd. (Chengdu, China). Soybean oil was bought from Ron Pharm (Shanghai) Co., Limited. Sabouraud dextrose broth was procured from Qingdao Hope Bio-Technology Co., Ltd. (cat no. HB0233, Qingdao, China). Sabouraud dextrose agar was obtained from Solarbio (cat no. P9240, Beijing, China). The fungal strain, *Cunninghamella elegans* (bio-110930), was purchased from the Beijing baioubowei Biotechnology Co., Ltd. (Beijing, China). Pooled rat liver microsomes (RLM), Gentest ^TM^ NADPH regenerating system solution A (26.1 mM β-nicotinamide adenine dinucleotide phosphate (NADP^+^), 66 mM D-glucose-6-phosphate (Glc-6-P), 66 mM magnesium chloride (MgCl_2_) in water and solution B (40 U/mL Glc-6-P dehydrogenase (Glc-6-P-DH) in sodium citrate (0.05 mM)), and 0.1 M PBS buffer were purchased from IPHASE Biosciences (Beijing, China). Column chromatography (CC) was performed using Sephadex LH-20 gel (GE Medical Systems Ltd., Buckinghamshire, UK). Ethyl acetate and acetone were analytical grade from Shenyang Chemical Reagent Co., Ltd. (Shenyang, China). LC-MS-grade acetonitrile, methanol and formic acid were purchased from Fisher-Scientific (Fair Lawn, NJ, USA) and were used in the mobile phase and sample preparation. LC-MS-grade leucine enkephalin was obtained from Sigma-Aldrich (St. Louis, MO, USA). Ultra-pure water was purified by a Milli-Q system (Millipore, Bedford, MA, USA). All other reagents were of analytical reagent grade. SH-SY5Y neuroblastoma cells, LO2 cells, AC-16 cells, and HK-2 cells were purchased from Shanghai Institute of Biochemistry and Cell Biology, Chinese Academy of Sciences (Shanghai, China). CCK-8 assay kit was obtained from Beyotime Biotechnology (Shanghai, China). 6 cm/10 cm Petri dishes and 96-well plates were obtained from Corning Incorporated (Corning, NY, USA).

### 2.2. Instrumentation and Analysis Conditions

For metabolite separation and detection, chromatographic analyses were performed using a Waters Acquity UPLC I-class system (Waters, Milford, MA, USA), equipped with an auto-sampler, a binary solvent delivery system, an online degasser, and a photodiode array detector. An ACQUITY UPLC^®^ HSS T3 column (2.1 × 150 mm, 1.8 μm, Waters) protected with a HSS T3 VanGuard ™ Pre-Column 3/Pk (2.1 × 5.0 mm, 1.8 μm, Waters) was used. The optimized parameters were set as follows: the mobile phase consisted of eluent A (0.1% formic acid in water, *v*/*v*) and eluent B (acetonitrile). The flow rate was 0.3 mL/min. The column and auto-sampler temperatures were maintained at 40 °C and 4 °C, respectively. The gradient elution program was optimized as follows: 0–9 min, 30–70% B; 9–13 min, 70–90% B; 13–17 min, 90–100% B; 17–20 min, 100–100% B.

The mass spectrometry detection was performed on the SYNAPT G2-Si HDMS system, equipped with an electrospray ionization (ESI) source (Waters Corp., Manchester, UK). A positive ion mode was conducted in this analysis. Mass spectrometry conditions were finally set as follows: capillary voltage of 3.0 kV, cone voltage of 40 V, source temperature of 120 °C, and de-solvation temperature of 400 °C. Nitrogen was used as the desolvation and cone gas with a flow rate of 800 and 50 L/h, respectively, and the full-scan mass range was set as *m*/*z* 50–1500 Da. In the auto mass spectrometry mode, the collision-induced dissociation energies were set at 0 eV for the precursor ion at the low-energy mode, and the collision-induced dissociation energies were set from 2 to 10 eV for the high-energy mode. Real-time data were calibrated using an external reference (LockSpray™) at a concentration of 0.2 ng/mL with an infusion flow rate of 5 μL/min, generating a reference ion for the positive ion mode (*m*/*z* 556.2771) during the UPLC-MS analysis. Data were acquired and processed using MassLynx ™ NT 4.1 software (Waters, Milford, MA, USA).

Accurate molecular weights of some metabolites were acquired using an Agilent 6520 Accurate Mass quadrupole time-of-flight mass spectrometer (Q-TOF MS; Agilent Technologies, Santa Clara, CA, USA). The capillary voltage of the ion source was set at 3.0 kV in positive ion mode. Nitrogen was used as the de-solvation and nebulizing gas at a constant temperature of 350 °C. The scan range was set at *m*/*z* 100–1500 Da.

The isolation and purification of metabolites were achieved using an Agilent 1200 series Semi-preparative High Performance Liquid Chromatography (HPLC) system (Palo Alto, CA, USA) consisting of a G1311A quat pump solvent delivery system, a G1379A degasser unit, a G1313A autosampler, and a G1315B DAD detector. The preparation was performed with a Zorbax SB-C18 (5 μm, 9.4 mm × 25 cm) column (Agilent Technologies, Santa Clara, CA, USA). The wavelength was set at 280 nm.

Nuclear magnetic resonance (NMR) spectra of euphorbiasteroid and metabolites were measured on Bruker AV-500 spectrometers (Faellanden, Switzerland) using tetramethylsilane as an internal standard.

### 2.3. Animal and Drug Administration

Male Sprague-Dawley rats (200–220 g) were commercially supplied by Shanghai Sippr-BK Laboratory Animal Co., Ltd. (Shanghai, China) and were housed in a humidity- and temperature-controlled room (50 ± 10% and 22–24 °C) with a 12-h light/dark cycle. The experimental rats were allowed to access food and water ad libitum and acclimatized to the conditions mentioned above for a week, then fasted overnight but with free access to water before the experiments. Euphorbiasteroid was dissolved in soybean oil solution (containing 0.5% ethanol) to form a concentration of 10 mg/mL. A single dose of 100 mg/kg euphorbiasteroid was orally administered to rats and the same concentration of soybean oil solution (containing 0.5% ethanol) was administered as a blank control. All animal procedures were performed in accordance with the Guidelines for Care and Use of Laboratory Animals of Naval Medical University and approved by the Animal Ethics Committee of Naval Medical University.

### 2.4. Sample Collection of Blood, Urine, and Feces

Blood samples (0.5 mL) were collected from six rats through the orbital sinus before administration (blank sample) and 0.25, 0.5, 1, 2, 4, 6, and 12 h after administration. Plasma samples were prepared by centrifugation at 4000 rpm for 10 min. For urine and feces sampling, 12 rats were divided into an administration group and a blank group, and were placed separately in stainless steel metabolic cages. Urine and feces samples were collected in containers surrounded by ice over 0–6, 6–12, and 12–24 h after drug administration. The mixed urine samples were centrifuged at 4000 rpm for 10 min at 4 °C to obtain the supernatants, and fecal samples were left in a cool and dry place until dry. All the biological samples were frozen at −80 °C before analysis.

### 2.5. Preparation of Blood, Urine, and Feces Samples

An aliquot of 200 µL of plasma and urine samples was put in a 1.5 mL tube, respectively. 800 µL of acetonitrile was added and vortexed for 5 min to extract metabolites. Feces samples (1.0 g) were crushed and then ultrasonically extracted with acetonitrile (10 mL) for 30 min. All the above-mentioned mixtures were centrifuged at 13,000 rpm at 4 °C for 10 min. The supernatants were then transferred and evaporated to dryness under a nitrogen stream at 30 °C. The residues were dissolved in 100 µL of methanol and then centrifuged at 13,000 rpm at 4 °C for 10 min. All supernatants were injected into the UPLC-Q/TOF-MS system for analysis.

### 2.6. In Vitro Incubation of Euphorbiasteroid with Rat Liver Microsomes

The microsomal incubation approach was based on previous metabolism studies published by Wintermeyer et al. [28] and Franziska et al. [17]. A 200 μL incubation system containing 10 μL of solution A, 2 μL of solution B, 5 μL of rat liver microsomes (20 mg/mL) and 182 μL 0.1 M PBS buffer (pH = 7.4) was constructed. The above solution was heated in a 37 °C water bath, then 1 μL of euphorbiasteroid (dissolved in DMSO solution, 10 mM) was used to start the reaction, and the mixture was then incubated at 37 °C for 1 h. The reactions were terminated by the addition of 200 μL of ice-cold acetonitrile. The mixture was then centrifuged at 13,000 rpm for 10 min, and a 2-μL aliquot of the supernatant was directly injected into the UPLC-Q-TOF-MS system.

### 2.7. Microbial Transformation of Euphorbiasteroid

The biotransformation process was conducted at two scales: preliminary screening and preparative. Preliminary screening scale biotransformation of euphorbiasteroid was carried out in 250 mL Erlenmeyer flasks containing 100 mL of liquid medium. The flasks were placed on a rotary shaker (160 rpm, 28 °C). A standard two-stage fermentation protocol was employed in all experiments [29,30]. After 2 days of pre-culture, the substrates of 5 mg (dissolved in 0.5 mL of acetone) were added into each flask. Taking 1 mL samples on days 0, 2, 4, 7, 10, and 14, samples were centrifuged and the degree of transformation was compared to controls on TLC and HPLC, and a 2-μL aliquot of the supernatant was directly injected into the UPLC-Q-TOF-MS system. Culture controls consisted of sterile medium, in which microorganisms were grown under identical conditions without substrate. Substrate controls were composed of sterile medium and the same amount of substrate incubated under the same conditions without microorganisms.

### 2.8. Preparation of the Transformation Products of Euphorbiasteroid

The preparative scale biotransformation of euphorbiasteroid was carried out in 50 1000 mL Erlenmeyer flasks, each containing 400 mL of sterilized potato medium. The flasks were placed on a rotary shaker operating at 160 rpm at 28 °C. After 48 h of pre-culture, 20 mg of substrates in 2 mL of acetone were added to each flask. After 12 days of incubation, the culture was pooled and filtered. The filtrate was extracted three times with an equal volume of EtOAc and concentrated under reduced pressure to dryness.

The crude extract (3.58 g) was partitioned by MPLC column chromatography (CC) eluted with gradient MeOH/H_2_O (100% H_2_O, 25 mL/min, 3 h; 30% MeOH, 25 mL/min, 3 h; 50–70% MeOH, 25 mL/min, 3 h; 70–100% MeOH, 25 mL/min, 3 h) into 6 fractions (Fr. A-Fr. F). On the basis of TLC and HPLC analysis as well as comparing the LC-MS/MS data with those of the metabolites in rats, the metabolites of euphorbiasteroid were detected in Fr. B-Fr. E. Then, Fr. B (285.9 mg) was separated by Sephadex LH-20 CC (3 × 150 cm) with MeOH/H_2_O (30%) as eluent to give the fractions Fr. B1-Fr. B4. Next, Fr. B2 (64.5 mg) was purified by semi-preparative HPLC on a Zorbax SB-C18 semi-preparative column with the mobile phase consisting of methanol and 0.1% formic acid water (42:58, *v*/*v*) to obtain compound **9** (4.7 mg, *t*_R_ = 24.5 min). Fr. C (56.2 mg) was applied to Sephadex LH-20 CC (3 × 150 cm) eluting with MeOH/H_2_O (50%) to yield Fr. C1-Fr. C3. Then, Fr. C2 (10.2 mg) was further separated using semi-preparative HPLC (50% MeOH in water, *v*/*v*, 2.0 mL/min) to give compound **6** (2.9 mg, *t*_R_ = 51.5 min). Fr. D (78.9 mg) was purified by Sephadex LH-20 CC (3 × 150 cm) eluting with MeOH/H_2_O (50%) to afford four fractions (Fr. D1-Fr. D4). Then, Fr. D2 (40.4 mg) was further separated using semi-preparative HPLC (35% CH_3_CN in water, *v*/*v*, 2.0 mL/min) to give compounds **2** (1.7 mg, *t*_R_ = 58.6 min) and **5** (3.6 mg, *t*_R_ = 62.3 min). Fr. E (345.0 mg) was subjected to Sephadex LH-20 CC (3 × 150 cm) eluting with MeOH/H_2_O (50%) to provide five fractions (Fr. E1- Fr. E5). Then, Fr. E2 (30.3 mg) was submitted to semi-preparative HPLC eluting with MeOH-H_2_O (60:40, *v*/*v*, 2.0 mL/min) to yield compound **12** (10.6 mg, *t*_R_ = 31.5 min). Fr. E3 (38.5 mg) was purified by semi-preparative HPLC eluting with MeOH-H_2_O (65:35, *v*/*v*, 2.0 mL/min) to obtain compounds **1** (7.1 mg, *t*_R_ = 19.2 min), **4** (2.5 mg, *t*_R_ = 30.5 min), and **3** (2.0 mg, *t*_R_ = 37.0 min). Compounds **7** (1.7 mg, *t*_R_ = 26.5 min) and **8** (4.3 mg, *t*_R_ = 38.5 min) were acquired by semi-preparative HPLC (MeOH/H_2_O, 65%, 2 mL/min) from Fr. E4 (19.2 mg).

Compounds **10** and **11** were obtained by the hydrolysis of euphorbiasteroid. 210 mg of euphorbiasteroid was dissolved in 45 mL of MeOH, and 3 mL of 1.54 mol/L KHCO_3_ aqueous solution was added dropwise to this solvent with stirring. The mixture was stirred and hydrolyzed at 30 °C. After 5 days, methanol was removed by evaporation under vacuum, and the remaining solution was then extracted three times with 200 mL of solution (ethyl acetate: water = 1:1). The combined organic layer was evaporated under a vacuum. The ethyl acetate extract (184.5 mg) was re-dissolved in 10 mL of methanol, followed by Sephadex LH-20 CC (3 × 150 cm) eluting with MeOH/H_2_O (50%) to afford four fractions (Fr. 1-Fr. 5). Fr. 2 was further purified using semi-preparative HPLC (50% MeOH in water, *v*/*v*, 2.0 mL/min) to afford compounds **10** (44.6 mg, *t*_R_ = 5.5 min) and **11** (45.7 mg, *t*_R_ = 19.5 min).

### 2.9. Cell Culture and Cell Cytotoxicity Assay

Cell cytotoxicity was determined by the CCK-8 assay. Four strains of human cells (SH-SY5Y) were seeded in 96-well plates at a density of 3 × 10^3^ cells/well under 37 °C and 5% CO_2_ for 12 h and subsequently treated with the test sample solution (euphorbiasteroid and its metabolites, 10 μL) for 72 h. After treatment, each well with 10 μL CCK-8 reagent was incubated for 1–2 h in the incubator. Afterwards, the optical OD-value was measured at 450 nm through a microplate reader. Three multiple wells were set as parallel experimental groups.

## 3. Results and Discussion

### 3.1. Mass Fragmentation Behavior Analyses of Euphorbiasteroid

**Figure 2:**
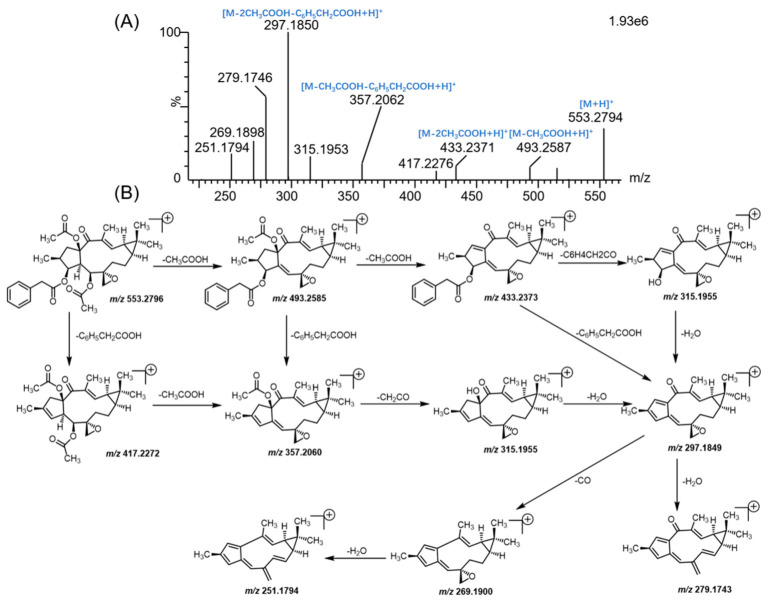
The mass spectrum (**A**) and proposed MS/MS fragmentation patterns (**B**) of euphorbiasteroid.

In order to obtain the overall fragmentation profile of euphorbiasteroid, the standard solution of euphorbiasteroid was analyzed by UPLC-Q/TOF-MS, which is helpful to better understand the MS/MS spectrum of its metabolites. The parent drug euphorbiasteroid had a protonated molecular ion [M + H]^+^ at *m*/*z* 553.2809 with a retention time of 12.67 min. In the MS/MS spectrum, it had the characteristic and most abundant fragment ion at *m*/*z* 297.1850, derived from the loss of two CH_3_COOH and one C_6_H_5_CH_2_COOH neutral molecules, which was further fragmented to form ion peaks at *m*/*z* 279.1746, *m*/*z* 269.1898, and *m*/*z* 251.1794 via loss of H_2_O, CO, and CO + H_2_O, respectively. Moreover, the fragment ions at *m*/*z* 493.2587 and *m*/*z* 417.2276 were produced by losing CH_3_COOH and C_6_H_5_CH_2_COOH from the ion at *m*/*z* 553.2794, respectively, which further yielded the fragment ions at *m*/*z* 433.2371 ([M + H-2CH_3_COOH]^+^) and *m*/*z* 357.2062 ([M + H-CH_3_COOH-C_6_H_5_CH_2_COOH]^+^). In addition, the fragment ion at *m*/*z* 315.1953 resulted from the ions at *m*/*z* 433.2371 and *m*/*z* 357.2062 by loss of C_6_H_4_CH_2_CO and CH_2_CO, respectively, which further lost a water to form ion at *m*/*z* 297.1850. Therefore, CH_3_COOH (*m*/*z* 493.2587), C_6_H_5_CH_2_COOH (*m*/*z* 417.2276), CH_3_COOH + C_6_H_5_CH_2_COOH (*m*/*z* 433.2371) and 2 CH_3_COOH + C_6_H_5_CH_2_COOH (*m*/*z* 279.1746) were the characteristic product ions of euphorbiasteroid. Mass spectra and the fragmentation scheme for euphorbiasteroid were shown in Figure 2. The ^1^H NMR and ^13^C NMR spectral data of euphorbiasteroid are listed in Table 1, Table 2 and Table 3, with the carbon position labeled as shown in Figure 1.

### 3.2. Identification of Metabolites of Euphorbiasteroid In Vitro and In Vivo

**Figure 3:**
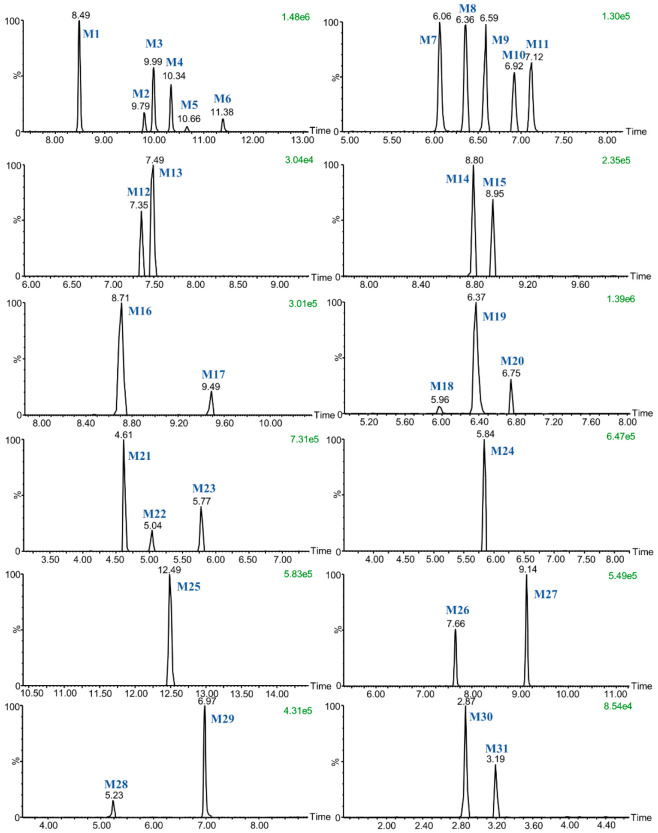
Extracted ion chromatograms of euphorbiasteroid metabolites in rats.

**Figure 4:**
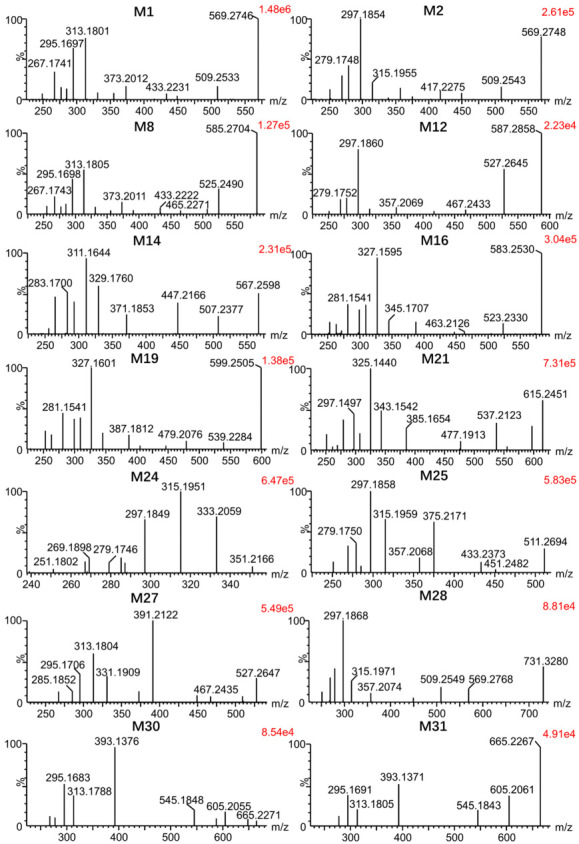
Product ion spectra of euphorbiasteroid metabolites in rats.

First, the metabolites of euphorbiasteroid in rats (plasma, urine, and feces), RLMs, and *C. elegans* culture medium were predicted by setting the prototype components and potential biological metabolic reactions in UNIFI 4.1 software. Then, the predicted metabolites in each sample were further compared according to the characteristic mass spectrum behaviors (including parent ions, internal cleavage in the ion source, and characteristic fragment ions of each metabolite) and retention times. A total of 31 metabolites identified in vitro and in vivo are listed in Table 4. The retention times, precursor molecular ions, and key fragments of euphorbiasteroid and its metabolites are listed in Table 4. The extracted ion chromatograms and product ion spectra of metabolites are shown in Figure 3 and Figure 4.

Metabolites **M1**, **M3**, and **M6** were detected at 8.49, 9.99, and 11.38 min, respectively. Taking **M1** as an example (Figure 4), the molecular ion at *m*/*z* 569.2746 ([M + H]^+^) was observed, with a 16 Da mass shift attributed to an oxygen atom relative to the substrate euphorbiasteroid (**M0**). The fragment ions at *m*/*z* 509.2533 and *m*/*z* 449.2336 resulted from successive CH_3_COOH loss from *m*/*z* 569.2746, and the fragment ions at *m*/*z* 433.2231, *m*/*z* 373.2012, and *m*/*z* 313.1801 were generated by C_6_H_5_CH_2_COOH loss from the ions at *m*/*z* 569.2746, *m*/*z* 509.2533, and *m*/*z* 449.2336, respectively. Furthermore, the ion at *m*/*z* 313.1801 was fragmented to form ions at *m*/*z* 295.1697, *m*/*z* 277.1595 and *m*/*z* 267.1741 via loss of H_2_O, 2H_2_O and CO + H_2_O, respectively. Moreover, the proposed fragmentation pathways of metabolites **M3** and **M6** were similar to those of metabolite **M1**, which were more likely to lose a H_2_O group compared with **M0**. This clearly suggested that the hydroxylated site should be located on the methyl moiety of the molecule, but the exact substituted position remained to be determined.

Metabolites **M2**, **M4**, and **M5** were eluted at 9.79, 10.34, and 10.66 min. Taking **M2** as an example (Figure 4), the molecular ion at *m*/*z* 569.2748 ([M + H]^+^) was observed, with a 16 Da mass shift attributed to an oxygen atom. The fragment ions at *m*/*z* 509.2543 and *m*/*z* 449.2332 resulted from successive CH_3_COOH loss from the molecular ion at *m*/*z* 569.2748, and the fragment ions at *m*/*z* 375.2172 and *m*/*z* 315.1955 were generated by the loss of C_6_H_5_CHCOO from the ions at *m*/*z* 509.2543 and *m*/*z* 449.2332, respectively. In particular, the fragment ion at *m*/*z* 417.2275 resulted from the molecular ion at 569.2748 by losing C_6_H_5_CH_2_COOH + O. Furthermore, the ion at *m*/*z* 315.1955 was fragmented to form ions at *m*/*z* 297.1854, *m*/*z* 279.1748, and *m*/*z* 269.1904 via loss of H_2_O, 2H_2_O, and CO + H_2_O, respectively. Moreover, metabolites **M2**, **M4**, and **M5** were more likely to lose a H_2_O group compared with euphorbiasteroid. The loss of C_6_H_5_CH_2_COOH + O clearly suggested that the hydroxylated site should be located on the 3-O-phenylacetyl moiety of the molecule, but the exact substituted position remained to be determined.

Metabolites **M7**–**M11** were predicted to be dihydroxylated derivatives with HPLC retention times at 6.06 and 7.12 min. For example, the molecular ion of **M8** was detected at *m*/*z* 585.2704 ([M + H]^+^) in positive ion mode, 16 mass units more than those of **M1**–**M6**, suggesting the presence of an additional hydroxyl group (Figure 4). The fragment ions at *m*/*z* 525.2490 and *m*/*z* 465.2271 resulted from successive CH_3_COOH loss of the parent ion at *m*/*z* 585.2704, and the ion at *m*/*z* 465.2271 was further fragmented to the ion at *m*/*z* 313.1805 by the loss of C_6_H_5_CH_2_COOH + O, in which the ion at *m*/*z* 433.2222 resulted from *m*/*z* 585.2704 in a similar mechanism. The fragment ions at *m*/*z* 373.2011 and *m*/*z* 313.1805 were generated by successive CH_3_COOH loss from the ion at *m*/*z* 433.2222. Furthermore, the ion at *m*/*z* 313.1805 was further fragmented to form the ions at *m*/*z* 295.1698 and *m*/*z* 285.1847 by the loss of H_2_O and CO, respectively. Consistent with the monohydroxylated products of euphorbiasteroid, these showed a series of product ions resulting from the loss of CH_3_COOH and C_6_H_5_CH_2_COOH. This indicates that metabolites **M7**–**M11** may be produced by further hydroxylation on the basis of metabolites **M1**–**M6**.

Metabolites **M12** and **M13** were detected with HPLC retention times between 7.35 and 7.49 min. Taking **M12** as an example (Figure 4), the molecular ion at *m*/*z* 587.2858 ([M + H]^+^) was observed, 34 Da higher than that of euphorbiasteroid. The fragment ions at *m*/*z* 527.2645 and *m*/*z* 467.2433 resulted from successive CH_3_COOH loss of the parent ion at *m*/*z* 587.2858, and the ion at *m*/*z* 527.2645 was fragmented to the ion at *m*/*z* 357.2069 following the loss of C_6_H_5_CH_2_COOH + H_2_O + O. Furthermore, the fragment ion at *m*/*z* 297.1860 was formed via the elimination of CH_3_COOH from the ion at *m*/*z* 357.2069, and the fragment ions at *m*/*z* 279.1752 and 269.1905 were produced by the loss of H_2_O and CO from the ion at *m*/*z* 297.1860, respectively. The MS/MS spectra of **M12** and **M13** were similar to those of **M7**–**M11**, except that the corresponding ions were each heavier by 2 Da. Therefore, it was provisionally presumed that **M12** and **M1**3 were produced via changing the olefin of euphorbiasteroid into dihydrodiol.

The metabolites **M14** and **M15** were eluted between 8.80 and 8.95 min, and showed the protonated ion at *m*/*z* 567.2598 ([M + H]^+^). They were 14 Da more than euphorbiasteroid and 2 Da less than **M1**–**M6** (Figure 4). Taking **M14** as an example, the fragment ions at *m*/*z* 507.2377 and *m*/*z* 447.2166 resulted from successive CH_3_COOH loss from *m*/*z* 567.2598, while the fragment ions at *m*/*z* 371.1853 and *m*/*z* 311.1644 were formed via the elimination of C_6_H_5_CH_2_COOH from the ion at *m*/*z* 507.2377 and *m*/*z* 447.2166, respectively. Furthermore, the fragment ions at *m*/*z* 293.1538 and *m*/*z* 283.1700 also resulted from H_2_O and CO loss from the ion at *m*/*z* 311.1644, respectively. In particular, the fragment ions at *m*/*z* 283.1700 and *m*/*z* 255.1747 were generated by successive loss of CO from the ion at *m*/*z* 311.1644, indicating the existence of an aldehyde group. Furthermore, according to the proposed metabolic pathway of tanshinone IIA [31], the methyl group of euphorbiasteroid might undergo similar metabolic modification, from methyl to primary alcohol, and then to an aldehyde group. Thus, it is provisionally interpreted that **M10** and **M11** were produced by transforming one methyl of euphorbiasteroid to aldehyde.

The metabolites **M16** and **M17**, which showed protonated molecular ions at *m*/*z* 583.2530 ([M + H]^+^) with the retention times of 8.71 and 9.49 min, were 30 Da higher than euphorbiasteroid and 2 Da less than the dihydroxylation products of euphorbiasteroid like **M7**–**M11** (Figure 4). Taking **M16** as an example, the fragment ions at *m*/*z* 523.2330 and *m*/*z* 463.2126 were proposed to result from successive loss of CH_3_COOH from the molecular ion *m*/*z* 583.2530. In addition, the fragment ions at *m*/*z* 447.2032, *m*/*z* 387.1815, and *m*/*z* 327.1595 were generated from the ions at *m*/*z* 583.2530, *m*/*z* 523.2330 and *m*/*z* 463.2126 through the loss of C_6_H_5_CH_2_COOH, respectively. The ion at *m*/*z* 327.1595 was further fragmented into the ions at *m*/*z* 309.1498 and *m*/*z* 299.1645 via the loss of H_2_O and CO, respectively. In particular, the fragment ions at *m*/*z* 281.1541, *m*/*z* 263.1438, and *m*/*z* 253.1591 were formed via the elimination of HCOOH from the ions at *m*/*z* 327.1595, *m*/*z* 309.1498, and *m*/*z* 299.1645, respectively, which indicated the existence of a carboxyl group. The compounds might be produced by the oxidation of **M14** or **M15**, which was consistent with our initial speculation. Thus, it is provisionally interpreted that **M16** and **M17** were produced by oxidizing the aldehyde of **M14** or **M15** into carboxyl groups.

Metabolites (**M18**–**M20**) were detected between 5.96 and 6.75 min. Taking **M19** as an example (Figure 4), it showed a molecular ion at *m*/*z* 599.2505 ([M + H]^+^), with 16 Da higher than the metabolites **M16** and **M17**. The fragment ions at *m*/*z* 539.2284 and *m*/*z* 479.2076 resulted from successive CH_3_COOH loss from the parent ion at *m*/*z* 599.2505, while the fragment ions at *m*/*z* 447.2024, *m*/*z* 387.1812, and *m*/*z* 327.1601 were proposed to result from loss of C_6_H_5_CH_2_COOH + O from the ions at *m*/*z* 599.2505, *m*/*z* 539.2284, and *m*/*z* 479.2076, respectively. Additionally, a battery of fragment ions at *m*/*z* 309.1494, *m*/*z* 299.1647, *m*/*z* 281.1541, *m*/*z* 263.1436 and *m*/*z* 253.1585 were produced, which were consistent with the fragmentation behaviors of the metabolites **M16** and **M17**. Therefore, these metabolites were provisionally characterized as monohydroxylated products of the metabolites **M16** and **M17**.

The metabolites (**M21**–**M23**) were eluted at between 4.61 and 5.77 min, giving rise to the protonated molecules [M + H]^+^ at *m*/*z* 615.2451. Taking **M21** as an example (Figure 4), the fragment ion at *m*/*z* 615.2451 was fragmented into the ions at *m*/*z* 555.2226, *m*/*z* 537.2123, and *m*/*z* 477.1913 following the loss of CH_3_COOH, CH_3_COOH + H_2_O, and 2CH_3_COOH + H_2_O, respectively. The fragment ions at *m*/*z* 385.1654 and *m*/*z* 325.1440 were proposed to result from loss of C_6_H_5_CH_2_COOH + O from the ions at *m*/*z* 537.2123 and *m*/*z* 477.1913, respectively. In particular, the MS/MS characteristic ions of the metabolite **M21** (*m*/*z* 385.1654, 325.1440, 307.1334, 297.1497, 279.1386, 261.1280, and 251.1426) were 2 Da less than those of the metabolites **M16** and **M17**. Therefore, they were tentatively identified as dihydroxylated products of metabolites **M16** and **M17**.

The metabolite **M24** was observed as its protonated molecular ion[M + H]^+^ at *m*/*z* 351.2166 with a retention time of 5.84 min, which was deductively assigned as a hydrolysis product of euphorbiasteroid because its protonated ion was 202 Da less than that of euphorbiasteroid (Figure 4). The fragment ions at *m*/*z* 333.2059, *m*/*z* 315.1951, *m*/*z* 297.1849, and *m*/*z* 279.1746 resulted from successive H_2_O loss from the parent ion at *m*/*z* 351.2166. Furthermore, a battery of fragment ions at *m*/*z* 315.1951, *m*/*z* 297.1849, *m*/*z* 279.1746, *m*/*z* 269.1898, and *m*/*z* 251.1802 were produced, which were consistent with fragmentation behaviors of euphorbiasteroid. Therefore, the metabolite **M24** was probably formed via the loss of two CH_2_CO and a C_6_H_5_CHCO from the prototype compound euphorbiasteroid. Similar findings have been reported before [32].

The metabolite **M25** provided its protonated ion at *m*/*z* 511.2694 with a retention time of 12.49 min (Figure 4). It was 42 Da less than that of euphorbiasteroid, meaning that it might be a hydrolysis product of euphorbiasteroid. In the MS/MS spectrum, a battery of fragment ions at *m*/*z* 451.2482 [M + H-CH_3_COOH]^+^, *m*/*z* 433.2373 [M + H-CH_3_COOH-H_2_O]^+^, *m*/*z* 375.2171 [M + H-C_6_H_5_CH_2_COOH]^+^, and *m*/*z* 297.1858 [M + H-CH_3_COOH-H_2_O-C_6_H_5_CH_2_COOH]^+^ were observed, suggesting that the metabolite **M25** was produced by losing an acetyl of euphorbiasteroid.

The metabolites **M26** and **M27**, which showed their respective positive ions at *m*/*z* 527.2661 and 527.2647 with the retention times of 7.66 and 9.14 min, were plausibly assigned as monohydroxylated products of metabolite **M25** due to their protonated ions being 16 Da more than that of **M25** (Figure 4). In the MS/MS spectrum of **M27**, the characteristic fragment ions at *m*/*z* 467.2435 [M + H-CH_3_COOH]^+^, *m*/*z* 391.2122 [M + H-C_6_H_5_CH_2_COOH]^+^, and *m*/*z* 331.1909 [M + H-CH_3_COOH-C_6_H_5_CH_2_COOH]^+^ were detected, which was in accordance with the mass fragmentation behaviors of **M25**. In addition, the fragment ions at *m*/*z* 313.1804 [M + H-CH_3_COOH-C_6_H_5_CH_2_COOH-H_2_O]^+^ and *m*/*z* 295.1706 [M + H-CH_3_COOH-C_6_H_5_CH_2_COOH-2H_2_O]^+^ proved the existence of hydroxylation. According to the reliable analysis above, they were provisionally characterized as monohydroxylated products of **M25**.

The metabolites **M28** and **M29** gave rise to protonated ions at *m*/*z* 731.3280 and 731.3286, which were eluted at 5.23 and 6.97 min, respectively. They were 162 Da more than metabolites **M1**–**M6**. Taking **M28** as an example (Figure 4), the fragment ion at *m*/*z* 569.2768 provided reliable evidence for identifying the metabolites as glycosylation products of euphorbiasteroid due to the loss of 162 mass units. The fragment ions at *m*/*z* 509.2549, 449.2335, 357.2074, 315.1971, 297.1868, 279.1754, and 269.1917 demonstrated that a hydroxyl group in the metabolites **M7**–**M11** was replaced by C_6_H_10_O_5_. Therefore, they were tentatively identified as glycosylation products of euphorbiasteroid.

The metabolites **M30** and **M31** were observed with their protonated ions at *m*/*z* 665.2271 and 665.2267 with retention times of 2.87 and 3.19 min, which were deductively assigned as sulfonated products of the metabolites **M7**–**M11** because their protonated ions were 80 Da larger than the metabolites **M7**–**M11** (Figure 4). In the MS/MS spectrum of **M3**0, the characteristic ions at *m*/*z* 605.2055 [M + H-CH_3_COOH]^+^, *m*/*z* 545.1848 [M + H-2CH_3_COOH]^+^, and *m*/*z* 393.1376 [M + H-2CH_3_COOH-C_6_H_5_CH_2_COOH-O]^+^ were consistent with fragmentation characteristics of euphorbiasteroid, which provided reliable reference for the identification of **M30**. Particularly, the fragment ion at *m*/*z* 313.1788 was proposed to result from the loss of SO_3_ from the ion at *m*/*z* 393.1376. Moreover, a battery of fragment ions at *m*/*z* 295.1683, *m*/*z* 277.1589, and *m*/*z* 267.1744 were also observed, suggesting that the metabolites **M30** and **M31** should be sulfonated products of metabolites **M7**–**M11**.

Finally, in order to explore whether RLMs and the microbial model could simulate the metabolism of euphorbiasteroid in rats, the incubation of RLMs and *C. elegans* bio-110930 with euphorbiasteroid were studied, respectively. The results showed that 18 and 14 metabolites identified in the above two models could match those of rats in vivo*,* respectively, indicating that they could simulate the metabolism of euphorbiasteroid in vivo to a certain extent. Considering the economic benefits and transformation efficiency, the large-scale microbial transformation experiment was used for the subsequent preparation of metabolite standards and the accurate characterization of the metabolite structures [33,34,35].

### 3.3. The Structure Elucidation of Transformation Products

**Figure 5:**
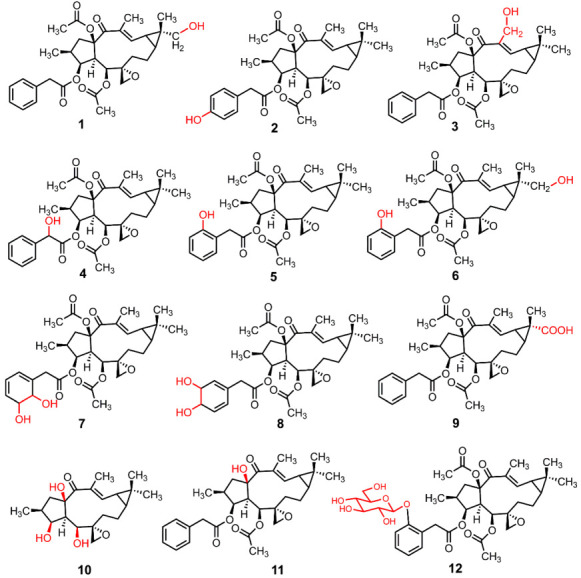
The structures of euphorbiasteroid and its transformation products.

Through large-scale microbial transformation and chemical hydrolysis experiments, twelve transformation products (**1–12**) were prepared, including eleven new compounds (**1**–**9**, **11** and **12**) and one known compound (**10**). Compound **10** was characterized as epoxylathyrol by comparison of the NMR and HR-ESI-MS data with the literature [36]. The structures of eleven new transformation products were characterized by ESI-MS, HR-ESI-MS, 1D-, and 2D-NMR data (Figure 5).

**Figure 6:**
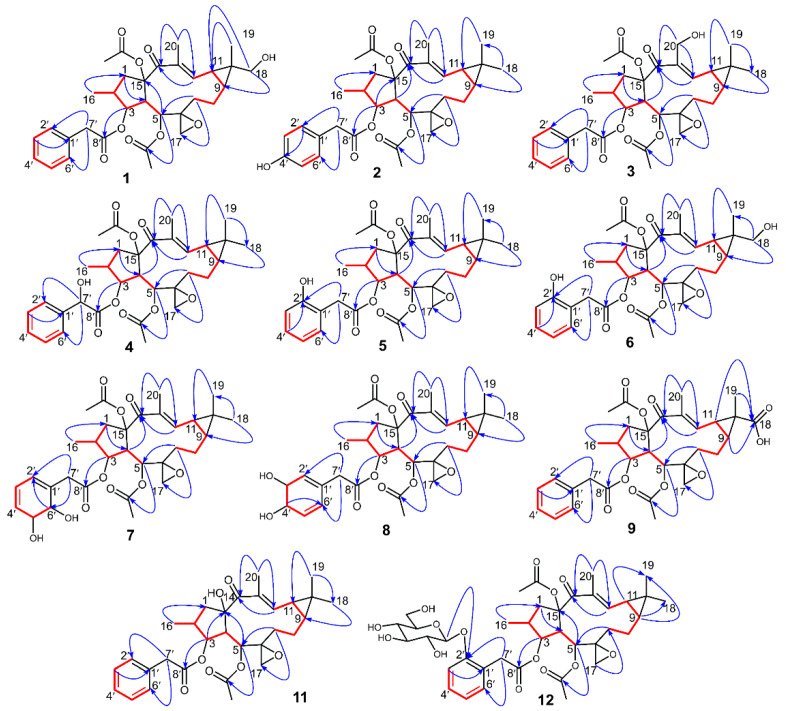
1H-1H COSY (━) and HMBC (→) correlations of compounds **1**–**9** and **11**–**12**.

**Figure 7:**
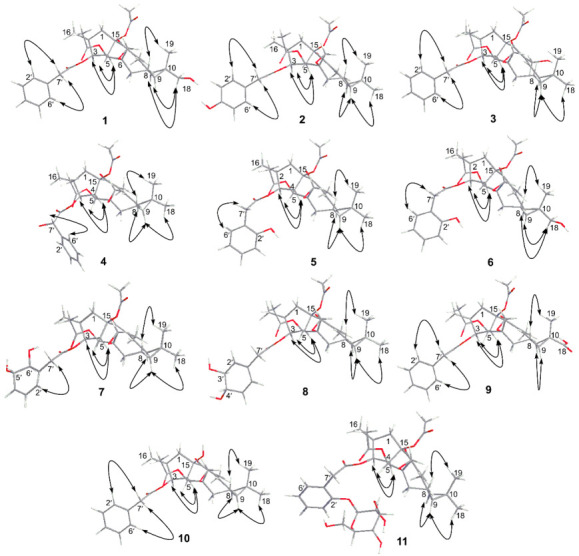
NOESY (↔) correlations of compounds **1**–**9** and **11**–**12**.

Compound **1** was isolated as a white powder with an optical rotation of ${\left[\alpha \right]}_{D}^{20}$ +116.10° (*c* 0.118, MeOH). The molecular formula of C_32_H_40_O_9_ was deduced from the [M + H]^+^ ion at *m*/*z* 569.2746, and the molecular weight of compound **1** is 16 Da more than that of the precursor compound euphorbiasteroid. Analysis of the ^1^H and ^13^C NMR spectroscopic data of 1 revealed its structural similarity to euphorbiasteroid (**M0**, Table 1 and Table 3). The only difference between these two compounds was the occurrence of an extra hydroxymethyl [*δ*_C_ 71.2, *δ*_H_ 3.52 (d, *J* = 11.25 Hz, H-18a)] and *δ*_H_ 3.44 (d, *J* = 11.25 Hz, H-18b)) in compound **1**, taking the place of a methyl of euphorbiasteroid. In the HMBC spectrum of compound **1**, the two protons of hydroxymethyl at *δ*_H_ 3.52 and 3.44 exhibited long-range HMBC correlations with C-9 (*δ*_C_ 30.2) and C-11 (*δ*_C_ 25.2) (Figure 6), suggesting that a hydroxyl might be substituted at C-18 or C-19 position. In the NOESY spectrum (Figure 7), the key NOE correlations of two hydroxymethyl protons (*δ*_H_ 3.52 and 3.44) with H-9 (*δ*_H_ 1.21) and H-11 (*δ*_H_ 1.65) proved that the C-18 of compound **1** was substituted by a hydroxyl, and assigned the relative configuration of 18-hydroxymethyl to be α-oriented. Unambiguous complete assignments for the ^1^H and ^13^C NMR signals were made by combination of DEPT, ^1^H-^1^H COSY, HSQC, HMBC, and NOESY spectra (Figures S1–S10). On the basis of the above evidence, the structure of compound **1** was thus established as 18α-hydroxyl euphorbiasteroid.

Compound **2** was isolated as a white powder with ${\left[\alpha \right]}_{D}^{20}\;$+74.89° (*c* 0.150, MeOH). The molecular formula was assigned as C_32_H_40_O_9_ based on its HR-ESI-MS data that displayed an [M + H]^+^ ion at *m*/*z* 569.2747. Similar to compound **1**, the molecular weight of **2** was 16 mass units more than that of euphorbiasteroid and shared the same molecular formula. The ^1^H and ^13^C NMR data of compound **2** were quite close to those of euphorbiasteroid (Table 1 and Table 3), except for the absence of an aromatic proton. In the ^1^H NMR spectrum of **1**, two groups of aromatic protons (each two protons) supported the presence of a *p*-substituted benzyl ring. Two groups of carbon resonances (each two carbons) at *δ*_C_ 130.6 and 115.4 as well as the ^13^C chemical shift of C-4′ proved the above deduction, and attributed a hydroxyl substitution at C-4′ (Figures S11–S20). On the basis of the above evidence, the structure of compound **2** was established as 4′-hydroxyl euphorbiasteroid.

Compound **3**, a white powder, had an optical rotation of ${\left[\alpha \right]}_{D}^{20}\;$+63.97° (*c* 0.130, MeOH). Its molecular formula was deduced to be C_32_H_40_O_9_ from the [M + H]^+^ ion at *m*/*z* 569.2746 in HRESIMS. Compound **3** was proposed to be a hydroxylated product of euphorbiasteroid due to its molecular weight being 16 mass units more than that of euphorbiasteroid. When comparing its ^1^H and ^13^C NMR data with those of euphorbiasteroid (Table 1 and Table 3), it was revealed that the two compounds shared a great similarity. In the NMR spectra, the appearance of an extra oxygenated methylene unit [*δ*_H_ 4.28 (d, *J* = 12.38 Hz, H-20a) and *δ*_H_ 4.42 (d, *J* = 12.38 Hz, H-20b); *δ*_C_ 58.1], in place of the methyl signals for C-20 (*δ*_H_ 1.82; *δ*_C_ 12.3) in euphorbiasteroid, suggested that one hydroxyl was incorporated to **3** at C-20 position. Key HMBC correlations of two protons of the hydroxymethyl (*δ*_H_ 4.28 and 4.42) with C-14 (*δ*_C_ 198.4) and C-12 (*δ*_C_ 147.6) attached a hydroxyl to C-20 (Figure 6). Assignments of the ^1^H and ^13^C NMR signals were achieved by a combination of DEPT, ^1^H-^1^H COSY, HSQC, and HMBC experiments (Figures S21–S30). Thus, the structure of compound **3** was identified as 20-hydroxyl euphorbiasteroid.

Compound **4** was obtained as a white powder with ${\left[\alpha \right]}_{D}^{20}$ +70.00° (*c* 0.125, MeOH). The protonated ion [M + H]^+^ at *m*/*z* 569.2756 (calcd for C_32_H_41_O_9_, 569.2745) in HRESIMS assigned the molecular formula to be C_32_H_40_O_9_ The ^1^H and ^13^C NMR data of **4** closely matched those of euphorbiasteroid (Table 1 and Table 3) The only difference was the appearance of the signals for an oxygenated methine (*δ*_H_ 5.10, *δ*_C_ 72.8) in **4**, rather than the C-7′ methylene signals (*δ*_H_ 3.58, *δ*_C_ 41.5). Thus, it was speculated that compound **4** was a C-7′ hydroxylated product of euphorbiasteroid. In addition, relative to euphorbiasteroid, the chemical shifts of C-1′ and C-8′ of compound 4 were downfield shifted significantly from *δ*_C_ 133.8 to *δ*_C_ 138.4, and from *δ*_C_ 170.9 to *δ*_C_ 173.7, respectively, while the resonances of C-2′ and C-6′ was upfield shifted from *δ*_C_ 129.4 to *δ*_C_ 126.5. The key HMBC correlations from H-7′ at *δ*_H_ 5.10 to C-1′, C-2′, C-6′ and C-8′ (Figure 6) further proved that a hydroxyl was substituted at C-7′ position. The stereo configuration of 7′-OH failed to be determined due to a lack of substantial NOE correlation (Figures S31–S40). Therefore, compound **4** was assigned as 7′-hydroxyl euphorbiasteroid.

Compound **5** was isolated as a white powder with the optical rotation ${\left[\alpha \right]}_{D}^{20}$ +112.65° (*c* 0.108, MeOH). The molecular formula was inferred as C_32_H_40_O_9_ due to the appearance of an [M + H]^+^ ion at *m*/*z* 569.2754 in the HR-ESI-MS spectrum. In the ^1^H and ^13^C NMR spectra of **5** (Table 1 and Table 3), compared to euphorbiasteroid, it was observed to have one additional oxygenated quaternary carbon at *δ*_C_ 154.7, replacing an aromatic methine of the benzyl ring. Additionally, the ^1^H NMR spectrum exhibited four aromatic proton signals for an AA′BB′ coupling system at *δ*_H_ 6.90, 7.18, 6.88, and 7.11. The above information, in combination with key HMBC correlations from H_2_-7′ (*δ*_H_ 3.70 and 3.55) to C-2′ (*δ*_C_ 154.7) and C-6′ (*δ*_C_ 131.1), supported that a hydroxyl was substituted at the C-2′ of the benzyl ring. Unambiguously complete assignments for the ^1^H and ^13^C NMR signals were made by a combination of DEPT, ^1^H-^1^H COSY, HSQC, and HMBC spectra (Figures S41–S50). Thus, compound **5** was identified as 2′-hydroxyl euphorbiasteroid.

The molecular formula of compound **6** was deduced to be C_32_H_40_O_10_ from the HR-ESI-MS [M + H]^+^ ion *m*/*z* 585.2704 (calcd for C_32_H_41_O_10_, 585.2694). Its molecular weight was 16 mass units more than that of compound **5**, implying that compound **6** might be a dihydroxylated product of euphorbiasteroid. The ^1^H and ^13^C NMR data closely resembled those of **5** (Figures S51–S60). The only difference was that compound **6** had an extra hydroxymethyl (*δ*_H_ 3.48, 3.38; *δ*_C_ 70.9), instead of a methyl (*δ*_H_ 1.20; *δ*_C_ 28.9) of **5**. In the HMBC spectrum (Figure 6), key long-range correlations from two hydroxymethylene protons at *δ*_H_ 3.48 and 3.38 to C-9 (*δ*_C_ 31.1) and C-11 (*δ*_C_ 26.2) revealed that a hydroxyl was linked to C-18 or C-19 position. The observation of NOE correlations (Figure 7) from the hydroxymethylene protons at *δ*_H_ 3.48 and 3.38 to H-9 (*δ*_H_ 1.33) and H-11 (*δ*_H_ 1.78) unambiguously attributed the hydroxymethylene as C-18, and determined the relative configuration of 18-CH_2_OH to be *α*-oriented. Therefore, the chemical structure of compound **6** was identified as 18α, 2′-dihydroxyl euphorbiasteroid.

Compound **7** was obtained as a white powder with an optical rotation ${\left[\alpha \right]}_{D}^{20}$ +60.00° (*c* 0.170, MeOH). Its molecular formula was deduced as C_32_H_42_O_10_ due to the [M + H]^+^ ion at *m*/*z* 587.2851 (calcd for C_32_H_43_O_10_, 587.2878) in HR-ESI-MS. Interestingly, the molecular weight of **7** was 34 mass units more than that of euphorbiasteroid, and had one less degree of unsaturation, suggesting that there was a possible missing double bond or ring as well as two hydroxyl substituents. The ^1^H and ^13^C NMR spectroscopic data (Table 2 and Table 3) showed that compound **7** shared most of its featured functionalities and had the same diterpene skeleton as euphorbiasteroid, but had a great difference in the phenylacetyl moiety. Only three aromatic proton signals occurred at *δ*_H_ 5.54, 7.54, and 6.07 in the ^1^H NMR spectrum, and four aromatic carbon resonances were observed at *δ*_C_ 150.2, 115.5, 125.5, and 138.1 in the ^13^C NMR spectrum. Detailed interpretation of the NMR spectroscopic data indicated that two additional oxygenated aliphatic methines [*δ*_H_ 4.26 (d, *J* = 7.49 Hz), 3.76 (m); *δ*_C_ 73.5, 72.9] in compound **7** replaced two aromatic methines [*δ*_H_ 7.30 (m), 7.25 (overlap); *δ*_C_ 128.5, 129.4] of euphorbiasteroid. The ^1^H-^1^H COSY correlations of H-2′ (*δ*_H_ 5.54)/H-3′ (*δ*_H_ 7.54)/H-4′ (*δ*_H_ 6.07)/H-5′ (*δ*_H_ 4.26)/H-6′ (*δ*_H_ 3.76) in combination with the key HMBC long-range correlations from H_2_-7′ (*δ*_H_ 2.67, 2.54) to C-2′ (*δ*_C_ 115.5) and C-6′ (*δ*_C_ 72.9) (Figure 6) evidenced that the C-5′ and C-6′ double bond of phenyl ring was hydrogenated and subsequently substituted by two hydroxyls, which was in agreement with the molecular weight and degrees of unsaturation of **7** as mentioned above. The stereo configurations of 5′-OH and 6′-OH failed to be determined due to a lack of substantial NOE correlations in the NOESY spectrum of compound **7** (Figures S61–S70). Based on the above analysis, the chemical structure of compound **7** was identified as 5′,6′-dihydroxyl dihydroeuphorbiasteroid.

Compound **8**, a yellowish powder with ${\left[\alpha \right]}_{D}^{20}$ +90.83° (*c* 0.123, MeOH), had the same molecular formula C_32_H_42_O_10_ and degrees of unsaturation as compound **7** due to the [M + H]^+^ ion peak at *m*/*z* 587.2873 (calcd for C_32_H_43_O_10_, 587.2878) in its HR-ESI-MS spectrum. The ^1^H and ^13^C NMR spectra (Table 2 and Table 3) demonstrated almost the same spectroscopic features as those of compound **7**, including a diterpenoid skeleton, two acetyl moieties, and a hydrogenated and dihydroxylated phenyl acetyl moiety [*δ*_H_ 5.66 (s), 3.72 (brs), 4.25 (brd., 5.56), 6.07, 6.15 (d, *J* = 10.14 Hz), 3.69, and 2.55; *δ*_C_ 151.0, 117.6, 72.3, 72.2, 137.1, 130.732.4, and 165.8], suggesting that compounds **7** and **8** might be isomers. Detailed analysis of the ^1^H and ^13^C NMR spectroscopic data revealed that compound **8** had a different hydrogenated and dihydroxylated position. In 2D NMR spectra, the ^1^H-^1^H COSY correlations of H-2′ (*δ*_H_ 5.66)/H-3′ (*δ*_H_ 3.72)/H-4′ (*δ*_H_ 4.25)/H-5′ (*δ*_H_ 6.07)/H-6′ (*δ*_H_ 6.15) implied that the C-3′ and C-4′ double bond of phenyl ring was hydrogenated and subsequently substituted by two hydroxyls, respectively. The above deduction was further proved by key HMBC correlations of H_2_-7′ (*δ*_H_ 3.69, 2.55) with the aromatic C-2′ (*δ*_C_ 117.6) and C-6′ (*δ*_C_ 130.7), and of H-4′ [*δ*_H_ 5.66 (brd., *J* = 5.56 Hz) with C-6′ (Figures S71–S80). Thus, the structure of compound **8** was identified as 3′,4′-dihydroxyl dihydroeuphorbiasteroid. The stereo configurations of 3′-OH and 4′-OH failed to be determined due to a lack of substantial NOE correlations.

Compound **9** was isolated as a white powder with an optical rotation of ${\left[\alpha \right]}_{D}^{20}$ +77.78° (*c* 0.123, MeOH). The HREI-MS exhibited the [M + H]^+^ ion at *m*/*z* 583.2330 (calcd. 583.2543), corresponding to the molecular formula C_32_H_38_O_10_. The molecular weight of **9** was 30 mass units more than that of the substrate euphorbiasteroid, implying that compound **9** might be a carboxylated product. Compared to the substrate euphorbiasteroid, the ^1^H and ^13^C NMR spectra (Table 2 and Table 3) exhibited an additional carboxyl signal (*δ*_C_ 179.8), but an absence of the signals for CH_3_-18 (*δ*_H_ 1.20; *δ*_C_ 28.9). Additionally, the chemical shift of C-19 was significantly shifted to high field by 6.4 ppm. These data implied that compound **9** might be a C-18 carboxylated product of euhporbiasteroid. This deduction was substantially proved by key HMBC correlations (Figure 6) from CH_3_-19 (*δ*_H_ 1.44), H-9 (*δ*_H_ 1.90), and H-11 (*δ*_H_ 2.44) to the carboxyl carbon (*δ*_C_ 179.8) (Figure S81–S90). Thus, the structure of compound **9** was thus identified as 18α-carboxyl euphorbiasteroid.

Compound **11** was isolated as a white powder with ${\left[\alpha \right]}_{D}^{20}$ +117.01° (*c* 0.146, MeOH). Its HR-ESI-MS spectrum gave a hydrogen adduct ion at *m*/*z* 511.2694, assigning the molecular formula of **11** as C_30_H_38_O_7_. The ^1^H and ^13^C NMR spectra of **11** exhibited quite similar spectroscopic features to those of **M0**, including four methyls [*δ*_H_ 0.81 (d, *J* = 6.69 Hz), 1.21 (s), 1.25 (s), 1.78 (s); *δ*_C_ 14.2, 29.0, 16.3, 12.2], one double bond [*δ*_H_ 7.32 (overlap), *δ*_C_ 150.8, 135.1], one ketone carbonyl (*δ*_C_ 199.3), one acetoxyl [*δ*_H_ 2.08 (3H, s), *δ*_C_ 171.0, 21.1], one oxygenated methine (*δ*_H_ 5.46; *δ*_C_ 82.8), and one oxygenated quaternary carbon (*δ*_C_ 88.7), and one characteristic three-membered epoxyl motif (*δ*_H_ 2.41 and 2.27, *δ*_C_ 58.9 and 55.4), with the exception of the disappearance of one acetoxyl in **11** (Table 2 and Table 3). The downfield shifted carbon signals at C-1 (Δ*δ* +2.0)/C-4 (Δ*δ* +1.7)/C-5 (Δ*δ* +0.8)/C-14 (Δ*δ* +2.4) and the upfield shifted C-15 (Δ*δ* -3.0) suggested that the acetoxyl at C-15 was replaced by one hydroxyl. Furthermore, the key HMBC correlations from the protons at *δ*_H_ 5.92 (H-5) and 5.46 (H-3) to C-15 (*δ*_C_ 88.7) further supported the above deduction (Figure 6). Interpretation of the NOESY spectrum revealed that compound **11** shared the same relative configurations as those of **M0** (Figure 7). Therefore, the structure of compound **11** was identified as 15-deacetyl euphorbiasteroid (Figures S94–S103).

The HR-ESI-MS spectrum of compound **12** gave a hydrogen adduct ion [M + H]^+^ *m*/*z* 731.3280 (calcd for C_38_H_50_O_14_, 731.3273), assigning the molecular formula as C_38_H_50_O_14_. Its molecular weight was 162 Da more than that of the substrate euphorbiasteroid, implying that compound **12** might be a hexosylated product of euphorbiasteroid. The ^1^H and ^13^C NMR spectroscopic data of compound **12** were very similar to those of the substrate euphorbiasteroid, and the major difference was the presence of an additional glucosyl unit [*δ*_H_ 4.81 and 3.49–3.89 (6H); *δ*_C_ 101.4, 75.9, 75.9, 73.5, 69.6, 61.8] in the structure of **12** (Table 2 and Table 3). The substitution position of glucosyl was unambiguously determined to be the C-2′ of the phenyl ring due to key HMBC correlations from the anomeric proton (*δ*_H_ 4.81) of glucosyl and H_2_-7′ (*δ*_H_ 3.67, 3.64) to C-2′ (*δ*_C_ 155.3) (Figure 6). The ^13^C chemical shifts of glucosyl and the large coupling constant of the anomeric proton (*J*_H-1__′′/H-2__′′_ = 6.77 Hz) attributed the glucosyl to be *β*-D-glucose. Assignment of the ^1^H and ^13^C NMR signals was achieved by a combination of DEPT, ^1^H-^1^H COSY, and HSQC experiments (Figure S104–S113). Based on this evidence, the structure of compound **12** was determined to be euphorbiasteroid 2′-*O-β-D*-glucopyranoside.

### 3.4. Comparison of Metabolite Formation In Vitro and In Vivo

**Figure 8:**
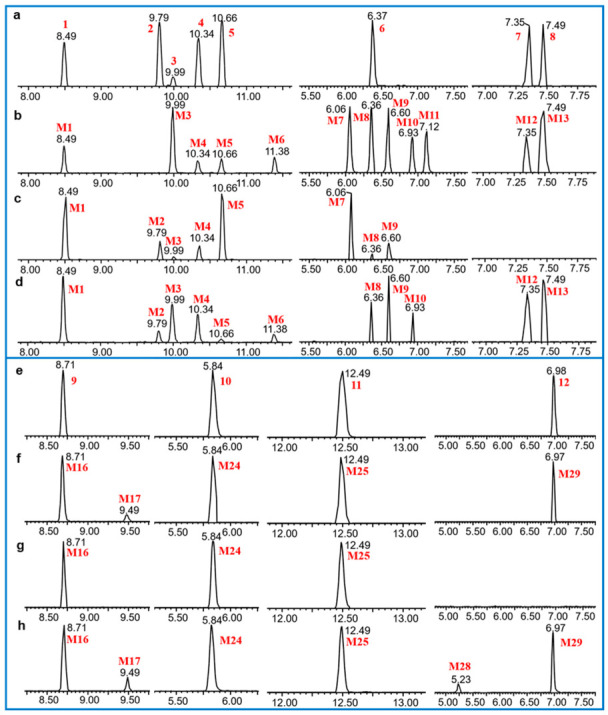
Extracted ion chromatograms of reference standard isolated from microbial transformation samples (**a**,**e**) and euphorbiasteroid metabolites in rat plasma (**b**,**f**), urine (**c**,**g**) and feces (**d**,**h**).

Various results of metabolite formation were discovered in the three approaches used in this study. Rat plasma, urine, and feces samples were generally rich in metabolites, with 27, 20, and 29 metabolites, respectively. In vitro incubation of RLMs produced the same 18 phase I metabolites as the metabolites in rats, just less in number and amount. In vitro co-incubation of *C. elegans* bio-110930 with euphorbiasteroid yielded 14 metabolites, 12 of which were further prepared by large scale microbial transformation and confirmed to be the same metabolites as those in rats by comparison of their HPLC retention times and MS/MS fragments (Figure 8), On the basis of analysis of 1D and 2D NMR spectroscopic data, 12 metabolites were identified as 18α-hydroxyl euphorbiasteroid (**M1**), 4′-hydroxyl euphorbiasteroid (**M2**), 20-hydroxyl euphorbiasteroid (**M3**), 7′-hydroxyl euphorbiasteroid (**M4**), 2′-hydroxyl euphorbiasteroid (**M5**), 18α,2′-dihydroxyl euphorbiasteroid (**M8**), 5′,6′-dihydroxyl dihydroeuphorbiasteroid (**M12**), 3′,4′-dihydroxyl dihydroeuphorbiasteroid (**M13**), 18α-carboxyl euphorbiasteroid (**M16**), epoxylathyrol (**M24**), 15-deacetyl euphorbiasteroid (**M25**), and euphorbiasteroid 2′-*O-β-D*-glucopyranoside (**M29**).

### 3.5. Proposed Metabolic Pathways of Euphorbiasteroid In Vivo

Based on the metabolites identified in rats (plasma, urine, and feces), RLMs and fungus mycelium (Table 4), metabolic pathways of euphorbiasteroid can be proposed.

**Figure 9:**
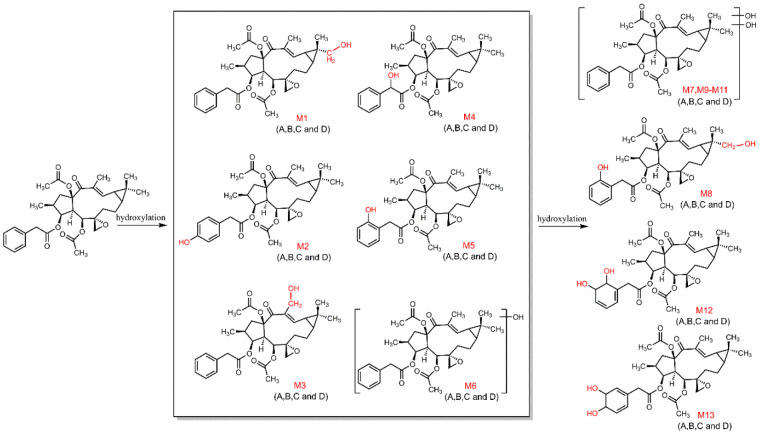
The proposed metabolic pathway of euphorbiasteroid in rats (I).

According to the above discoveries in the metabolism of euphorbiasteroid, the hydroxylation of euphorbiasteroid was the major metabolic pathway (Figure 9), including monohydroxylated euphorbiasteroid (**M1**–**M6**) and dihydroxylated euphorbiasteroid (**M7**–**M11**), which undergo further metabolism to form dihydrodiol (**M12**–**M13**).

**Figure 10:**
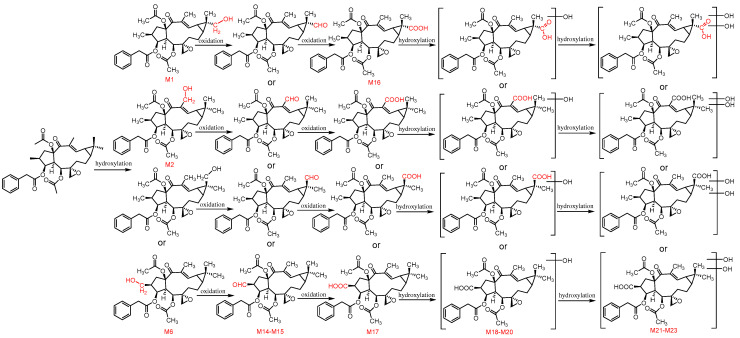
The proposed metabolic pathway of euphorbiasteroid in rats (Ⅱ).

The second pathway involves the methyl oxidation of euphorbiasteroid, from methyl to primary alcohol, then to aldehyde group (**M14**–**M15**), and finally to carboxyl group (**M16**–**M17**) (Figure 10). In addition, the metabolites (**M16**–**M17**) were further hydroxylated to form monohydroxylated products (**M18**–**M20**) and diehydroxylated products (**M21**–**M23**).

**Figure 11:**
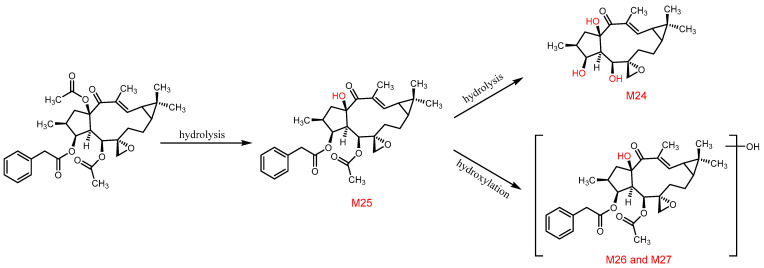
The proposed metabolic pathway of euphorbiasteroid in rats (Ⅲ).

The third pathway involves hydrolysis of ester groups to form epoxylathyrol (**M24**) and 15-deacetyl euphorbiasteroid (**M25**), followed by hydroxylation to produce metabolites **M26**–**M27**, as shown in Figure 11.

**Figure 12:**
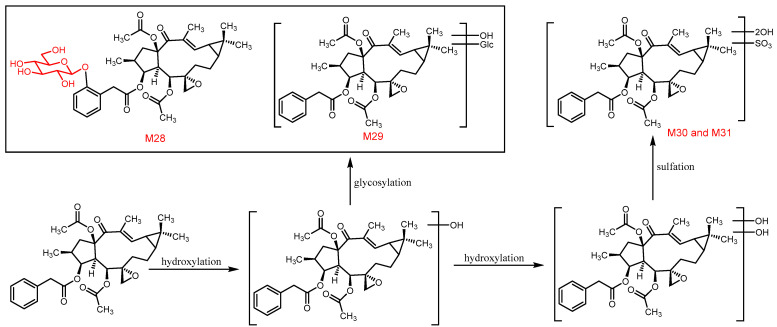
The proposed metabolic pathway of euphorbiasteroid in rats (Ⅳ).

Finally, phase II metabolism was the predominant pathway in rat feces samples, where several metabolites were formed by glycosylation and sulfonation (Figure 12). Overall, hydroxylation, oxidation, hydrolysis, sulfonation, and glycosylation are the main metabolic pathways of euphorbiasteroid in rats.

### 3.6. Cytotoxicity of Euphorbiasteroid and Its Metabolites

Euphorbiasteroid and its metabolites were tested for their cytotoxicity on human cell lines SH-SY5Y, LO2, AC-16, and HK-2 by the CCK-8 assay. The results (Table 5) indicated that euphorbiasteroid showed no cytotoxicity against four human cell lines with IC_50_ values of more than 50 μM. Among metabolites, the C-20 hydroxylated product **M3** (20-hydroxyl euphorbiasteroid) and two hydrolysis products **M24** (epoxylathyrol) and **M25** (15-deacetyl euphorbiasteroid) showed significant cytotoxicity against four human cell lines with IC_50_ values from 3.60 μM to 40.74 μM. Therefore, considering the high content of euphorbiasteroid in *Euphorbiae semen*, it was speculated that the metabolites from hydroxylation and hydrolysis might be the potential toxic constituents of *Euphorbiae semen*.

## 4. Conclusions

In the present study, euphorbiasteroid metabolites generated in vivo (rat plasma, urine and feces) and in vitro (RLMs and *C. elegans* bio-110930 model) were characterized through UPLC-Q/TOF-MS. According to the molecular ions and the MS/MS fragments, a total of 31 metabolites were identified, including 27 phase I and 4 phase II metabolites. Additionally, the structures of twelve metabolites were exactly confirmed by comparing their HPLC retention times and MS/MS fragments with those of the prepared reference standards, whose structures were exactly determined based on 1D and 2D NMR analysis. The twelve identified metabolites were 18α-hydroxyl euphorbiasteroid (**M1**), 4′-hydroxyl euphorbiasteroid (**M2**), 20-hydroxyl euphorbiasteroid (**M3**), 7′-hydroxyl euphorbiasteroid (**M4**), 2′-hydroxyl euphorbiasteroid (**M5**), 18α,2′-dihydroxyl euphorbiasteroid (**M8**), 5′,6′-dihydroxyl dihydroeuphorbiasteroid (**M12**), 3′,4′-dihydroxyl dihydroeuphorbiasteroid (**M13**), 18-carboxyl euphorbiasteroid (**M16**), epoxylathyrol (**M24**), 15-deacetyl euphorbiasteroid (**M25**), and euphorbiasteroid 2′-*O-β-D*-glucopyranoside (**M29**). These results showed that the majority of phase I metabolites were generated by hydroxylation and hydrolysis, followed by oxidation and hydroxylation. Glycosylation and sulfonation played significant roles in the formation of phase II metabolites. Moreover, RLMs and *C. elegans* bio-110930 could be suitable models to simulate and prepare phase I metabolites of euphorbiasteroid. Thus, an overall description of metabolites of euphorbiasteroid from rats, RLMs and *C. elegans* bio-110930 has been provided. Three metabolites **M3**, **M24**, and **M25** exhibited potent cytotoxicity against four human cell lines. Furthermore, our study provides valuable information for predicting in vivo human metabolites and important clues for further clarifying the mechanism of drug toxicity of euphorbiasteroid and its metabolites. The method can also be applied to the study of other herbal components, providing new ideas in the field of metabolic studies of traditional Chinese medicine.

## Figures and Tables

**Table 1:** The ^1^H NMR data for euphorbiasteroid and its transformation products (**1**–**6**).

Position	M0 ^a^	1 ^a^	2 ^a^	3 ^a^	4 ^a^	5 ^a^	6 ^b^
1a	3.32 (dd, 8.15, 13.68)	3.29 (dd, 8.29, 14.22)	3.32 (dd, 8.38, 14.26)	3.30 (dd, 8.67, 14,43)	3.19 (dd, 8.25, 14.37)	3.35 (dd, 8.25, 14.28)	3.20 (dd, 8.16, 12.94)
1b	1.35 (dd, 12.01, 13.68)	1.34 (dd, 12.42, 14.22)	1.37 (dd, 12.22, 14.26)	1.36 (dd, 12.80, 14.43)	1.04 (dd, 12.60, 14.37)	1.42 (dd, 12.26, 14.28)	1.41 (t, 12.94)
2	2.08 (m)	2.06 (overlap)	2.07 (m)	2.07 (m)	2.01 (m)	2.10 (overlap)	2.05 (overlap)
3	5.48 (brt., 3.32)	5.47 (brt., 3.15)	5.47 (brt., 3.05)	5.50 (s)	5.49 (brt., 2.84)	5.50 (brt, 3.03)	5.37 (brt, 2.95)
4	1.86 (dd, 3.32, 9.28)	1.85 (dd, 3.20, 9.35)	1.86 (dd, 3.05, 9.30)	1.92 (d, 9.66)	1.84 (dd, 2.84, 9.11)	1.88 (dd, 3.03, 9.17)	1.83 (dd, 2.95, 9.02)
5	6.24 (d, 9.28)	6.23 (d, 9.23)	6.23 (d, 9.30)	6.25 (d, 9.66)	6.28 (d, 9.11)	6.25 (d, 9.17)	6.29 (d, 9.02)
7a	1.87 (m)	2.14 (m)	2.12 (m)	2.14 (m)	2.12 (overlap)	2.10 (overlap)	2.10 (m)
7b	0.92 (m)	0.94 (t, 13.16)	0.93 (m)	0.98 (m)	0.94 (m)	0.93 (m)	0.97 (t, 13.93)
8a	2.10 (m)	2.06 (overlap)	2.07 (m)	2.12 (overlap)	2.09 (m)	2.07 (m)	2.00 (m)
8b	1.72 (m)	1.77 (m)	1.72 (m)	1.75 (m)	1.72 (m)	1.68 (m)	1.80 (m)
9	1.09 (m)	1.21 (m)	1.09 (ddd, 3.78, 8.08, 12.07)	1.21 (overlap)	1.09 (m)	1.09 (m)	1.33 (m)
11	1.48 (dd, 8.11, 11.34)	1.65 (dd, 8.39, 11.13)	1.48 (dd, 8.08, 11.35)	1.71 (m)	1.48 (dd, 8.20, 11.42)	1.47 (dd, 8.10, 11.39)	1.78 (overalp)
12	6.59 (d, 11.34)	6.59 (d, 11.23)	6.59 (dd, 1.05, 11.35)	6.71 (d, 12.41)	6.59 (dd, 1.05, 11.30)	6.59 (d, 11.39)	6.69 (d, 11.28)
16	0.66 (d, 6.63)	0.65 (d, 6.65)	0.67 (d, 6.66)	0.67 (d, 6.63)	0.33 (d, 6.70)	0.69 (d, 6.69)	0.76 (d, 6.72)
17a	2.48 (d, 3.37)	2.49 (d, 3.35)	2.49 (d, 3.40)	2.62 (s)	2.50 (d, 3.24)	2.50 (d, 3.24)	2.56 (d, 3.54)
17b	2.30 (brt., 3.37)	2.29 (brt., 3.35)	2.31 (brt., 3.40)	2.50 (s)	2.28 (brt., 3.24)	2.21 (brt., 3.24)	2.20 (t, 3.54)
18a	1.20 (s)	3.52 (d, 11.25)	1.20 (s)	1.21 (s)	1.21 (s)	1.20 (s)	3.48 (d, 11.25)
18b		3.44 (d, 11.25)					3.38 (d, 11.25)
19	1.21 (s)	1.27 (s)	1.21 (s)	1.22 (s)	1.22 (s)	1.21 (s)	1.27 (s)
20a	1.84 (s)	1.84 (s)	1.84 (brd, 0.81)	4.42 (d, 12.38)	1.83 (d, 1.04)	1.84 (s)	1.79 (d, 0.93)
20b				4.28 (d, 12.38)			
3-O-phenylacetyl							
2′	7.25 (overlap)	7.25 (overlap)	7.13 (d, 7.92)	7.27 (overlap)	7.37 (m)		
3′	7.30 (m)	7.29 (td, 1.21, 7.19)	6.76 (d, 7.92)	7.31 (t, 7.26)	7.32 (overlap)	6.90 (overlap)	6.86 (dd, 0.96, 7.35)
4′	7.27 (t, 7.76)	7.24 (m)		7.26 (overlap)	7.30 (overlap)	7.18 (t, 7.45)	7.08 (t, 7.35)
5′	7.30 (m)	7.29 (td, 1.21, 7.19)	6.76 (d, 7.92)	7.31 (t, 7.26)	7.32 (overlap)	6.88 (overlap)	6.78 (t, 7.35)
6′	7.25 (overlap)	7.25 (overlap)	7.13 (d, 7.92)	7.27 (overlap)	7.37 (m)	7.11 (dd, 1.45, 7.45)	7.18 (d, 7.35)
7′a	3.58 (2H, d, 3.21)	3.56 (2H, 2.71)	3.50 (2H, d, 3.57)	3.58 (2H, s)	5.10 (2H, s)	3.70 (d, 15.10)	3.65 (d, 15.64)
7′b						3.55 (d, 15.10)	3.55 (d, 15.64)
8′							
5-OAc							
C=O							
CH_3_	2.02 (s)	2.00 (s)	2.01 (s)	2.03 (s)	2.12 (s)	1.94 (s)	2.06 (s)
15-OAc							
C=O							
CH_3_	2.12 (s)	2.12 (s)	2.13 (s)	2.12 (s)	2.19 (s)	2.17 (s)	2.08 (s)

^a^ Measured at 500 MHz in CDCl_3_. ^b^ Measured at 500 MHz in CD_3_OD.

**Table 2:** The ^1^H NMR data for euphorbiasteroid and its transformation products (**7**–**12**).

Position	M0 ^a^	7 ^a^	8 ^a^	9 ^a^	10 ^a^	11 ^a^	12 ^a^
1a	3.32 (dd, 8.15, 13.68)	3.40 (dd, 8.21, 14.17)	3.39 (dd, 8.03, 13.95)	3.30 (dd, 8.24,14.25)	3.00 (dd, 9.58, 13.84)	3.15 (dd, 9.37, 14.18)	3.21 (dd, 7.65, 13.47)
1b	1.35 (dd, 12.01, 13.68)	1.52 (dd, 12.25, 14.17)	1.50 (dd, 12.69, 13.95)	1.36 (dd, 12.35, 14.25)	2.26 (dd, 6.08, 13.84)	0.91 (dd, 11.72, 14.18)	1.16 (overlap)
2	2.08 (m)	2.16 (m)	2.16 (m)	2.08 (m)	2.04 (m)	2.04 (m)	1.96 (m)
3	5.48 (brt., 3.32)	5.51 (brt., 3.08)	5.53 (brs.)	5.49 (brt., 2.80)	4.25 (brs)	5.46 (s)	5.37 (s)
4	1.86 (dd, 3.32, 9.28)	1.89 (dd, 3.08, 9.06)	1.89 (m)	1.87 (overlap)	1.28 (brs)	1.76 (dd, 2.84, 9.91)	1.79 (overlap)
5	6.24 (d, 9.28)	6.22 (d, 9.06)	6.24 (d, 8.82)	6.22 (d, 9.10)	4.39 (brs)	5.92 (d, 9.91)	6.21 (d, 8.90)
7a	1.87 (m)	2.08 (m)	2.08 (m)	2.17 (m)	2.04 (m)	2.12 (dd, 6.42, 13.12)	2.08 (overlap)
7b	0.92 (m)	0.92 (t, 13.69)	0.93 (t, 12.07)	1.01 (t, 13.36)	1.05 (m)	0.87 (m)	0.90 (m)
8a	2.10 (m)	2.04 (m)	2.05 (m)	2.11 (m)	1.77 (m)	2.08 (overlap)	2.08 (overlap)
8b	1.72 (m)	1.73 (m)	1.71 (m)	1.77 (m)	1.67 (dd, 10.35,13.80)	1.69 (m)	1.69 (m)
9	1.09 (m)	1.08 (m)	1.08 (m)	1.90 (overlap)	1.16 (m)	1.09 (m)	1.07 (brt., 8.28)
11	1.48 (dd, 8.11, 11.34)	1.48 (dd,8.07, 11.30)	1.48 (dd,8.37, 11.17)	2.44 (dd, 9.04, 11.20)	1.42 (dd, 8.48, 11.02)	1.48 (dd, 8.21, 11.60)	1.46 (dd, 8.28, 11.11)
12	6.59 (d, 11.34)	6.60 (d, 11.03)	6.60 (d, 11.17)	6.47 (d, 11.20)	6.68 (d, 8.48)	7.32 (overlap)	6.56 (d, 11.11)
16	0.66 (d, 6.63)	0.87 (d, 6.72)	0.85 (d, 6.39)	0.67 (d, 6.58)	1.11 (d, 6.84)	0.81 (d, 6.69)	0.42 (d, 6.46)
17a	2.48 (d, 3.37)	2.48 (d, 3.46)	2.49 (d, 3.33)	2.53 (d, 3.11)	2.64 (d, 3.79)	2.41 (d, 2.24)	2.46 (s)
17b	2.30 (brt., 3.37)	2.32 (brt, 3.46)	2.32 (brs)	2.29 (brt., 3.11)	2.59 (d, 3.79)	2.27 (s)	2.26 (s)
18	1.20 (s)	1.19 (s)	1.19 (s)		1.14 (s)	1.21 (s)	1.19 (s)
19	1.21 (s)	1.19 (s)	1.19 (s)	1.44 (s)	1.15 (s)	1.25 (s)	1.20 (s)
20	1.84 (s)	1.85 (s)	1.85 (s)	1.88 (s)	1.89 (s)	1.78 (s)	1.81 (s)
3-O-phenylacetyl							
2′	7.25 (overlap)	5.54 (brs)	5.66 (s)	7.27 (overlap)		7.32 (overlap)	
3′	7.30 (m)	7.54 (d, 10.30)	3.72 (brs)	7.31 (td, 0.83, 7.12)		7.36 (t, 7.35)	7.01 (d, 7.36)
4′	7.27 (t, 7.76)	6.07 (dt, 1.81, 10.30)	4.25 (brd., 5.56)	7.26 (m)		7.28 (t, 7.21)	7.20 (t, 7.36)
5′	7.30 (m)	4.26 (d, 7.49)	6.07 (m)	7.31 (td, 0.83, 7.12)		7.36 (t, 7.35)	6.92 (t, 7.36)
6′	7.25 (overlap)	3.76 (m)	6.15 (d, 10.14)	7.27 (overlap)		7.32 (overlap)	7.12 (d, 7.36)
7′a	3.58 (2H, d, 3.21)	2.67 (m)	3.69 (m)	3.58 (2H, s)		3.67 (d, 16.65)	3.81 (d, 13.39)
7′b		2.54 (m)	2.55 (m)	7.27 (overlap)		3.64 (d, 16.65)	3.35 (d, 13.39)
8′							
5-OAc							
C=O							
CH_3_	2.02 (s)	2.00 (s)	2.00 (s)	2.02 (s)		2.08 (s)	2.04 (s)
15-OAc							
C=O							
CH_3_	2.12 (s)	2.13 (s)	2.13 (s)	2.13 (s)			2.17 (s)
Glc							
1′′							4.81 (d, 6.77)
2′′							3.69 (overlap)
3′′							3.49 (m)
4′′							3.69 (overlap)
5′′							3.65 (m)
6′′a							3.89 (d, 13.27)
6′′b							3.85 (d, 13.27)

^a^ Measured at 500 MHz in CDCl_3_.

**Table 3:** The ^13^C NMR data for euphorbiasteroid and its transformation products (**1**–**12**).

Position	M0 ^a^	1 ^a^	2 ^a^	3 ^a^	4 ^a^	5 ^a^	6 ^b^	7 ^a^	8 ^a^	9 ^a^	10 ^a^	11 ^a^	12 ^a^
1	47.9(d)	47.8 (t)	47.9 (t)	47.3 (t)	47.5 (t)	47.7(t)	48.3 (t)	47.9 (t)	47.9 (t)	47.9 (t)	48.2 (t)	49.9 (t)	47.7 (t)
2	37.7 (s)	37.7 (d)	37.8 (d)	37.8 (d)	37.8 (d)	37.8 (d)	38.5 (d)	37.8 (d)	37.9 (d)	37.7 (d)	37.6 (d)	37.2 (d)	37.6 (d)
3	80.6(d)	80.6 (d)	80.6 (d)	80.6 (d)	82.6 (d)	81.5 (d)	80.9 (d)	79.8 (d)	79.8 (d)	80.6 (d)	78.9 (d)	82.8 (d)	81.0 (d)
4	49.9 (s)	49.9 (d)	49.9 (d)	49.9 (d)	49.7 (d)	49.7 (d)	50.6 (d)	49.9 (d)	49.7 (d)	50.0 (d)	53.4 (d)	51.6 (d)	49.7 (d)
5	65.2(d)	65.1 (d)	65.2 (d)	65.1 (d)	65.1 (d)	65.3 (d)	65.9 (d)	65.2 (d)	65.2 (d)	65.0 (d)	66.5 (d)	66.0 (d)	65.2 (d)
6	58.9 (s)	58.9 (s)	59.0 (s)	58.6 (s)	58.9 (s)	58.9 (s)	59.4 (s)	59.0 (s)	59.0 (s)	58.7 (s)	60.8 (s)	58.9 (s)	58.9 (s)
7	33.5(d)	33.4 (t)	33.5 (t)	33.4 (t)	33.4 (t)	33.5 (t)	34.1 (t)	33.5 (t)	33.4 (t)	32.9 (t)	32.1 (t)	33.9 (t)	33.4 (t)
8	20.0(d)	19.7 (t)	20.1 (t)	20.0 (t)	20.1 (t)	20.1 (t)	20.6 (t)	20.0 (t)	20.0 (t)	19.3 (t)	19.7 (t)	20.0 (t)	20.0 (t)
9	34.8 (s)	30.2 (d)	34.8 (d)	35.8 (d)	34.8 (d)	34.8 (d)	31.1 (d)	34.8 (d)	34.8 (d)	32.9 (d)	34.7 (d)	35.7 (d)	34.8 (d)
10	25.6 (s)	31.0 (s)	25.7 (s)	26.9 (s)	25.7 (s)	25.7 (s)	32.1 (s)	25.6 (s)	25.6 (s)	29.9 (s)	25.1 (s)	26.4 (s)	25.6 (s)
11	29.0 (d)	25.2 (d)	29.0 (d)	29.0 (d)	29.1 (d)	29.0 (d)	26.2 (d)	29.1 (d)	29.1 (d)	30.1 (d)	28.7 (d)	29.3 (d)	29.0 (d)
12	143.7 (t)	142.1(d)	143.8(d)	147.6(d)	143.8(d)	143.7(d)	144.1(d)	143.7(d)	143.7(d)	137.9(d)	144.6(d)	150.8(d)	143.7(d)
13	136.0(d)	136.7 (s)	136.0 (s)	138.4 (s)	136.0 (s)	136.0 (s)	136.7 (s)	136.0 (s)	135.9 (s)	139.0 (s)	136.2 (s)	135.1 (s)	135.9 (s)
14	196.9(d)	196.9 (s)	196.9(s)	198.4 (s)	196.6(s)	196.7(s)	197.3 (s)	196.9 (s)	196.8 (s)	197.2 (s)	202.8 (s)	199.3 (s)	197.3 (s)
15	91.7 (s)	91.7 (s)	91.8 (s)	91.5 (s)	91.5 (s)	91.6 (s)	92.2 (s)	91.8 (s)	91.7 (s)	91.7 (s)	88.4 (s)	88.7 (s)	91.6 (s)
16	13.5(q)	13.5 (q)	13.6 (q)	13.5 (q)	12.8 (q)	13.4 (q)	13.8 (q)	14.0 (q)	14.0 (q)	13.5 (q)	13.9 (q)	14.2 (q)	13.1 (q)
17	55.4 (t)	55.3 (t)	55.4 (t)	56.2 (t)	55.3 (t)	55.3 (t)	55.3 (t)	55.3 (t)	55.3 (t)	55.2 (t)	53.4 (t)	55.4 (t)	55.3 (t)
18	28.9(q)	71.2 (t))	28.9 (q)	28.8 (q)	28.9 (q)	28.9 (q)	70.9 (t)	16.7 (q)	16.7 (q)	179.8 (s)	28.7 (q)	29.0 (q)	28.9 (q)
19	16.8(q)	12.5 (q)	16.8 (q)	16.7 (q)	16.8 (q)	16.8 (q)	12.9 (q)	28.9 (q)	28.9 (q)	10.4 (q)	15.8 (q)	16.3 (q)	16.7 (q)
20	12.3 (t)	12.4 (q)	12.4 (q)	58.1 (t)	12.3 (q)	12.4 (q)	12.5 (q)	12.4 (q)	12.4 (q)	12.5 (q)	13.0 (q)	12.2 (t)	12.3 (q)
3-O-phenylacetyl													
1′	133.8 (s)	133.7 (s)	125.8 (s)	133.7 (s)	138.4 (s)	120.3 (s)	121.1 (s)	150.2 (s)	151.0 (s)	133.6 (s)		133.8 (s)	123.1 (s)
2′	129.4(d)	129.4(d)	130.6(d)	129.4(d)	126.5(d)	154.7 (s)	156.3 (s)	115.5(d)	117.6(d)	129.4 (s)		129.6(d)	155.3 (s)
3′	128.5(d)	128.5(d)	115.4(d)	128.5(d)	128.4(d)	117.4(d)	115.8(d)	125.5(d)	72.3 (d)	128.5(d)		128.9(d)	114.5(d)
4′	127.2(d)	127.2(d)	154.9 (s)	127.3(d)	128.7(d)	129.2(d)	129.1(d)	138.1(d)	72.2 (d)	127.3(d)		127.6(d)	129.2(d)
5′	128.5(d)	128.5(d)	115.4(d)	128.5(d)	128.4(d)	120.9(d)	120.1(d)	73.5 (d)	137.1(d)	128.5(d)		128.9(d)	122.5(d)
6′	129.4(d)	129.4(d)	130.6(d)	129.4(d)	126.5(d)	131.1(d)	132.3(d)	72.9 (d)	130.7(d)	129.4(d)		129.4(d)	131.2(d)
7′	41.53 (t)	41.5 (t)	40.6 (t)	41.5 (t)	72.8 (d)	37.3 (t)	36.4 (t)	38.5 (t)	32.4 (t)	41.5 (t)		41.5 (t)	36.4 (t)
8′	170.9(s)	170.9 (s)	171.3 (s)	170.9 (s)	173.7 (s)	172.1 (s)	171.7 (s)	165.8 (s)	165.8 (s)	170.9 (s)		169.9 (s)	172.0 (s)
5-OAc													
C=O	170.8(s)	170.7 (s)	170.8 (s)	170.7 (s)	170.8 (s)	171.2 (s)	170.9 (s)	170.6 (s)	170.7 (s)	170.8 (s)		171.0 (s)	171.1 (s)
CH_3_	21.0(q)	21.0 (q)	21.0 (q)	21.0 (q)	20.9 (q)	20.8 (q)	21.1 (q)	20.9 (q)	20.8 (q)	21.0 (q)		21.1 (q)	20.9 (q)
15-OAc													
C=O	169.6(s)	169.6 (s)	169.7 (s)	169.6 (s)	169.4 (s)	169.8 (s)	170.2 (s)	169.7 (s)	169.8 (s)	169.6 (s)			169.6 (s)
CH_3_	21.9 (s)	21.9 (q)	21.9 (q)	21.8 (q)	21.8 (q)	21.9 (q)	22.0 (q)	21.9 (q)	21.9 (q)	21.8 (q)			21.9 (q)
Glc													
1′′													101.2(d)
2′′													73.5 (d)
3′′													75.9 (d)
4′′													69.6 (d)
5′′													75.9 (d)
6′′													61.8 (t)

^a^ Measured at 125 MHz in CDCl_3_. ^b^ Measured at 125 MHz in CD_3_OD.

**Table 4:** Mass spectrum characteristics of metabolites of euphorbiasteroid detected in vivo and in vitro.

No.	Component Name	RT (min)	Formula [M + H]^+^	Observed *m*/*z*	Error (ppm)	MS ^n^	Distribution				
							Rat			RLMs	Fungi
							Plasma	Urine	Faeces		
**M1**	M + O	8.49	C_32_H_41_O_9_	569.2746	0.18	569.2746, 509.2533, 449.2336, 433.2231, 373.2012, 355.1910, 313.1801, 295.1697, 277.1595, 267.1741, 249.1643	√	√	√	√	√
**M2**	M + O	9.79	C_32_H_41_O_9_	569.2748	0.53	569.2748, 509.2543, 449.2332, 417.2275, 375.2172, 357.2069, 315.1955, 297.1854, 279.1748, 269.1904, 251.1803	-	√	√	√	√
**M3**	M + O	9.99	C_32_H_41_O_9_	569.2747	0.35	569.2747, 551.2642, 509.2538, 491.2438,449.2336, 431.2226, 373.2020, 355.1910, 313.1805, 295.1699, 277.1592, 267.1745, 249.1645	√	√	√	√	√
**M4**	M + O	10.34	C_32_H_41_O_9_	569.2756	1.93	569.2756, 509.2549, 449.2331, 417.2286, 375.2184, 357.2071, 315.1959, 297.1858, 279.1751, 269.1907, 251.1805	√	√	√	√	√
**M5**	M + O	10.66	C_32_H_41_O_9_	569.2754	1.58	569.2754, 509.2547, 449.2329, 417.2288, 375.2181, 357.2071, 315.1959, 297.1865, 279.1751, 269.1912	√	-	√	√	√
**M6**	M + O	11.38	C_32_H_41_O_9_	569.2747	0.35	569.2747, 509.2542, 449.2339, 431.2230, 373.2020, 355.1910, 313.1806, 295.1699, 277.1592, 267.1745	√	-	√	√	-
**M7**	M + 2O	6.06	C_32_H_41_O_10_	585.2688	−1.03	585.2688, 525.2480, 507.2377, 433.2221, 373.1995, 331.1906, 313.1796, 295.1701, 285.1841, 277.1594	√	√	-	√	-
**M8**	M + 2O	6.36	C_32_H_41_O_10_	585.2704	1.71	585.2704, 525.2490, 507.2382, 465.2271, 433.2222, 391.2116, 373.2011, 355.1904, 313.1805, 295.1698, 285.1847, 267.1743, 255.1382	√	√	√	√	√
**M9**	M + 2O	6.59	C_32_H_41_O_10_	585.2689	−0.85	585.2689, 525.2485, 507.2382, 465.2272, 447.2169, 433.2221, 391.2114, 373.2005, 355.1900, 313.1806, 295.1689, 285.1847, 277.1591, 267.1749	√	√	√	√	√
**M10**	M + 2O	6.92	C_32_H_41_O_10_	585.2692	−0.34	585.2692, 525.2480, 507.2377, 465.2278, 391.2118, 373.2010, 355.1907, 313.1805, 295.1695, 267.1747	√	-	√	√	-
**M11**	M + 2O	7.12	C_32_H_41_O_10_	585.2714	3.42	585.2714, 525.2496, 465.2284, 373.1997, 355.1906, 313.1802, 295.1706	√	-	-	√	-
**M12**	M + 2O + 2H	7.35	C_32_H_43_O_10_	587.2858	0.17	587.2858, 527.2645, 467.2433, 357.2069, 315.1961, 297.1860, 279.1752, 269.1905, 251.1796	√	-	√	√	√
**M13**	M + 2O + 2H	7.49	C_32_H_43_O_10_	587.2867	2.72	587.2867, 527.2661, 509.2549, 467.2442, 449.2337, 417.2285, 357.2074, 315.1963, 297.1858, 279.1757, 269.1906	√	-	√	√	√
**M14**	M + O-2H	8.80	C_32_H_39_O_9_	567.2598	1.59	567.2598, 507.2377, 447.2166, 371.1853, 329.1760, 311.1644, 293.1538, 283.1700, 265.1597, 255.1747	√	√	√	-	√
**M15**	M + O-2H	8.95	C_32_H_39_O_9_	567.2598	1.59	567.2598, 507.2377, 447.2166, 371.1852, 329.1761, 311.1654, 293.1547, 283.1700, 265.1597	√	√	√	-	-
**M16**	M + 2O-2H	8.71	C_32_H_39_O_10_	583.2530	−1.37	583.2530, 523.2330, 463.2126, 447.2032, 387.1815, 345.1707, 327.1595, 309.1498, 299.1645, 281.1541, 263.1438, 253.1591	√	√	√	√	√
**M17**	M + 2O-2H	9.49	C_32_H_39_O_10_	583.2548	1.71	583.2548, 523.2332, 463.2132, 387.1814, 345.1710, 327.1600, 309.1490, 299.1647, 281.1540, 263.1449, 253.1596	√	-	√	√	√
**M18**	M + 3O-2H	5.96	C_32_H_39_O_11_	599.2497	1.67	599.2497, 539.2279, 387.1814, 345.1704, 327.1600, 309.1490, 299.1645, 281.1540, 263.1425, 253.1594	√	√	√	-	-
**M19**	M + 3O-2H	6.37	C_32_H_39_O_11_	599.2505	3.00	599.2505, 539.2284, 479.2076, 447.2024, 405.1918, 387.1812, 345.1705, 327.1601, 309.1494, 299.1647, 281.1541, 263.1436, 253.1585	√	√	√	√	-
**M20**	M + 3O-2H	6.75	C_32_H_39_O_11_	599.2500	2.17	599.2500, 539.2286, 387.1811, 345.1711, 327.1602, 309.1496, 299.1648, 281.1547, 263.1438, 253.1591	√	√	√	-	-
**M21**	M + 4O-2H	4.61	C_32_H_39_O_12_	615.2451	2.44	615.2451, 597.2335, 555.2226, 537.2123, 477.1913, 385.1654, 343.1542, 325.1440, 307.1334, 297.1497, 279.1386, 261.1280, 251.1426	√	√	√	-	-
**M22**	M + 4O-2H	5.04	C_32_H_39_O_12_	615.2460	3.90	615.2460, 555.2236, 537.2133, 477.1919, 343.1549, 325.1448, 297.1494, 279.1389, 261.1285, 251.1437	√	√	√	-	-
**M23**	M + 4O-2H	5.77	C_32_H_39_O_12_	615.2453	2.76	615.2453, 555.2231, 537.2125, 477.1916, 343.1549, 325.1440, 307.1335, 297.1493, 279.1390, 261.1286, 251.1439	√	√	√	-	-
**M24**	M-2CH_2_CO-C_8_H_6_O	5.84	C_20_H_31_O_5_	351.2166	0.00	351.2166, 333.2059, 315.1951, 297.1849, 279.1746, 269.1898, 251.1802	√	√	√	√	-
**M25**	M-C_2_H_2_O	12.49	C_30_H_39_O_7_	511.2694	0.78	511.2694, 451.2482, 433.2373, 375.2171, 357.2068, 315.1959, 297.1858, 279.1750, 269.1902, 251.1431	√	√	√	√	-
**M26**	M-C_2_H_2_O + O	7.66	C_30_H_39_O_8_	527.2661	4.17	527.2661, 509.2540, 467.2440, 449.2337, 391.2120, 373.2017, 331.1910, 313.1803, 295.1696	√	√	√	-	-
**M27**	M-C_2_H_2_O + O	9.14	C_30_H_39_O_8_	527.2647	1.52	527.2647, 509.2553, 467.2435, 449.2330, 391.2122, 373.2020, 331.1909, 313.1804, 295.1706	√	√	√	-	√
**M28**	M + O+ C_6_H_10_O_5_	5.23	C_38_H_51_O_14_	731.3280	0.96	731.3280, 569.2768, 509.2549, 449.2335, 357.2074, 315.1971, 297.1868, 279.1754, 269.1911	-	-	√	-	-
**M29**	M + O+ C_6_H_10_O_5_	6.97	C_38_H_51_O_14_	731.3286	1.78	731.3286, 671.3065, 569.2745, 509.2544, 449.2331, 375.2173, 357.2073, 315.1963, 297.1855, 279.1752, 269.1902	√	-	√	-	√
**M30**	M + 2O+ SO_3_	2.87	C_32_H_41_O_13_S	665.2271	1.35	665.2271, 647.2167, 605.2055, 587.1955, 545.1848, 393.1376, 313.1788, 295.1683, 277.1589, 267.1744	-	-	√	-	-
**M31**	M + 2O+ SO_3_	3.19	C_32_H_41_O_13_S	665.2267	0.75	665.2267, 605.2061, 545.1843, 393.1371, 313.1805, 295.1691, 277.1580	-	-	√	-	-

**Table 5:** The cytotoxicities of euphorbiasteroid and its metabolites on four strains of human cells.

Compound	**IC_50_ (μM)**			
	SH-SY5Y	LO2	AC-16	HK-2
**M0**	54.95 ± 1.20	>100	97.72 ± 2.13	>100
**M3**	39.63 ± 0.43	37.41 ± 0.41	17.86 ± 0.19	22.65 ± 0.25
**M24**	40.74 ± 0.44	38.9 ± 0.42	21.73 ± 0.24	26.49 ± 0.29
**M25**	33.27 ± 0.36	30.13 ± 0.33	3.60 ± 0.04	16.11 ± 0.18

## Data Availability

The data presented in this study are available in Supplementary Materials.

## Supplementary Materials

The following supporting information can be downloaded at: https://www.mdpi.com/article/10.3390/metabo12090830/s1. Figure S1. HR-ESI-MS spectrum of compound **1**; Figure S2. OR Valve of compound **1** in MeOH; Figure S3. UV spectrum of compound **1** in MeOH; Figure S4. CD spectrum of compound **1** in MeOH; Figure S5. ^1^H NMR spectrum of compound **1** in CDCl_3_; Figure S6. ^13^C NMR and DEPT spectrum of compound **1** in CDCl_3_; Figure S7. ^1^H-^1^H COSY spectrum of compound **1** in CDCl_3_; Figure S8. HMBC spectrum of compound **1** in CDCl_3_; Figure S9. HSQC spectrum of compound **1** in CDCl_3_; Figure S10. NOESY spectrum of compound **1** in CDCl_3_; Figure S11. HR-ESI-MS spectrum of compound **2**; Figure S12. OR Valve of compound **2** in MeOH; Figure S13. UV spectrum of compound **2** in MeOH; Figure S14. CD spectrum of compound **2** in MeOH; Figure S15. ^1^H NMR spectrum of compound **2** in CDCl_3_; Figure S16. ^13^C NMR and DEPT spectrum of compound **2** in CDCl_3_; Figure S17. ^1^H-^1^H COSY spectrum of compound **2** in CDCl_3_; Figure S18. HMBC spectrum of compound **2** in CDCl_3_; Figure S19. HSQC spectrum of compound **2** in CDCl_3_; Figure S20. NOESY spectrum of compound **2** in CDCl_3_; Figure S21. HR-ESI-MS spectrum of compound **3**; Figure S22. OR valve of compound **3** in MeOH; Figure S23. UV spectrum of compound **3** in MeOH; Figure S24. CD spectrum of compound **3** in MeOH; Figure S25. ^1^H NMR spectrum of compound **3** in CDCl_3_; Figure S26. ^13^C NMR and DEPT spectrum of compound **3** in CDCl_3_; Figure S27. ^1^H-^1^H COSY spectrum of compound **3** in CDCl_3_; Figure S28. HMBC spectrum of compound **3** in CDCl_3_; Figure S29. HSQC spectrum of compound **3** in CDCl_3_; Figure S30. NOESY spectrum of compound **3** in CDCl_3_; Figure S31. HR-ESI-MS spectrum of compound **4**; Figure S32. OR valve of compound **4** in MeOH; Figure S33. UV spectrum of compound **4** in MeOH; Figure S34. CD spectrum of compound **4** in MeOH; Figure S35. ^1^H NMR spectrum of compound **4** in CDCl_3_; Figure S36. ^13^C NMR and DEPT spectrum of compound **4** in CDCl_3_; Figure S37. ^1^H-^1^H COSY spectrum of compound **4** in CDCl_3_; Figure S38. HMBC spectrum of compound **4** in CDCl_3_; Figure S39. HSQC spectrum of compound **4** in CDCl_3_; Figure S40. NOESY spectrum of compound **4** in CDCl_3_; Figure S41. HR-ESI-MS spectrum of compound **5**; Figure S42. OR valve of compound **5** in MeOH; Figure S43. UV spectrum of compound **5** in MeOH; Figure S44. CD spectrum of compound **5** in MeOH; Figure S45. ^1^H NMR spectrum of compound **5** in CDCl_3_; Figure S46. ^13^C NMR and DEPT spectrum of compound **5** in CDCl_3_; Figure S47. ^1^H-^1^H COSY spectrum of compound **5** in CDCl_3_; Figure S48. HMBC spectrum of compound **5** in CDCl_3_; Figure S49. HSQC spectrum of compound **5** in CDCl_3_; Figure S50. NOESY spectrum of compound **5** in CDCl_3_; Figure S51. HR-ESI-MS spectrum of compound **6**; Figure S52. OR valve of compound **6** in MeOH; Figure S53. UV spectrum of compound **6** in MeOH; Figure S54. CD spectrum of compound **6** in MeOH; Figure S55. ^1^H NMR spectrum of compound **6** in C_3_D_6_O; Figure S56. ^13^C NMR and DEPT spectrum of compound **6** in C_3_D_6_O; Figure S57. ^1^H - ^1^H COSY spectrum of compound **6** in C_3_D_6_O; Figure S58. HMBC spectrum of compound **6** in C_3_D_6_O; Figure S59. HSQC spectrum of compound **6** in C_3_D_6_O; Figure S60. NOESY spectrum of compound **6** in C_3_D_6_O; Figure S61. HR-ESI-MS spectrum of compound **7**; Figure S62. OR valve of compound **7** in MeOH; Figure S63. UV spectrum of compound **7** in MeOH; Figure S64. CD spectrum of compound **7** in MeOH; Figure S65. ^1^H NMR spectrum of compound **7** in CDCl_3_; Figure S66. ^13^C NMR and DEPT spectrum of compound **7** in CDCl_3_; Figure S67. ^1^H-^1^H COSY spectrum of compound **7** in CDCl_3_; Figure S68. HMBC spectrum of compound **7** in CDCl_3_; Figure S69. HSQC spectrum of compound **7** in CDCl_3_; Figure S70. NOESY spectrum of compound **7** in CDCl_3_; Figure S71. HR-ESI-MS spectrum of compound **8**; Figure S72. OR Valve of compound **8** in MeOH; Figure S73. UV spectrum of compound **8** in MeOH; Figure S74. CD spectrum of compound **8** in MeOH; Figure S75. ^1^H NMR spectrum of compound **8** in CDCl_3_; Figure S76. ^13^C NMR and DEPT spectrum of compound **8** in CDCl_3_; Figure S77. ^1^H-^1^H COSY spectrum of compound **8** in CDCl_3_; Figure S78. HMBC spectrum of compound **8** in CDCl_3_; Figure S79. HSQC spectrum of compound **8** in CDCl_3_; Figure S80. NOESY spectrum of compound **8** in CDCl_3_; Figure S81. HR-ESI-MS spectrum of compound **9**; Figure S82. OR Valve of compound **9** in MeOH; Figure S83. UV spectrum of compound **9** in MeOH; Figure S84. CD spectrum of compound **9** in MeOH; Figure S85. ^1^H NMR spectrum of compound **9** in CDCl_3_; Figure S86. ^13^C NMR and DEPT spectrum of compound **9** in CDCl_3_; Figure S87. ^1^H - ^1^H COSY spectrum of compound **9** in CDCl_3_; Figure S88. HMBC spectrum of compound **9** in CDCl_3_; Figure S89. HSQC spectrum of compound **9** in CDCl_3_; Figure S90. NOESY spectrum of compound **9** in CDCl_3_; Figure S91. HR-ESI-MS spectrum of compound **10**; Figure S92. ^1^H NMR spectrum of compound **10** in CDCl_3_; Figure S93. ^13^C NMR and DEPT spectrum of compound **10** in CDCl_3_; Figure S94. HR-ESI-MS spectrum of compound **11**; Figure S95. OR valve of compound **11** in MeOH; Figure S96. UV spectrum of compound **11** in MeOH; Figure S97. CD spectrum of compound **11** in MeOH; Figure S98. ^1^H NMR spectrum of compound **11** in CDCl_3_; Figure S99. ^13^C NMR and DEPT spectrum of compound **11** in CDCl_3_; Figure S100. ^1^H-^1^H COSY spectrum of compound **11** in CDCl_3_; Figure S101. HMBC spectrum of compound **11** in CDCl_3_; Figure S102. HSQC spectrum of compound **11** in CDCl_3_; Figure S103. NOESY spectrum of compound **11** in CDCl_3_; Figure S104. HR-ESI-MS spectrum of compound **12**; Figure S105. OR valve of compound **12** in MeOH; Figure S106. UV spectrum of compound **12** in MeOH; Figure S107. CD spectrum of compound **12** in MeOH; Figure S108. ^1^H NMR spectrum of compound **12** in CDCl_3_; Figure S109. ^13^C NMR and DEPT spectrum of compound **12** in CDCl_3_; Figure S110. ^1^H - ^1^H COSY spectrum of compound **12** in CDCl_3_; Figure S111. HMBC spectrum of compound **12** in CDCl_3_; Figure S112. HSQC spectrum of compound **12** in CDCl_3_; Figure S113. NOESY spectrum of compound **12** in CDCl_3_; Table S1: Effects of metabolites on proliferation of four strains of human cells (Inhibition rate %).

## Abbreviations

*C. elegans*, *Cunninghamella elegans*; HPLC-MS, high performance liquid chromatography-mass spectrometry; LC, liquid chromatography; NMR, Nuclear magnetic resonance; RLMs, rat liver microsomes; UPLC-Q/TOF-MS, ultra-performance liquid chromatography coupled with quadrupole time-of-flight mass spectrometry.
