# Supplementary material for: Comprehensive Metabolic Profiling of Euphorbiasteroid in Rats by Integrating UPLC-Q/TOF-MS and NMR as Well as Microbial Biotransformation
Source: Metabolites. 2022 Sep 2;12(9):830. doi: 10.3390/metabo12090830 (PMC9504842; doi:10.3390/metabo12090830)
Supplement: Supplementary file 1 [file metabolites-12-00830-s001.zip › metabolites-1883572-supplementary.pdf]

# Supplementary Materials

## Comprehensive metabolic profiling of euphorbiasteroid in rats by integrating UPLC-Q/TOF-MS and NMR as well as microbial biotransformation

**Sijia Xiao [1,†], Xike Xu [1, †], Xintong Wei [2, †], Jiayun Xin [1, 2], Shanshan Li [3], Yanhui Lv [1, 2], Wei Chen [1], Wenlin Yuan [1], Bin Xie [1], Xianpeng Zu [1,*], Yunheng Shen [1,*]**

[1] School of Pharmacy, Naval Medical University, Shanghai 200433, China

[2] School of Pharmacy, Shandong University of Traditional Chinese Medicine, Jinan 250355, China

[3] School of Pharmaceutical Sciences and Yunnan Key Laboratory of Pharmacology for Natural Products, Kunming Medical University, Kunming 650500, Yunnan, China

[*] Correspondence: zuxianpeng@163.com (X.Z.); shenyunheng@hotmail.com (Y.S.)

[†] These authors contributed equally to this work.

# Contents

Figure S1. HR-ESI-MS spectrum of compound **1**

Figure S2. OR Valve of compound **1** in MeOH

Figure S3. UV spectrum of compound **1** in MeOH

Figure S4. CD spectrum of compound **1** in MeOH

Figure S5. $^{1}$H NMR spectrum of compound **1** in $CDCl_3$

Figure S6. $^{13}$C NMR and DEPT spectrum of compound **1** in $CDCl_3$

Figure S7. $^{1}$H-$^{1}$H COSY spectrum of compound **1** in $CDCl_3$

Figure S8. HMBC spectrum of compound **1** in $CDCl_3$

Figure S9. HSQC spectrum of compound **1** in $CDCl_3$

Figure S10. NOESY spectrum of compound **1** in $CDCl_3$

Figure S11. HR-ESI-MS spectrum of compound **2**

Figure S12. OR Valve of compound **2** in MeOH

Figure S13. UV spectrum of compound **2** in MeOH

Figure S14. CD spectrum of compound **2** in MeOH

Figure S15. $^{1}$H NMR spectrum of compound **2** in $CDCl_3$

Figure S16. $^{13}$C NMR and DEPT spectrum of compound **2** in $CDCl_3$

Figure S17. $^{1}$H-$^{1}$H COSY spectrum of compound **2** in $CDCl_3$

Figure S18. HMBC spectrum of compound **2** in $CDCl_3$

Figure S19. HSQC spectrum of compound **2** in $CDCl_3$

Figure S20. NOESY spectrum of compound **2** in $CDCl_3$

Figure S21. HR-ESI-MS spectrum of compound **3**

Figure S22. OR valve of compound **3** in MeOH

Figure S23. UV spectrum of compound **3** in MeOH

Figure S24. CD spectrum of compound **3** in MeOH

Figure S25. $^{1}$H NMR spectrum of compound **3** in $CDCl_3$

Figure S26. $^{13}$C NMR and DEPT spectrum of compound **3** in $CDCl_3$

Figure S27. $^{1}$H-$^{1}$H COSY spectrum of compound **3** in $CDCl_3$

Figure S28. HMBC spectrum of compound **3** in $CDCl_3$

Figure S29. HSQC spectrum of compound **3** in $CDCl_3$

Figure S30. NOESY spectrum of compound **3** in $CDCl_3$

Figure S31. HR-ESI-MS spectrum of compound **4**

Figure S32. OR valve of compound **4** in MeOH

Figure S33. UV spectrum of compound **4** in MeOH

Figure S34. CD spectrum of compound **4** in MeOH

Figure S35. $^1H$ NMR spectrum of compound **4** in $CDCl_3$

Figure S36. $^{13}C$ NMR and DEPT spectrum of compound **4** in $CDCl_3$

Figure S37. $^1H$-$^1H$ COSY spectrum of compound **4** in $CDCl_3$

Figure S38. HMBC spectrum of compound **4** in $CDCl_3$

Figure S39. HSQC spectrum of compound **4** in $CDCl_3$

Figure S40. NOESY spectrum of compound **4** in $CDCl_3$

Figure S41. HR-ESI-MS spectrum of compound **5**

Figure S42. OR valve of compound **5** in MeOH

Figure S43. UV spectrum of compound **5** in MeOH

Figure S44. CD spectrum of compound **5** in MeOH

Figure S45. $^1H$ NMR spectrum of compound **5** in $CDCl_3$

Figure S46. $^{13}C$ NMR and DEPT spectrum of compound **5** in $CDCl_3$

Figure S47. $^1H$-$^1H$ COSY spectrum of compound **5** in $CDCl_3$

Figure S48. HMBC spectrum of compound **5** in $CDCl_3$

Figure S49. HSQC spectrum of compound **5** in $CDCl_3$

Figure S50. NOESY spectrum of compound **5** in $CDCl_3$

Figure S51. HR-ESI-MS spectrum of compound **6**

Figure S52. OR valve of compound **6** in MeOH

Figure S53. UV spectrum of compound **6** in MeOH

Figure S54. CD spectrum of compound **6** in MeOH

Figure S55. $^1H$ NMR spectrum of compound **6** in $C_3D_6O$

Figure S56. $^{13}C$ NMR and DEPT spectrum of compound **6** in $C_3D_6O$

Figure S57. $^1H$ - $^1H$ COSY spectrum of compound **6** in $C_3D_6O$

Figure S58. HMBC spectrum of compound **6** in $C_3D_6O$

Figure S59. HSQC spectrum of compound **6** in $C_3D_6O$

Figure S60. NOESY spectrum of compound **6** in $C_3D_6O$

Figure S61. HR-ESI-MS spectrum of compound **7**

Figure S62. OR valve of compound **7** in MeOH

Figure S63. UV spectrum of compound **7** in MeOH

Figure S64. CD spectrum of compound **7** in MeOH

Figure S65. $^1H$ NMR spectrum of compound **7** in $CDCl_3$

Figure S66. $^{13}C$ NMR and DEPT spectrum of compound **7** in $CDCl_3$

Figure S67. $^1H$-$^1H$ COSY spectrum of compound **7** in $CDCl_3$

Figure S68. HMBC spectrum of compound **7** in $CDCl_3$

Figure S69. HSQC spectrum of compound **7** in $CDCl_3$

Figure S70. NOESY spectrum of compound **7** in $CDCl_3$

Figure S71. HR-ESI-MS spectrum of compound **8**

Figure S72. OR Valve of compound **8** in MeOH

Figure S73. UV spectrum of compound **8** in MeOH

Figure S74. CD spectrum of compound **8** in MeOH

Figure S75. $^1H$ NMR spectrum of compound **8** in $CDCl_3$

Figure S76. $^{13}C$ NMR and DEPT spectrum of compound **8** in $CDCl_3$

Figure S77. $^1H$-$^1H$ COSY spectrum of compound **8** in $CDCl_3$

Figure S78. HMBC spectrum of compound **8** in $CDCl_3$

Figure S79. HSQC spectrum of compound **8** in $CDCl_3$

Figure S80. NOESY spectrum of compound **8** in $CDCl_3$

Figure S81. HR-ESI-MS spectrum of compound **9**

Figure S82. OR Valve of compound **9** in MeOH

Figure S83. UV spectrum of compound **9** in MeOH

Figure S84. CD spectrum of compound **9** in MeOH

Figure S85. $^1H$ NMR spectrum of compound **9** in $CDCl_3$

Figure S86. $^{13}C$ NMR and DEPT spectrum of compound **9** in $CDCl_3$

Figure S87. $^{1}$H - $^{1}$H COSY spectrum of compound **9** in $CDCl_3$

Figure S88. HMBC spectrum of compound **9** in $CDCl_3$

Figure S89. HSQC spectrum of compound **9** in $CDCl_3$

Figure S90. NOESY spectrum of compound **9** in $CDCl_3$

Figure S91. HR-ESI-MS spectrum of compound **10**

Figure S92. $^{1}$H NMR spectrum of compound **10** in $CDCl_3$

Figure S93. $^{13}$C NMR and DEPT spectrum of compound **10** in $CDCl_3$

Figure S94. HR-ESI-MS spectrum of compound **11**

Figure S95. OR valve of compound **11** in MeOH

Figure S96. UV spectrum of compound **11** in MeOH

Figure S97. CD spectrum of compound **11** in MeOH

Figure S98. $^{1}$H NMR spectrum of compound **11** in $CDCl_3$

Figure S99. $^{13}$C NMR and DEPT spectrum of compound **11** in $CDCl_3$

Figure S100. $^{1}$H-$^{1}$H COSY spectrum of compound **11** in $CDCl_3$

Figure S101. HMBC spectrum of compound **11** in $CDCl_3$

Figure S102. HSQC spectrum of compound **11** in $CDCl_3$

Figure S103. NOESY spectrum of compound **11** in $CDCl_3$

Figure S104. HR-ESI-MS spectrum of compound **12**

Figure S105. OR valve of compound **12** in MeOH

Figure S106. UV spectrum of compound **12** in MeOH

Figure S107. CD spectrum of compound **12** in MeOH

Figure S108. $^{1}$H NMR spectrum of compound **12** in $CDCl_3$

Figure S109. $^{13}$C NMR and DEPT spectrum of compound **12** in $CDCl_3$

Figure S110. $^{1}$H - $^{1}$H COSY spectrum of compound **12** in $CDCl_3$

Figure S111. HMBC spectrum of compound **12** in $CDCl_3$

Figure S112. HSQC spectrum of compound **12** in $CDCl_3$

Figure S113. NOESY spectrum of compound **12** in $CDCl_3$

Table S1. Effects of metabolites on proliferation of four strains of human cells (Inhibition rate %)

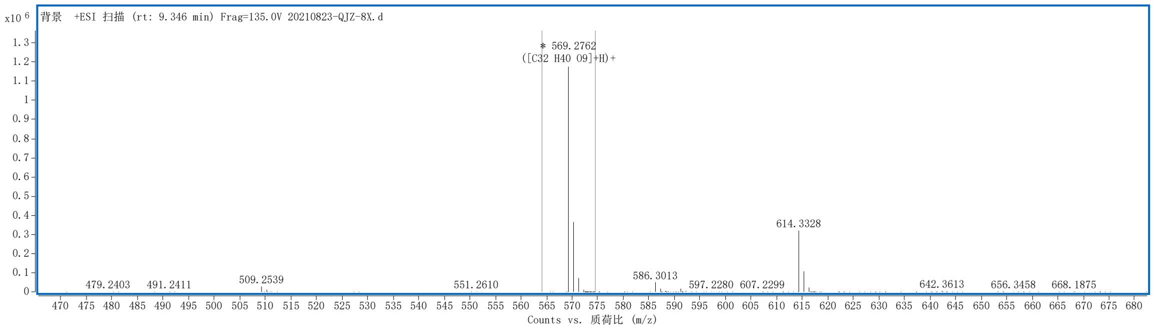


| Best | Formula(M) | Ion Formula | Tgt (m/z) | m/z | Diff (ppm) |
|---|---|---|---|---|---|
| TRUE | $C_{32}H_{40}O_9$ | $C_{32}H_{41}O_9$ | 569.2745 | 569.2762 | 2.99 |

Figure S1. HR-ESI-MS spectrum of compound **1**

**Rudolph Research Analytical**

Monday, 10/11/2021

This sample was measured on an Autopol VI, serial number 90079,
manufactured by Rudolph Research Analytical,Hackettstown,NJ.

LotID : QJZ-8
Set Temperature : 20.0
Temp Corr : OFF

| n | Average | Std.Dev. | Maximum | Minimum |
|---|---|---|---|---|
| 6 | 116.102 | 0.0000 | 116.102 | 116.102 |

| S.No | Sample ID | Time | Result | Scale | OR °Arc | WLG | Lg.mm | Conc. | Temp. | Comment |
|---|---|---|---|---|---|---|---|---|---|---|
| 1 | QJZ-8 | 10:45:38 AM | 116.102 | SR | 0.137 | 589 | 100.00 | 0.118 | 19.7 | |
| 2 | QJZ-8 | 10:45:45 AM | 116.102 | SR | 0.137 | 589 | 100.00 | 0.118 | 19.7 | |
| 3 | QJZ-8 | 10:45:53 AM | 116.102 | SR | 0.137 | 589 | 100.00 | 0.118 | 19.7 | |
| 4 | QJZ-8 | 10:46:00 AM | 116.102 | SR | 0.137 | 589 | 100.00 | 0.118 | 19.7 | |
| 5 | QJZ-8 | 10:46:08 AM | 116.102 | SR | 0.137 | 589 | 100.00 | 0.118 | 19.8 | |
| 6 | QJZ-8 | 10:46:15 AM | 116.102 | SR | 0.137 | 589 | 100.00 | 0.118 | 19.8 | |

______________________________
Signature

Figure S2. OR valve of compound **1** in MeOH

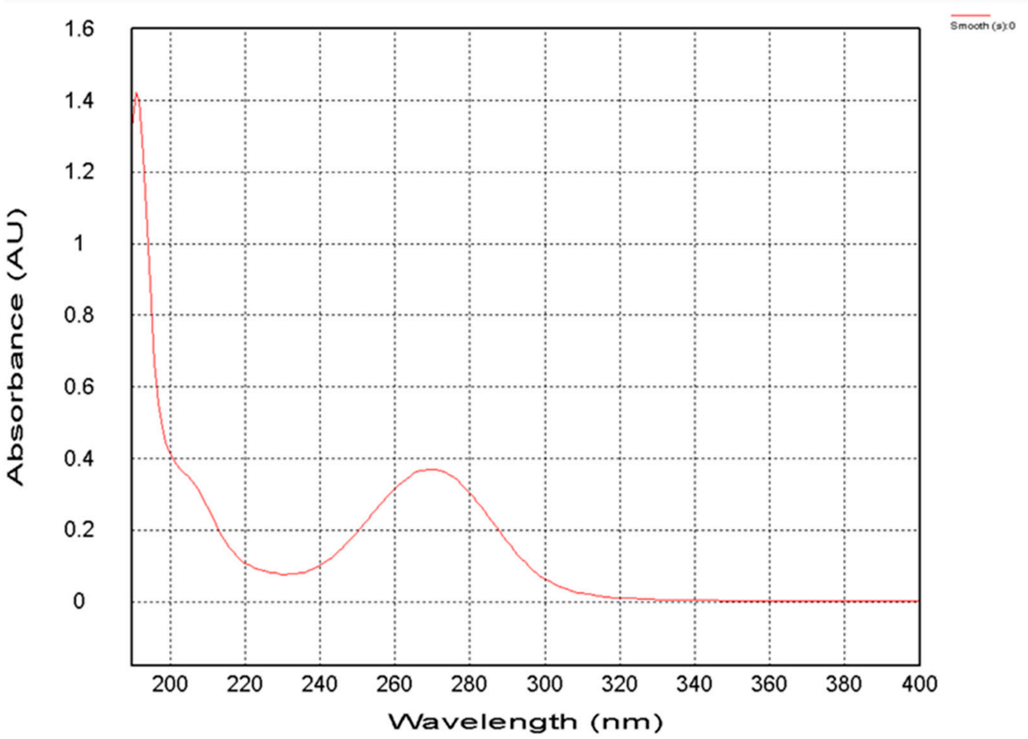


Figure S3. UV spectrum of compound **1** in MeOH

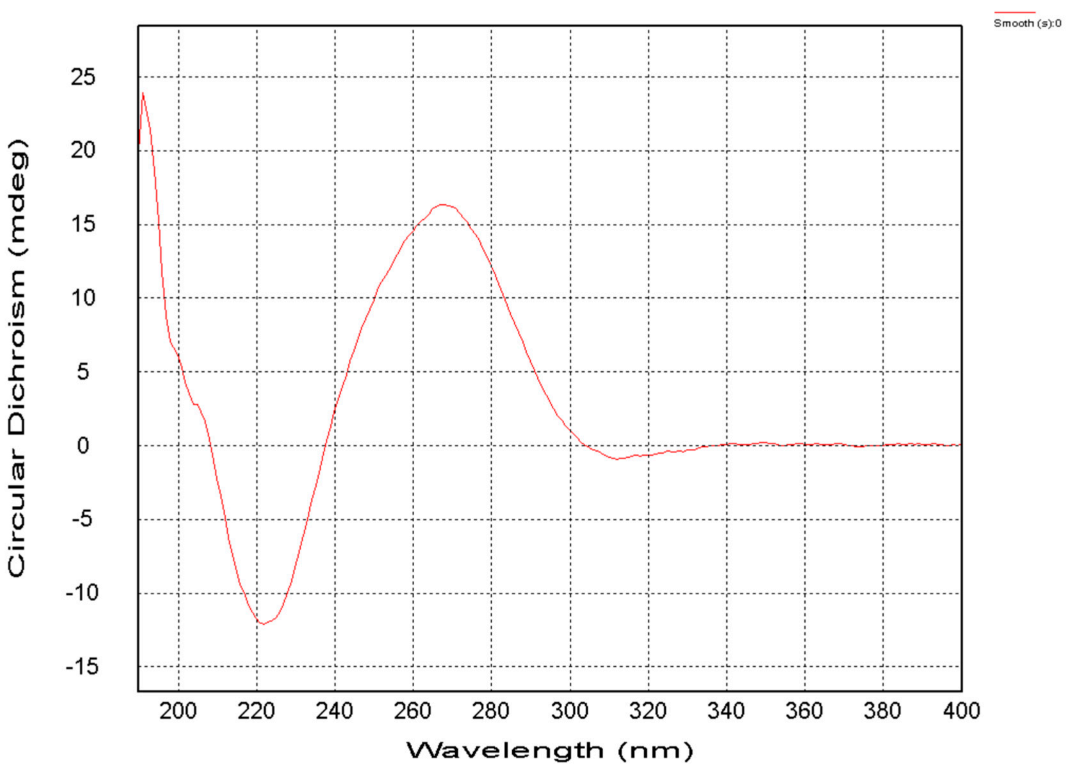


Figure S4. CD spectrum of compound **1** in MeOH

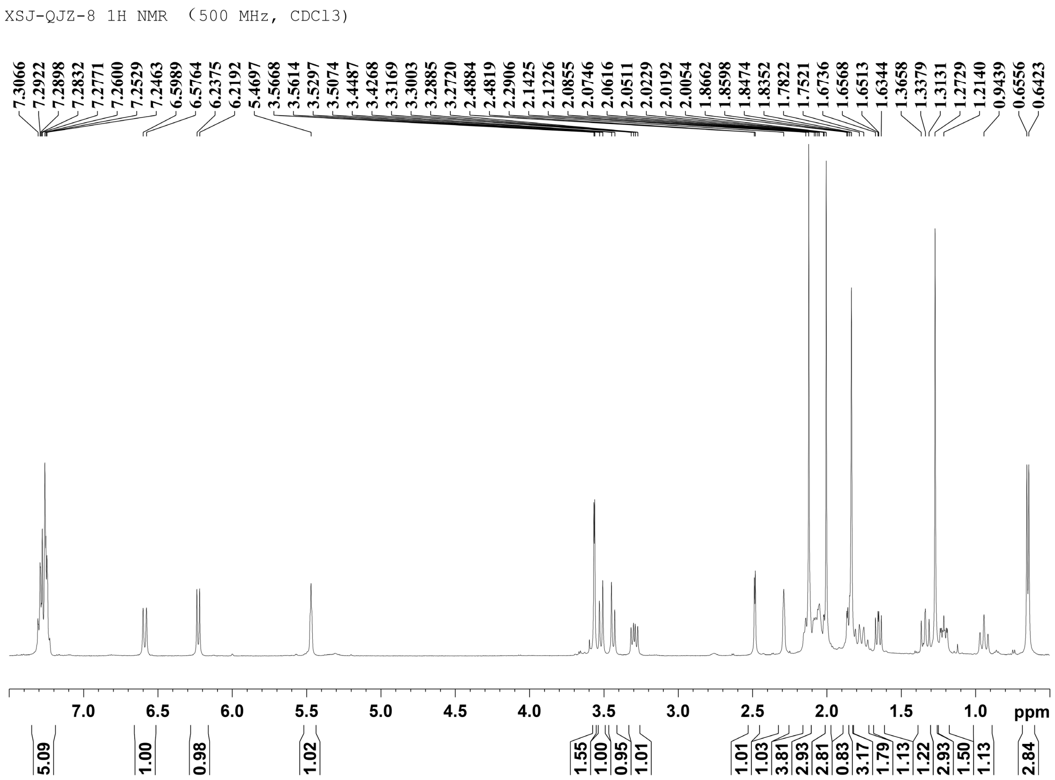


Figure S5. $^{1}H$ NMR spectrum of compound **1** in $CDCl_3$

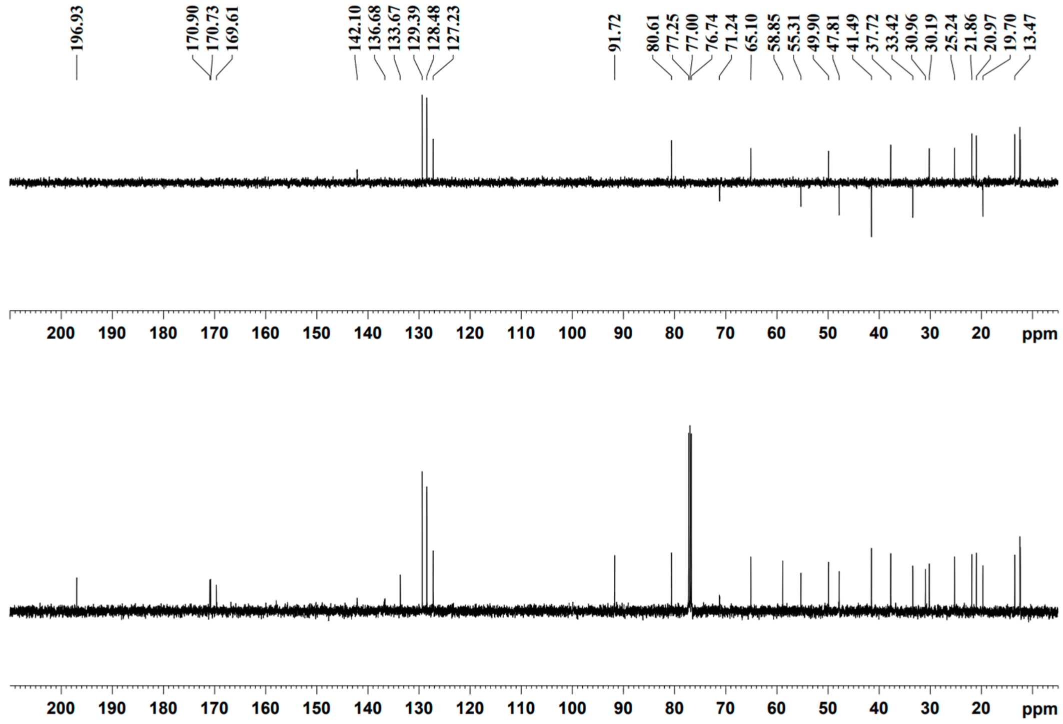


Figure S6. $^{13}C$ NMR and DEPT spectrum of compound **1** in $CDCl_3$

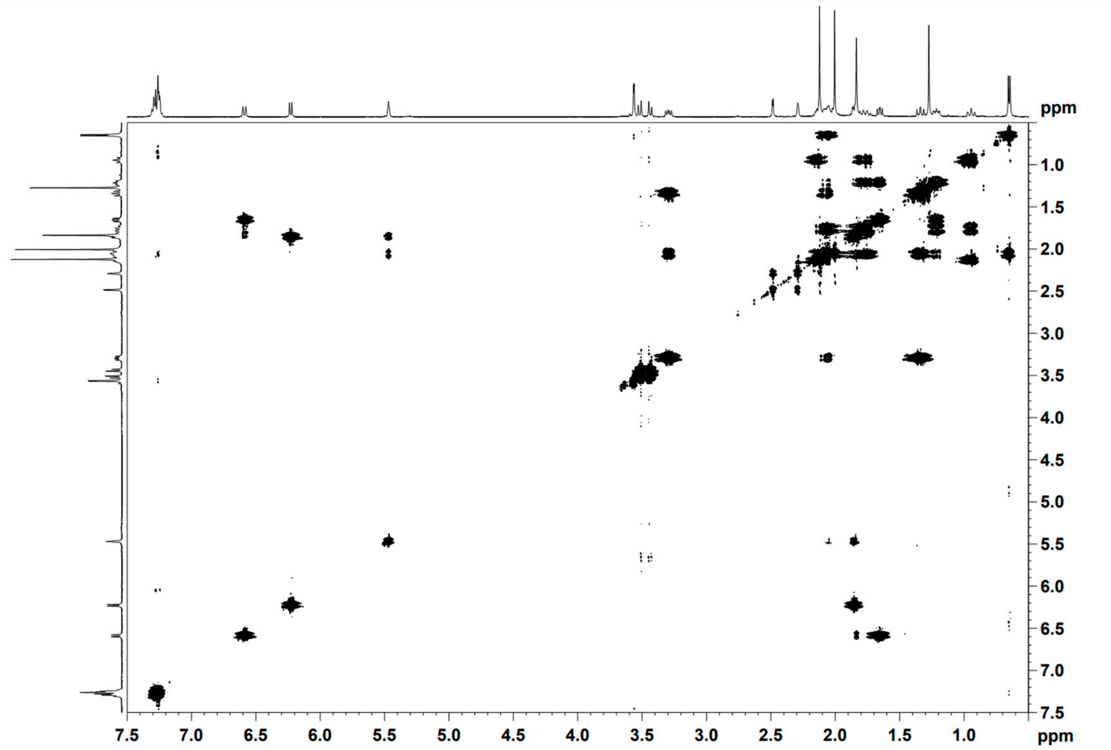


Figure S7. $^{1}$H-$^{1}$H COSY spectrum of compound **1** in $CDCl_3$

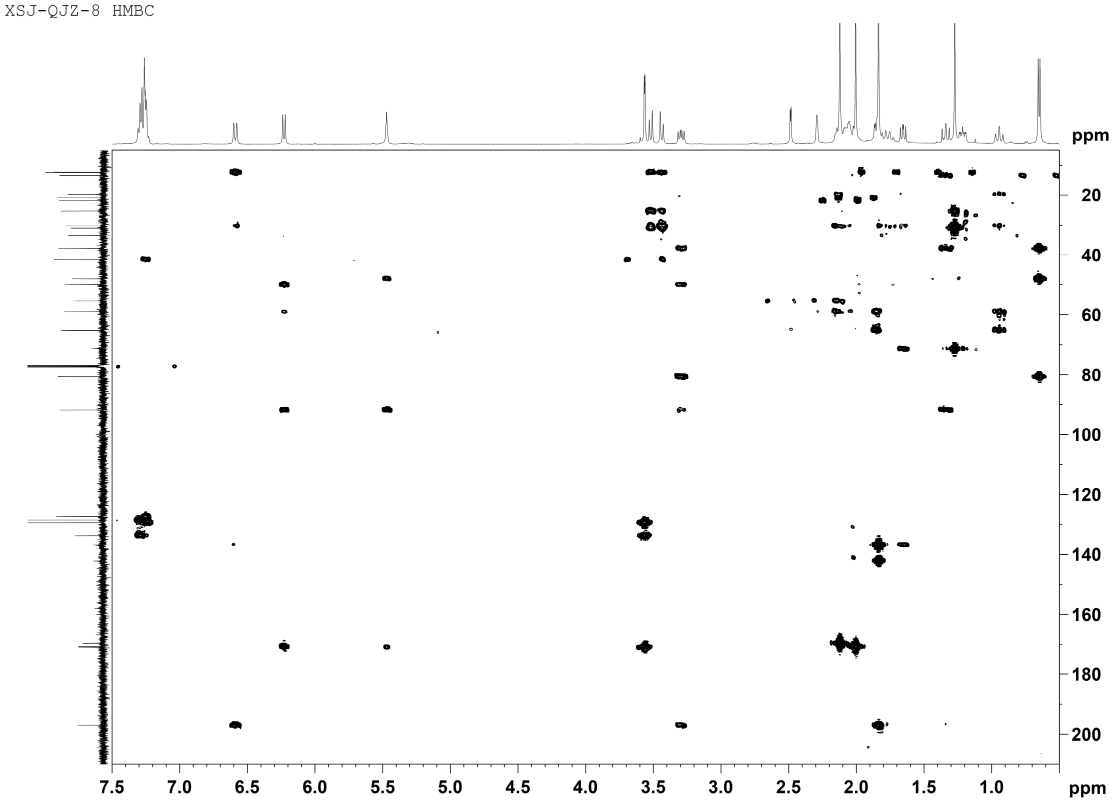


Figure S8. HMBC spectrum of compound **1** in $CDCl_3$

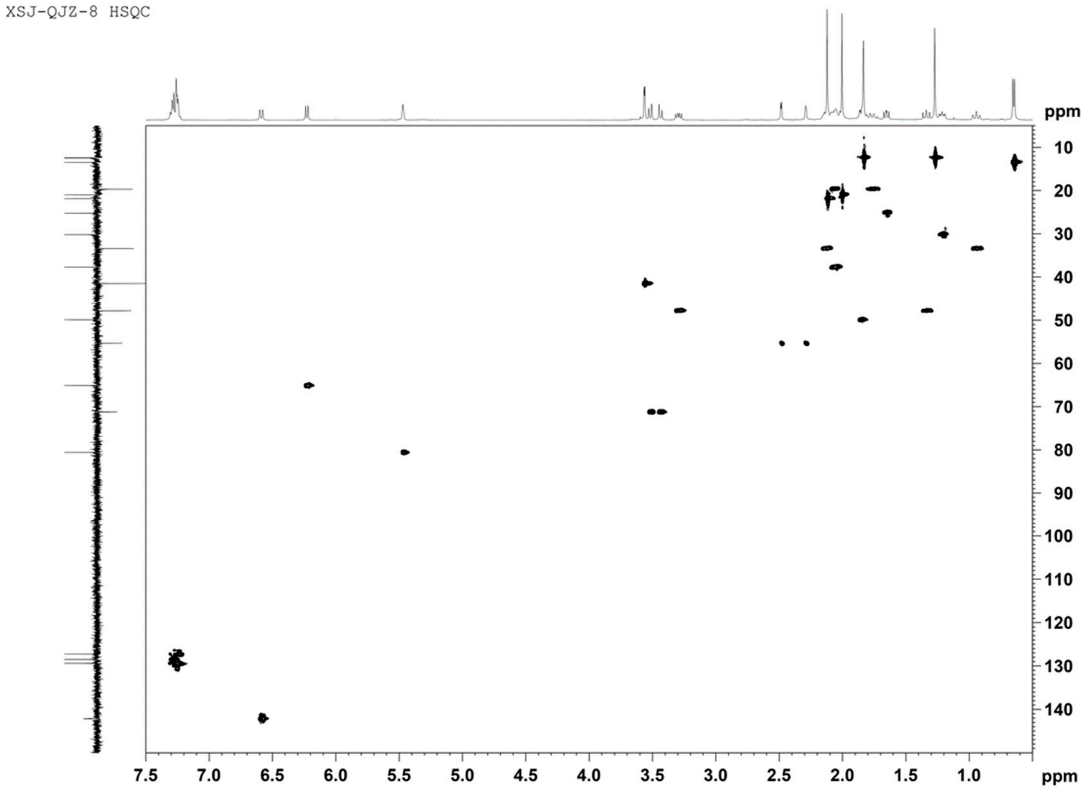


Figure S9. HSQC spectrum of compound **1** in $CDCl_3$

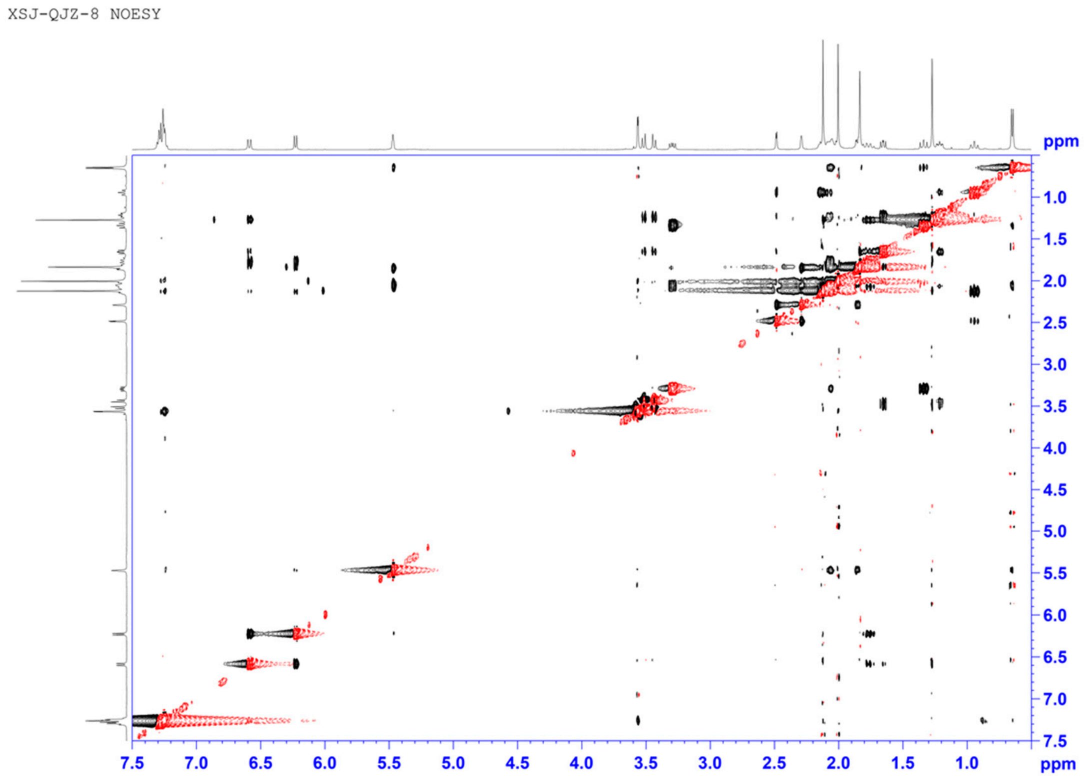


Figure S10. NOESY spectrum of compound **1** in $CDCl_3$

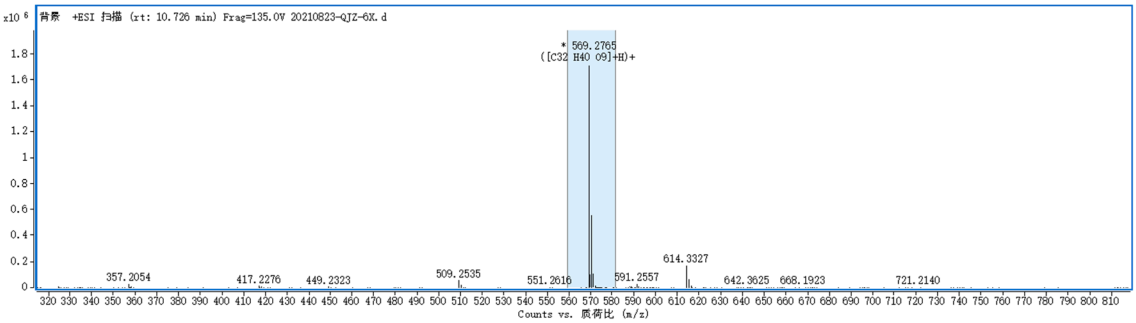


| Best | Formula(M) | Ion Formula | Tgt (m/z) | m/z | Diff (ppm) |
|---|---|---|---|---|---|
| TRUE | $C_{32}H_{40}O_9$ | $C_{32}H_{41}O_9$ | 569.2745 | 569.2765 | 3.51 |

Figure S11. HR-ESI-MS spectrum of compound **2**

**Rudolph Research Analytical**

Monday, 10/11/2021

This sample was measured on an Autopol VI, serial number 90079, manufactured by Rudolph Research Analytical,Hackettstown,NJ.

LotID : QJZ-6
Set Temperature : 20.0
Temp Corr : OFF

| n | Average | Std.Dev. | Maximum | Minimum |
|---|---|---|---|---|
| 6 | 74.889 | 0.3140 | 75.333 | 74.667 |

| S.No | Sample ID | Time | Result | Scale | OR ° Arc | WLG | Lg.mm | Conc. | Temp. | Comment |
|---|---|---|---|---|---|---|---|---|---|---|
| 1 | QJZ-6 | 11:09:52 AM | 75.333 | SR | 0.113 | 589 | 100.00 | 0.150 | 19.7 | |
| 2 | QJZ-6 | 11:10:00 AM | 75.333 | SR | 0.113 | 589 | 100.00 | 0.150 | 19.7 | |
| 3 | QJZ-6 | 11:10:08 AM | 74.667 | SR | 0.112 | 589 | 100.00 | 0.150 | 19.7 | |
| 4 | QJZ-6 | 11:10:15 AM | 74.667 | SR | 0.112 | 589 | 100.00 | 0.150 | 19.8 | |
| 5 | QJZ-6 | 11:10:23 AM | 74.667 | SR | 0.112 | 589 | 100.00 | 0.150 | 19.8 | |
| 6 | QJZ-6 | 11:10:30 AM | 74.667 | SR | 0.112 | 589 | 100.00 | 0.150 | 19.8 | |

______________________
Signature

Figure S12. OR valve of compound **2** in MeOH

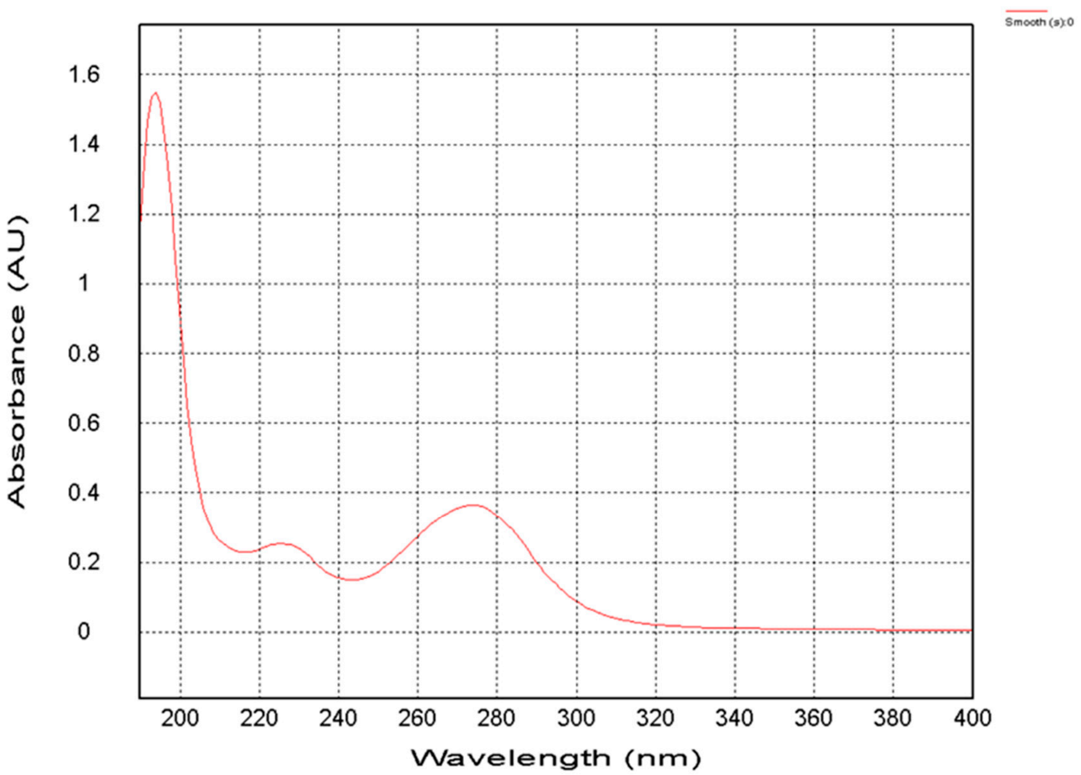


Figure S13. UV spectrum of compound **2** in MeOH

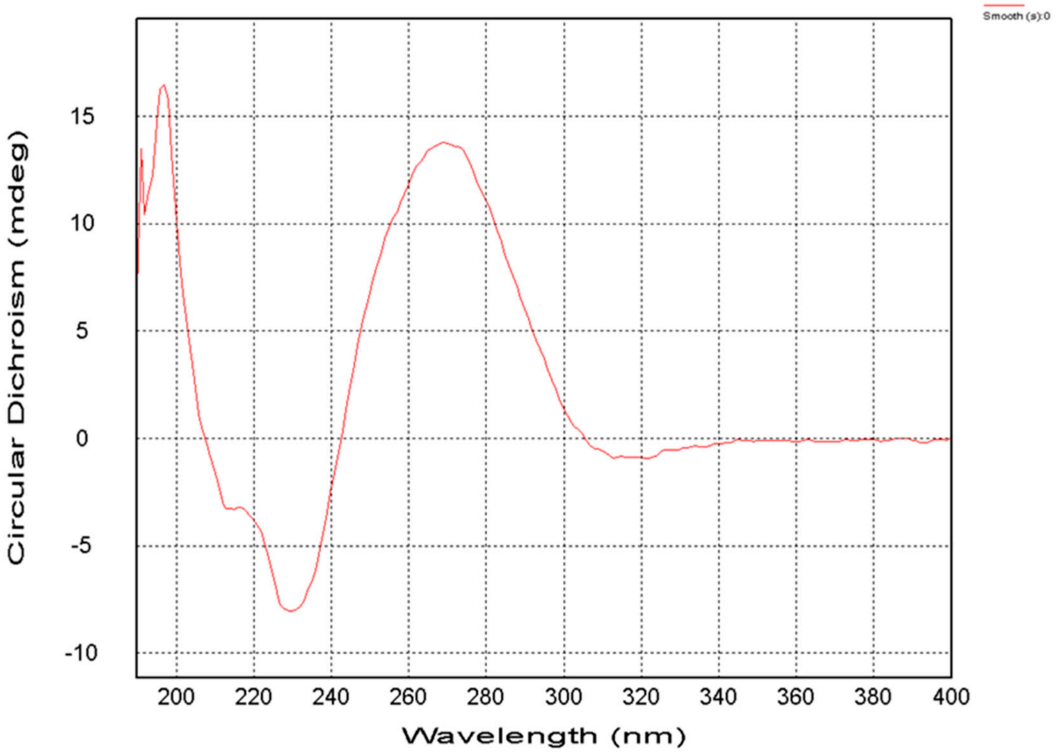


Figure S14. CD spectrum of compound **2** in MeOH

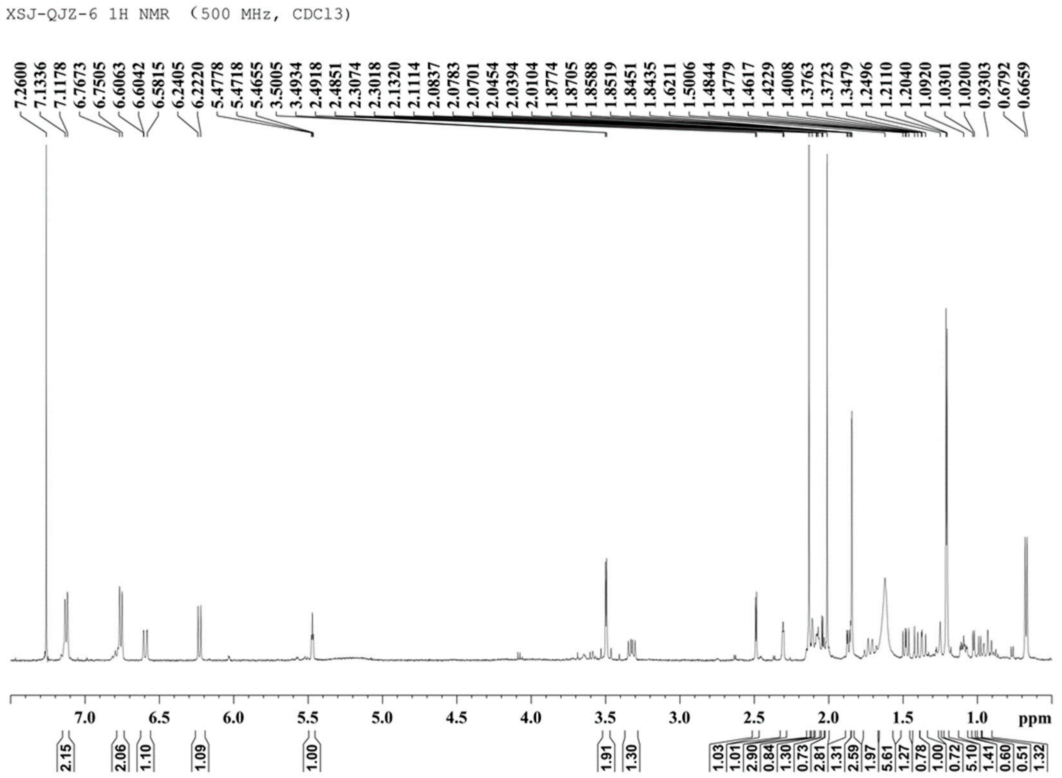


Figure S15. $^{1}$H NMR spectrum of compound **2** in $CDCl_3$

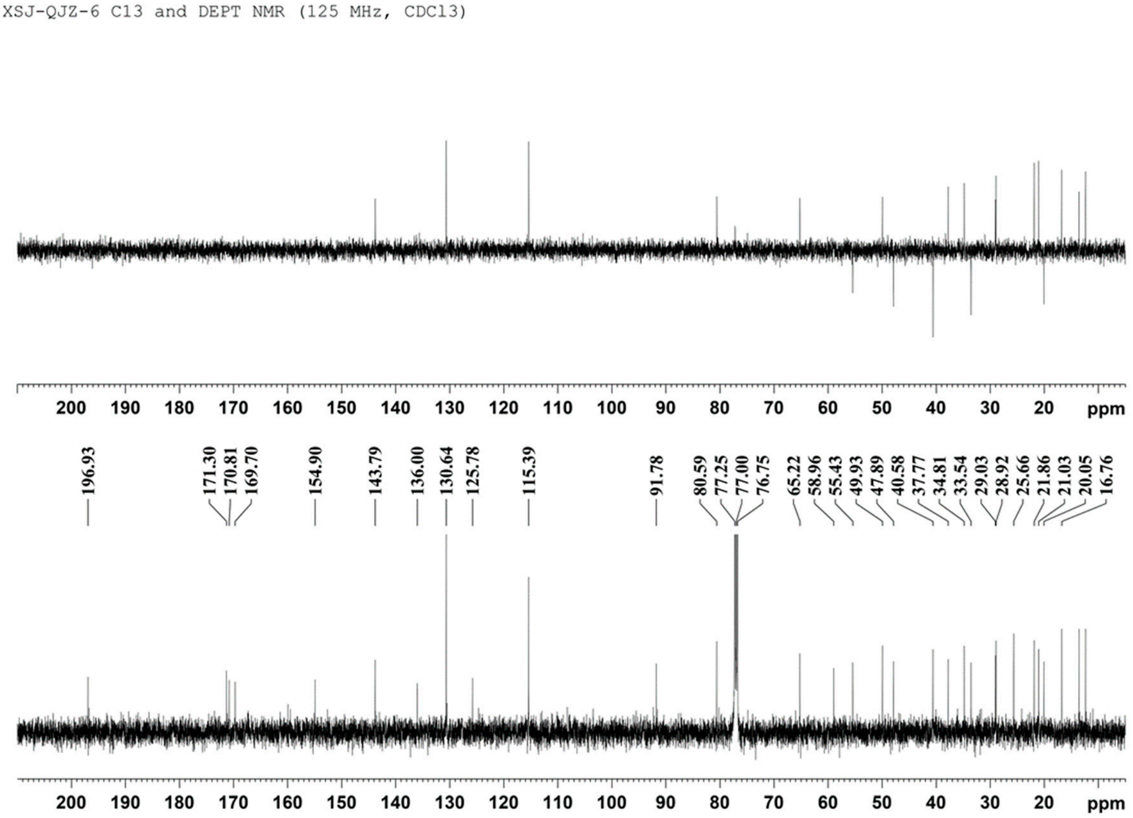


Figure S16. $^{13}$C NMR and DEPT spectrum of compound **2** in $CDCl_3$

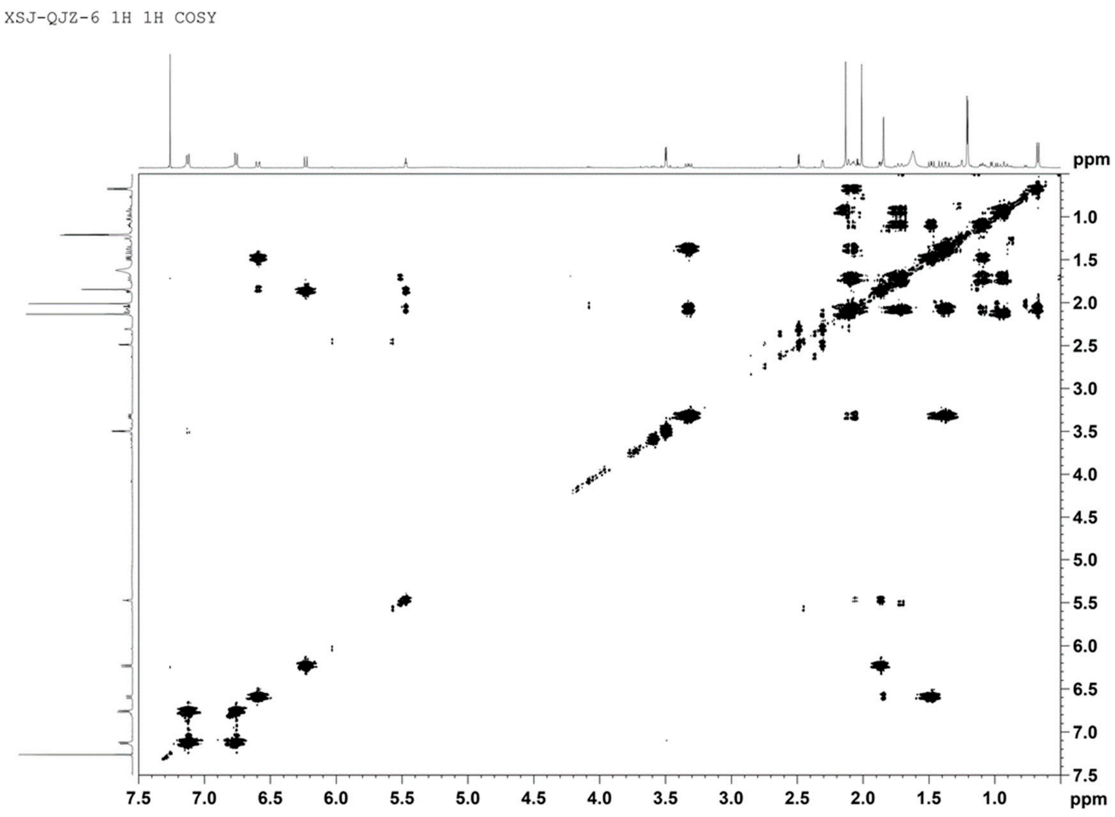


Figure S17. $^1H$-$^1H$ COSY spectrum of compound **2** in $CDCl_3$

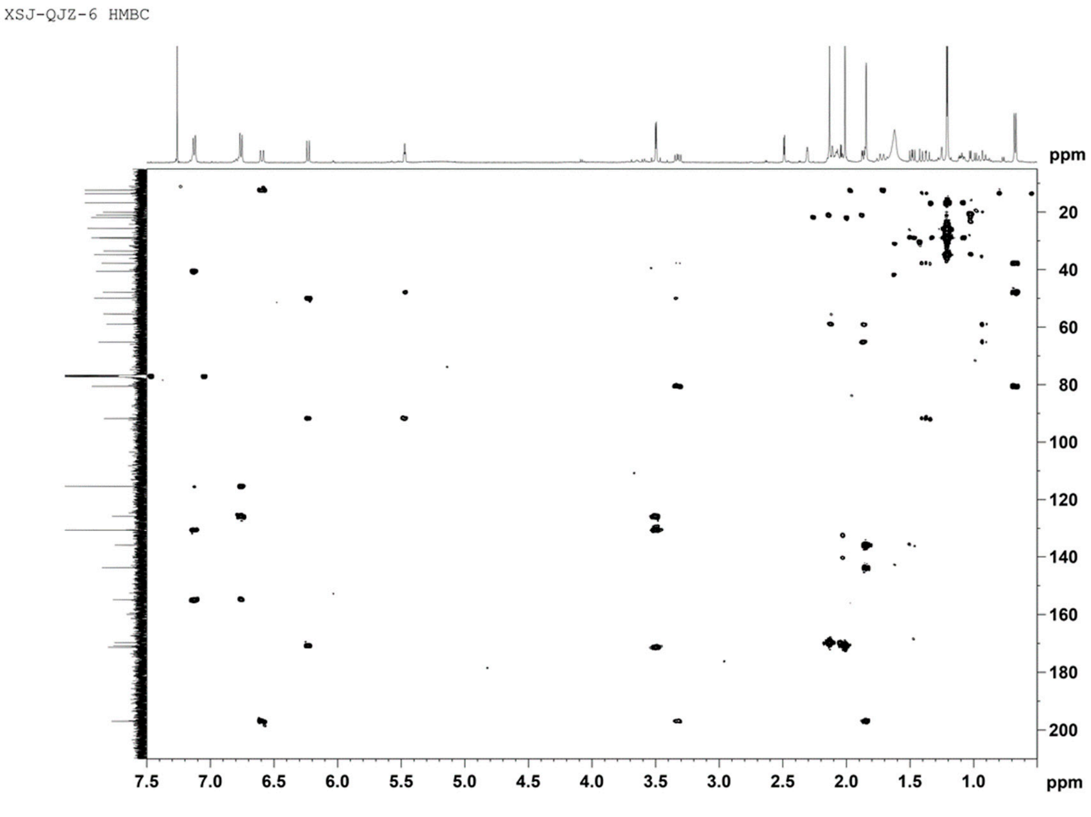


Figure S18. HMBC spectrum of compound **2** in $CDCl_3$

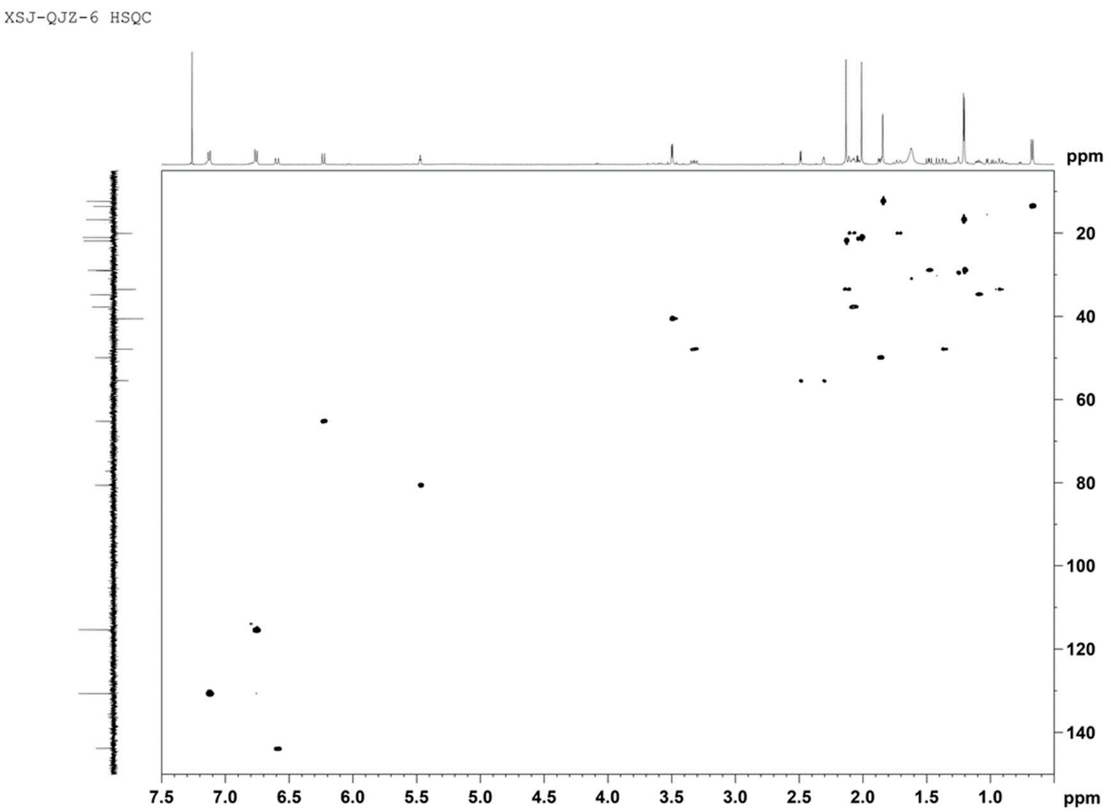


Figure S19. HSQC spectrum of compound **2** in $CDCl_3$

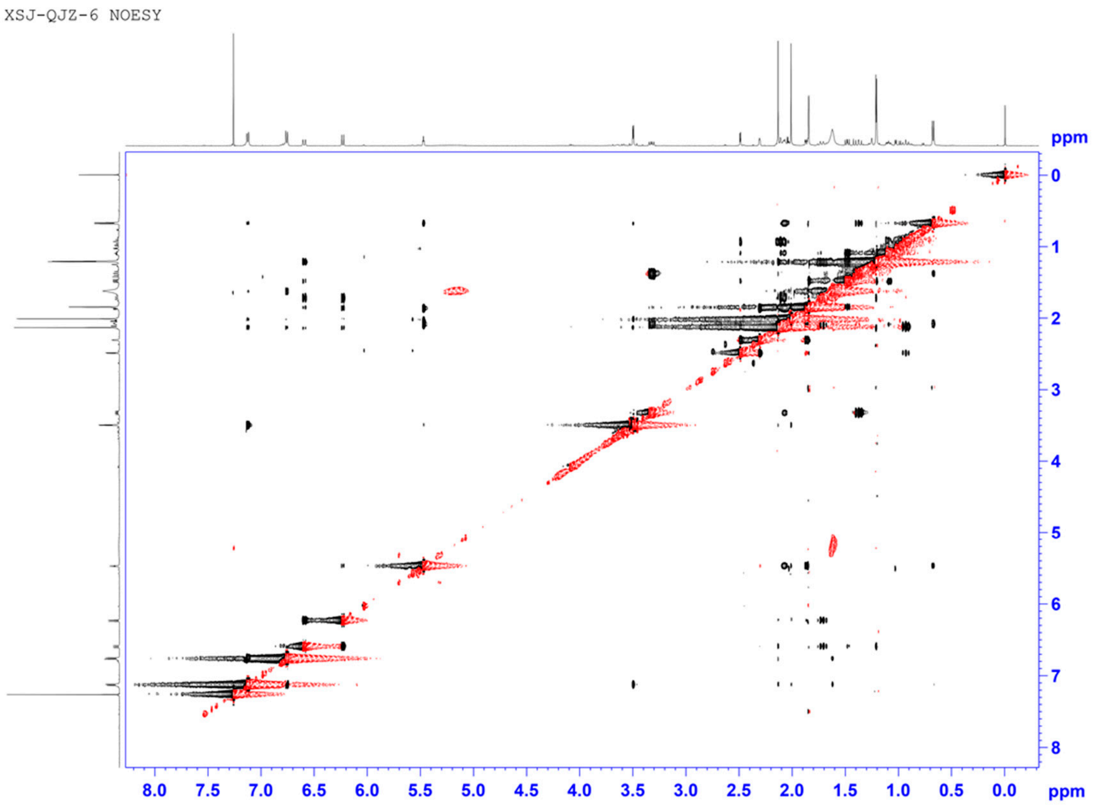


Figure S20. NOESY spectrum of compound **2** in $CDCl_3$

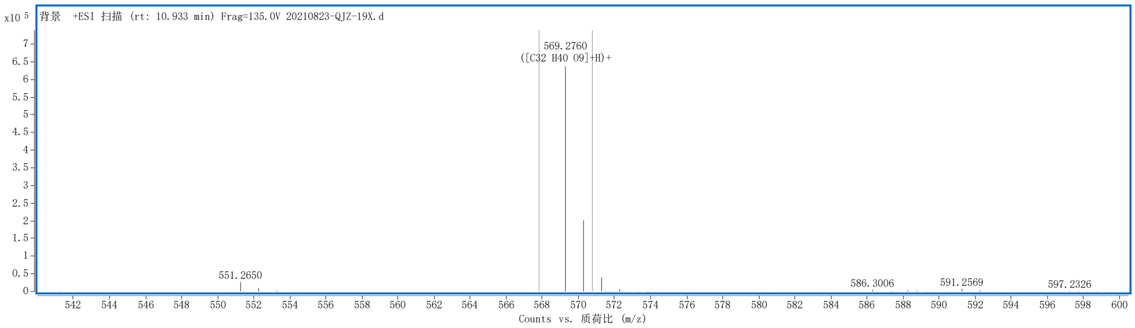


| Best | Formula(M) | Ion Formula | Tgt (m/z) | m/z | Diff (ppm) |
|---|---|---|---|---|---|
| TRUE | $C_{32}H_{40}O_9$ | $C_{32}H_{41}O_9$ | 569.2745 | 569.2760 | 2.63 |

Figure S21. HR-ESI-MS spectrum of compound **3**

**Rudolph Research Analytical**

Saturday, 10/09/2021

This sample was measured on an Autopol VI, serial number 90079,
manufactured by Rudolph Research Analytical,Hackettstown,NJ.

LotID : QJZ-19
Set Temperature : 20.0
Temp Corr : OFF

| n | Average | Std.Dev. | Maximum | Minimum |
|---|---|---|---|---|
| 6 | 63.974 | 0.2866 | 64.615 | 63.846 |

| S.No | Sample ID | Time | Result | Scale | OR °Arc | WLG | Lg.mm | Conc. | Temp. | Comment |
|---|---|---|---|---|---|---|---|---|---|---|
| 1 | QJZ-19 | 04:13:14 PM | 64.615 | SR | 0.084 | 589 | 100.00 | 0.130 | 19.7 | |
| 2 | QJZ-19 | 04:13:21 PM | 63.846 | SR | 0.083 | 589 | 100.00 | 0.130 | 19.7 | |
| 3 | QJZ-19 | 04:13:28 PM | 63.846 | SR | 0.083 | 589 | 100.00 | 0.130 | 19.7 | |
| 4 | QJZ-19 | 04:13:36 PM | 63.846 | SR | 0.083 | 589 | 100.00 | 0.130 | 19.7 | |
| 5 | QJZ-19 | 04:13:43 PM | 63.846 | SR | 0.083 | 589 | 100.00 | 0.130 | 19.7 | |
| 6 | QJZ-19 | 04:13:51 PM | 63.846 | SR | 0.083 | 589 | 100.00 | 0.130 | 19.8 | |

____________________________
Signature

Figure S22. OR value of compound **3** in MeOH

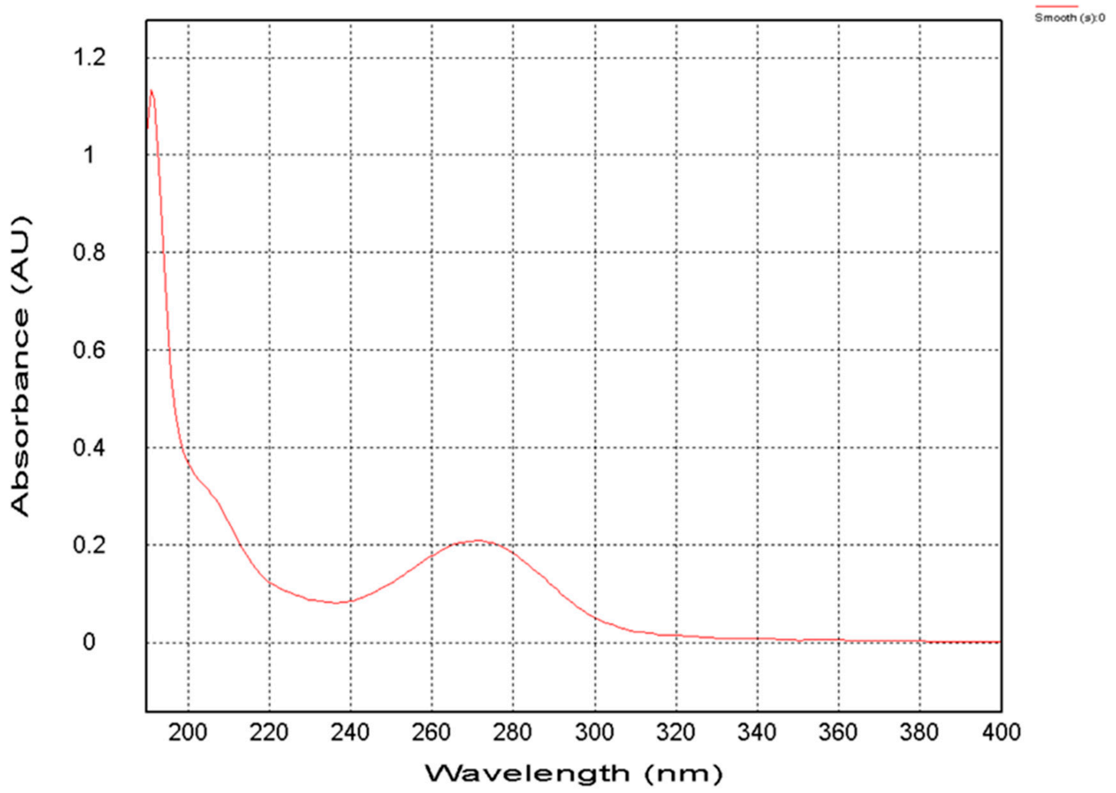


Figure S23. UV spectrum of compound **3** in MeOH

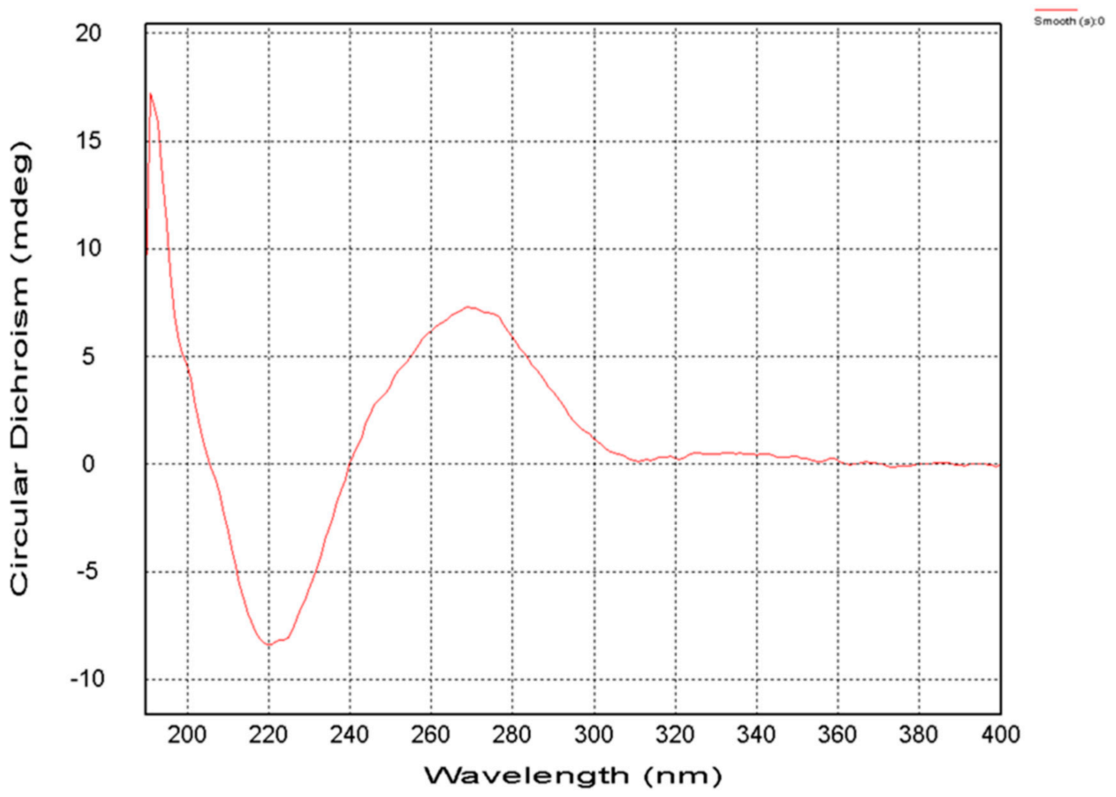


Figure S24. CD spectrum of compound **3** in MeOH

XSJ-QJZ-19 1H NMR (500 MHz, CDCl3)

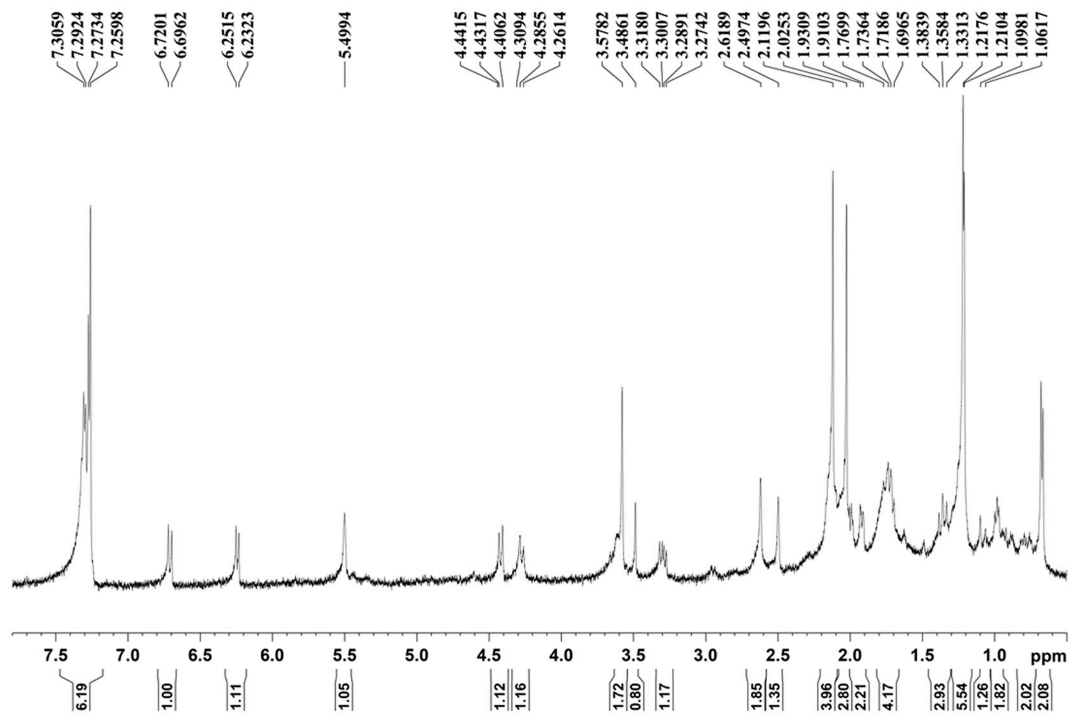


Figure S25. $^{1}H$ NMR spectrum of compound **3** in $CDCl_3$

XSJ-QJZ-19 C13 and DEPT NMR (125 MHz, CDCl3)

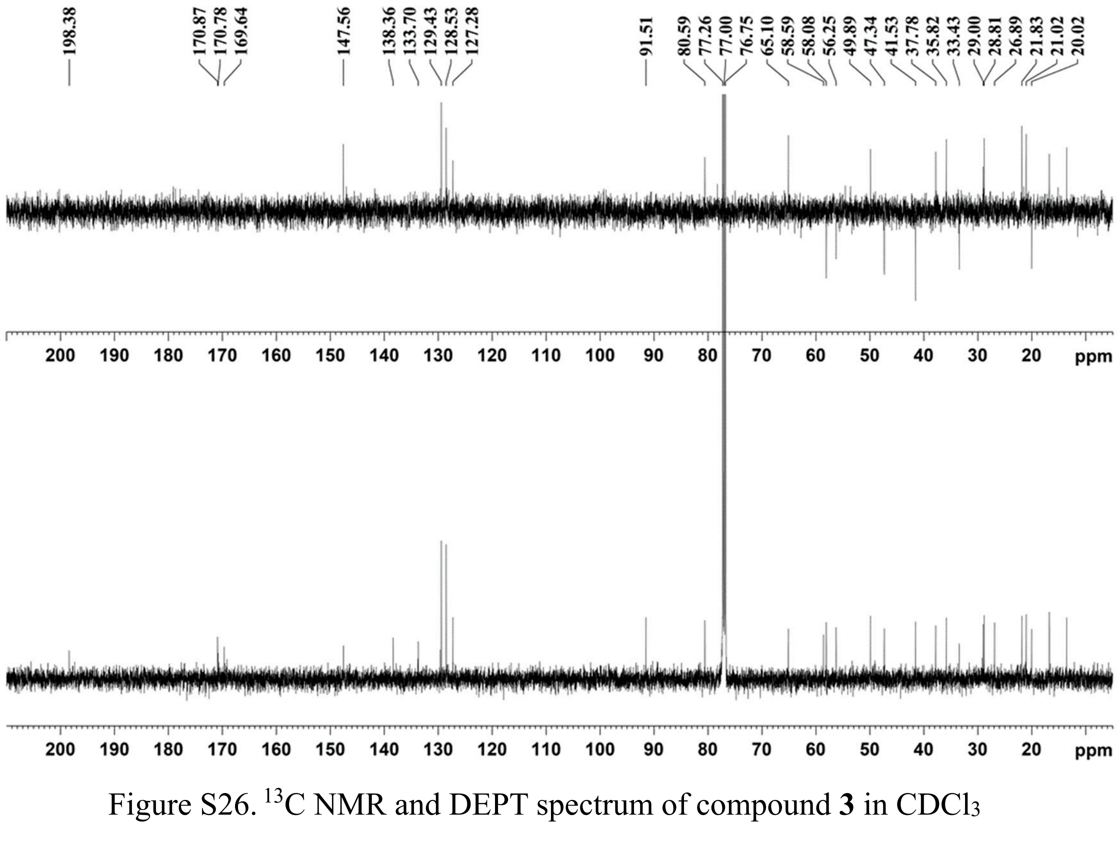


Figure S26. $^{13}C$ NMR and DEPT spectrum of compound **3** in $CDCl_3$

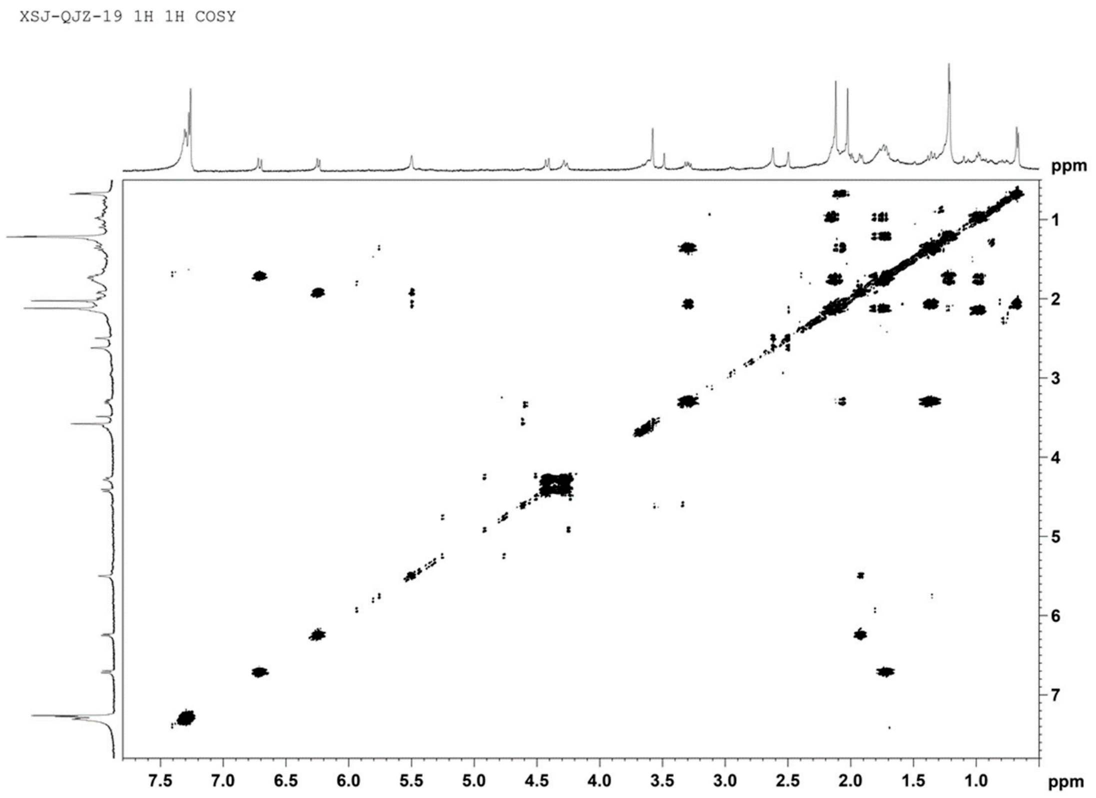


Figure S27. $^{1}H$-$^{1}H$ COSY spectrum of compound **3** in $CDCl_3$

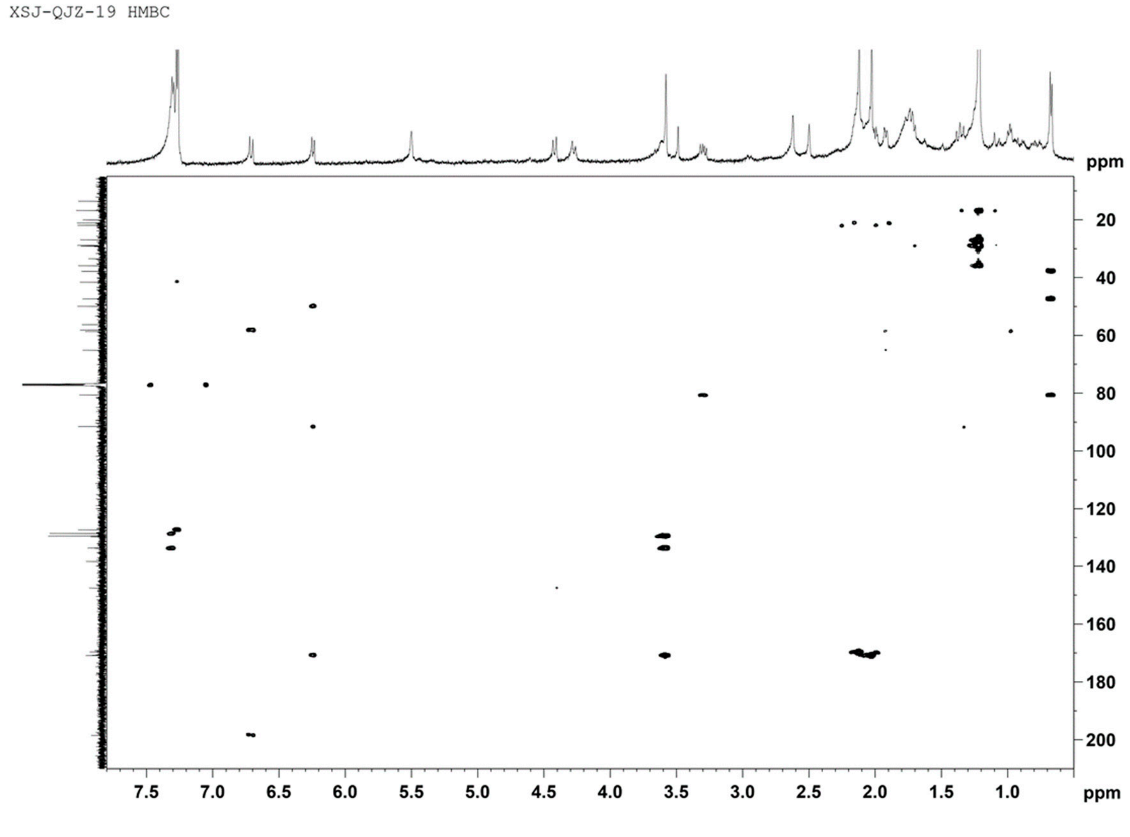


Figure S28. HMBC spectrum of compound **3** in $CDCl_3$

XSJ-QJZ-19 HSQC

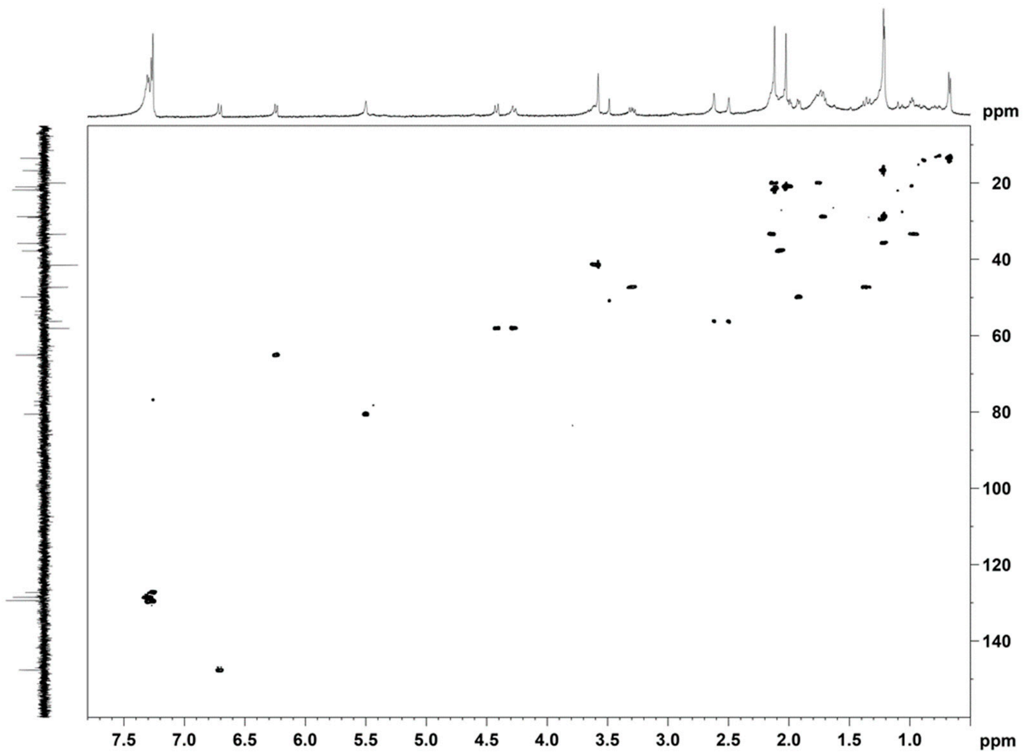


Figure S29. HSQC spectrum of compound **3** in $CDCl_3$

XSJ-QJZ-19 NOESY

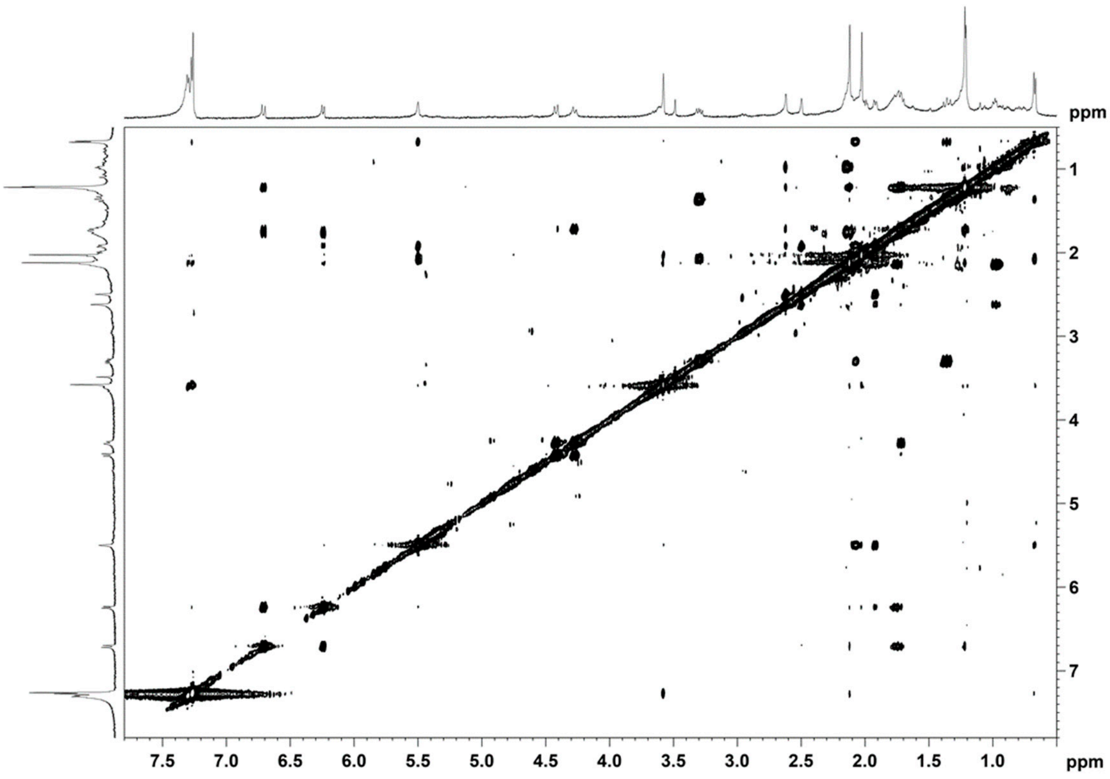


Figure S30. NOESY spectrum of compound **3** in $CDCl_3$

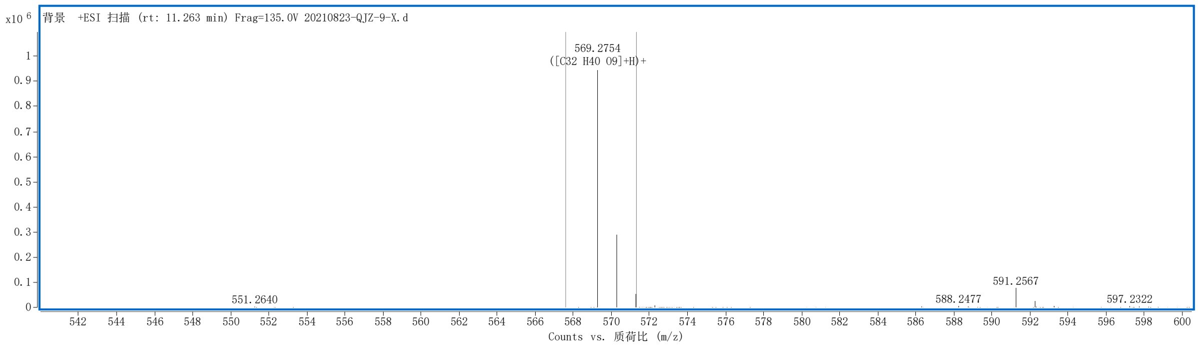


| Best | Formula(M) | Ion Formula | Tgt (m/z) | m/z | Diff (ppm) |
|---|---|---|---|---|---|
| TRUE | $C_{32}H_{40}O_9$ | $C_{32}H_{41}O_9$ | 569.2745 | 569.2754 | 1.58 |

Figure S31. HR-ESI-MS spectrum of compound **4**

**Rudolph Research Analytical**

Monday, 10/11/2021

This sample was measured on an Autopol VI, serial number 90079,
manufactured by Rudolph Research Analytical,Hackettstown,NJ.

LotID : QJZ-9
Set Temperature : 20.0
Temp Corr : OFF

| n | Average | Std.Dev. | Maximum | Minimum |
|---|---|---|---|---|
| 6 | 70.000 | 0.4000 | 70.400 | 69.600 |

| S.No | Sample ID | Time | Result | Scale | OR °Arc | WLG | Lg.mm | Conc. | Temp. | Comment |
|---|---|---|---|---|---|---|---|---|---|---|
| 1 | QJZ-9 | 10:32:39 AM | 69.600 | SR | 0.087 | 589 | 100.00 | 0.125 | 20.2 | |
| 2 | QJZ-9 | 10:32:47 AM | 69.600 | SR | 0.087 | 589 | 100.00 | 0.125 | 20.1 | |
| 3 | QJZ-9 | 10:32:54 AM | 69.600 | SR | 0.087 | 589 | 100.00 | 0.125 | 20.1 | |
| 4 | QJZ-9 | 10:33:01 AM | 70.400 | SR | 0.088 | 589 | 100.00 | 0.125 | 20.1 | |
| 5 | QJZ-9 | 10:33:09 AM | 70.400 | SR | 0.088 | 589 | 100.00 | 0.125 | 20.0 | |
| 6 | QJZ-9 | 10:33:20 AM | 70.400 | SR | 0.088 | 589 | 100.00 | 0.125 | 20.0 | |

______________________________
Signature

Figure S32. OR value of compound **4** in MeOH

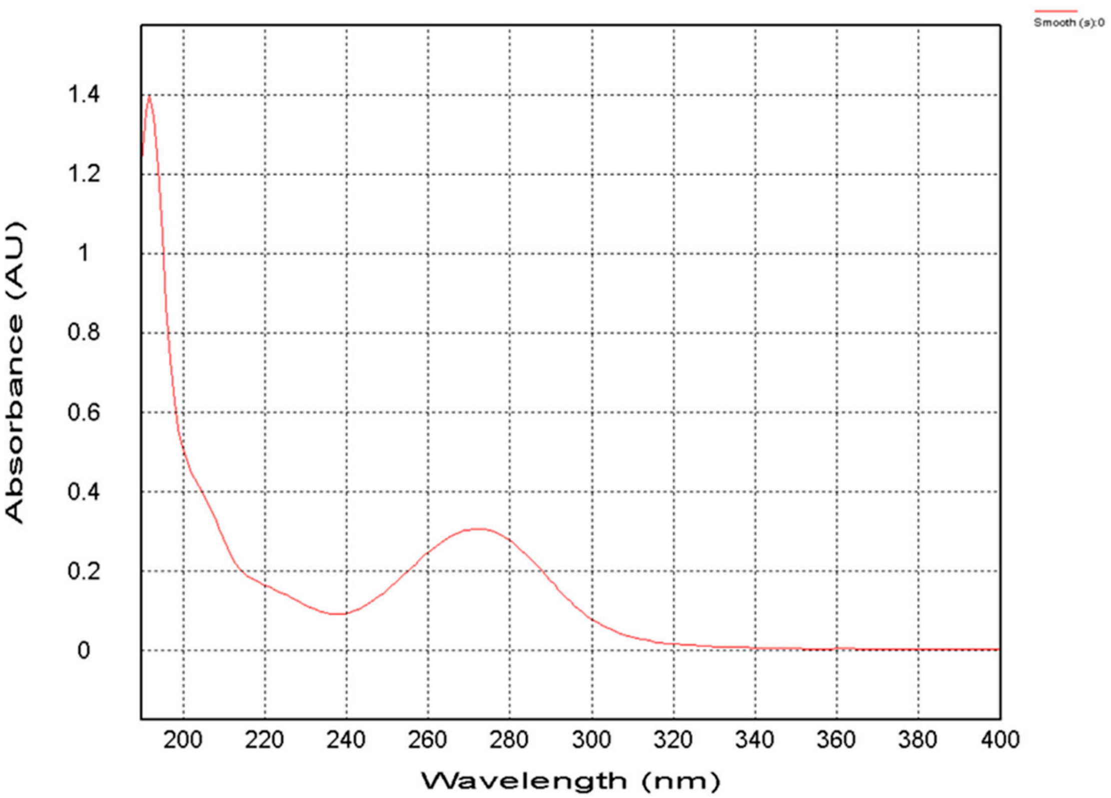


Figure S33. UV spectrum of compound **4** in MeOH

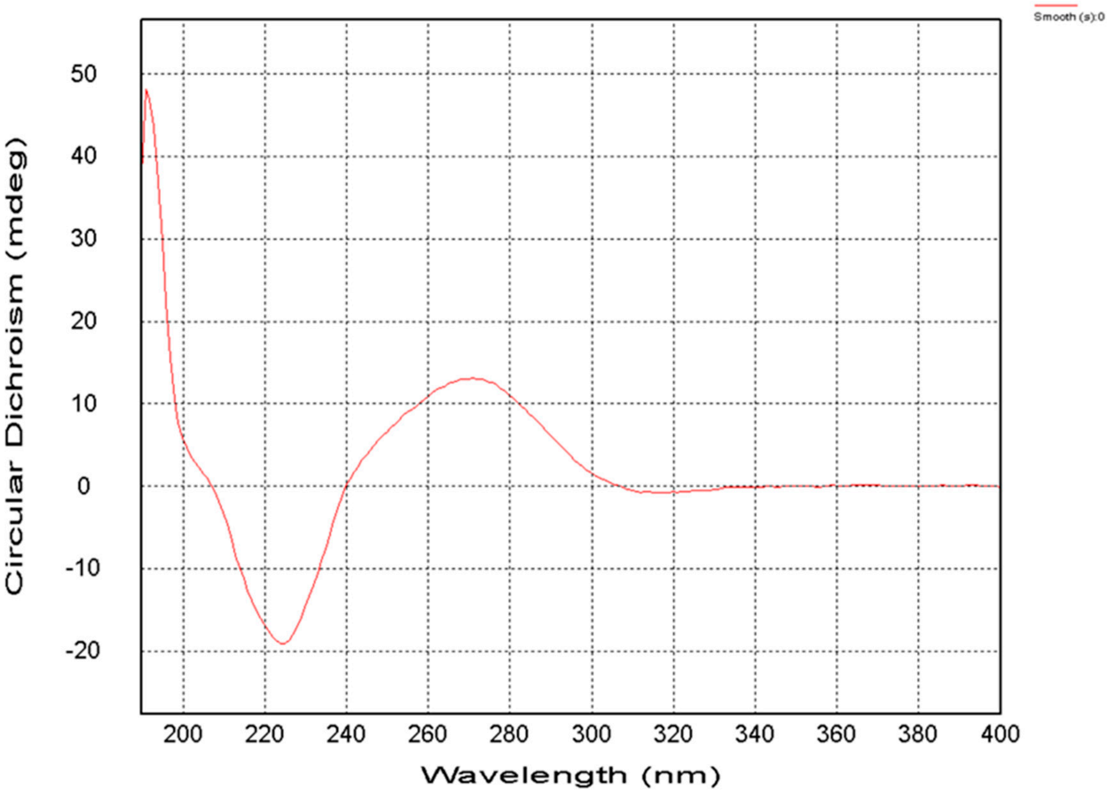


Figure S34. CD spectrum of compound **4** in MeOH

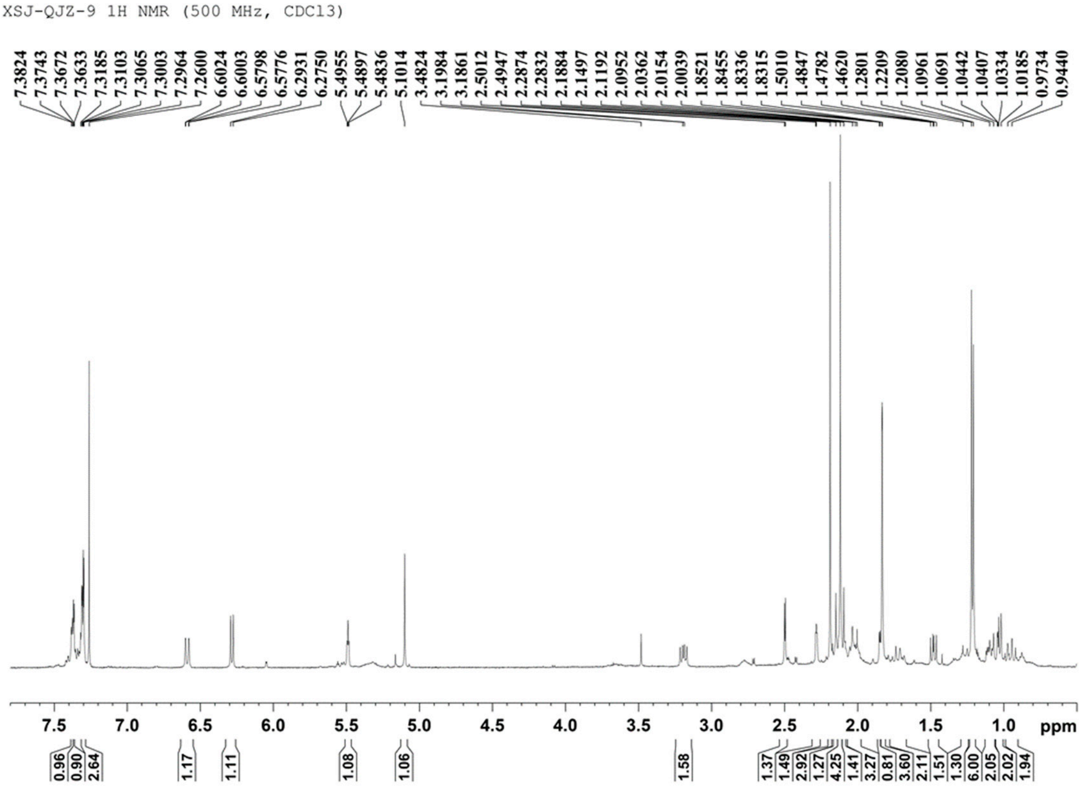


Figure S35. $^{1}H$ NMR spectrum of compound **4** in $CDCl_3$

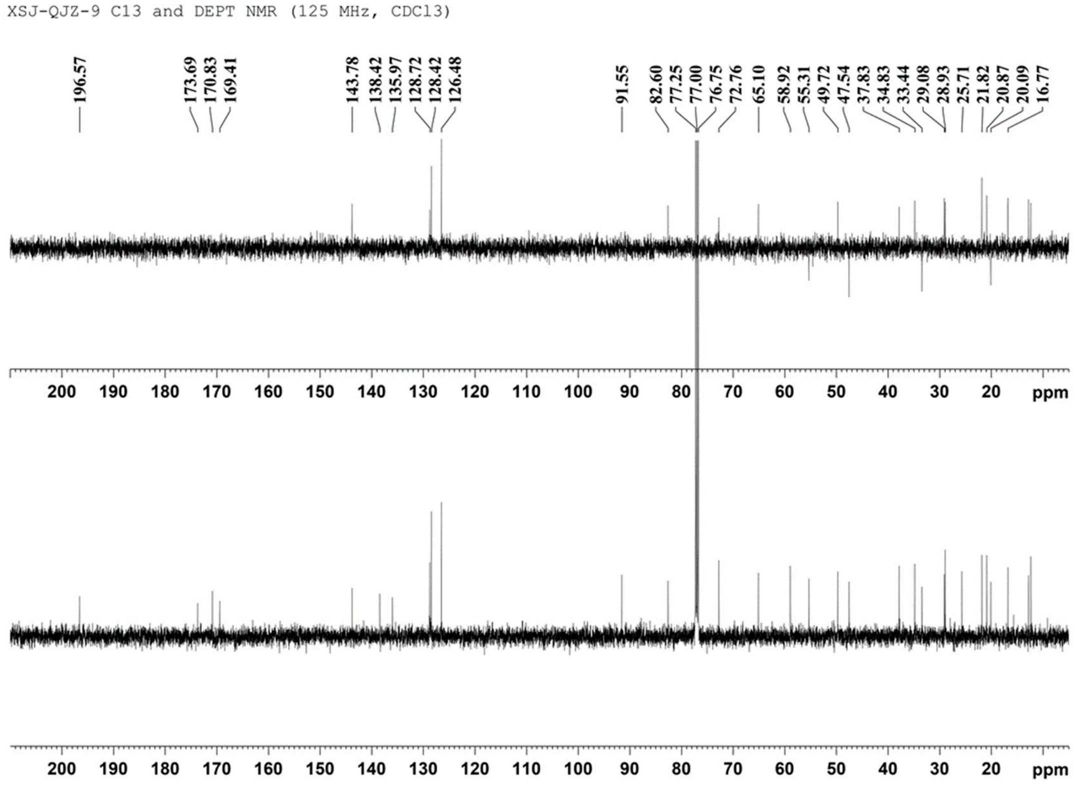


Figure S36. $^{13}C$ NMR and DEPT spectrum of compound **4** in $CDCl_3$

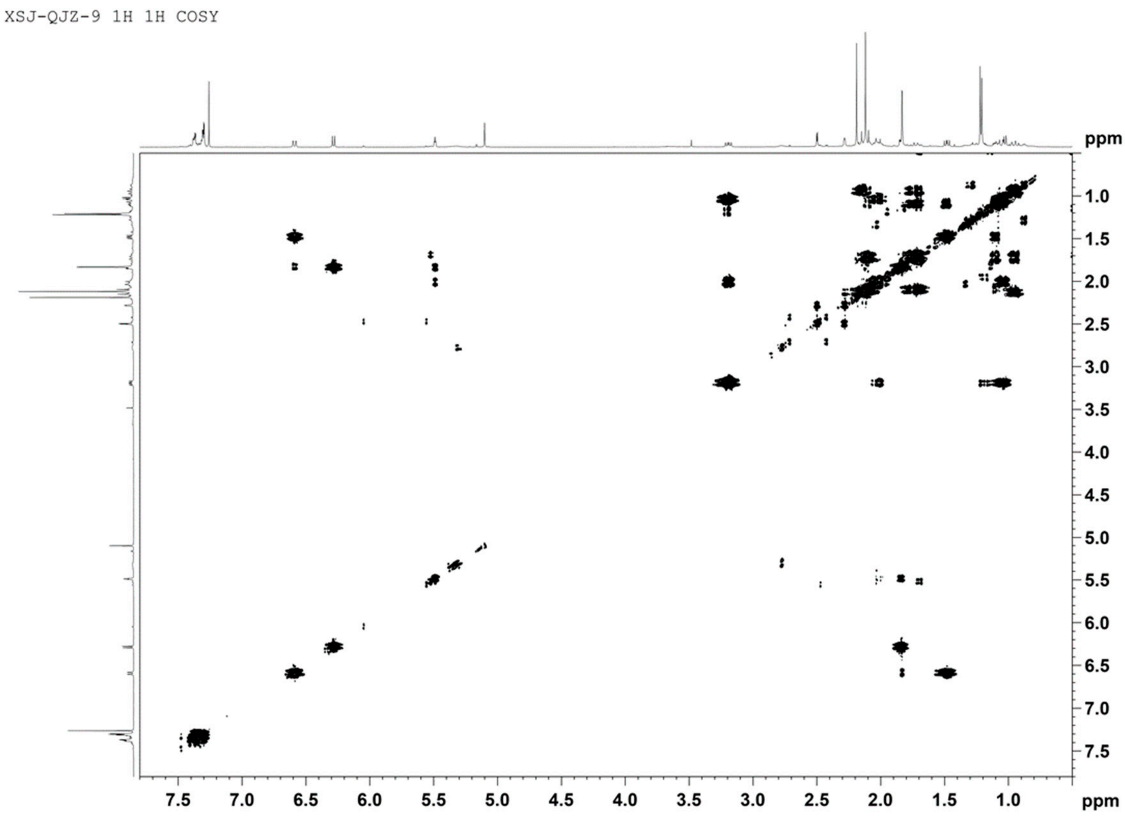


Figure S37. $^{1}H$-$^{1}H$ COSY spectrum of compound **4** in $CDCl_3$

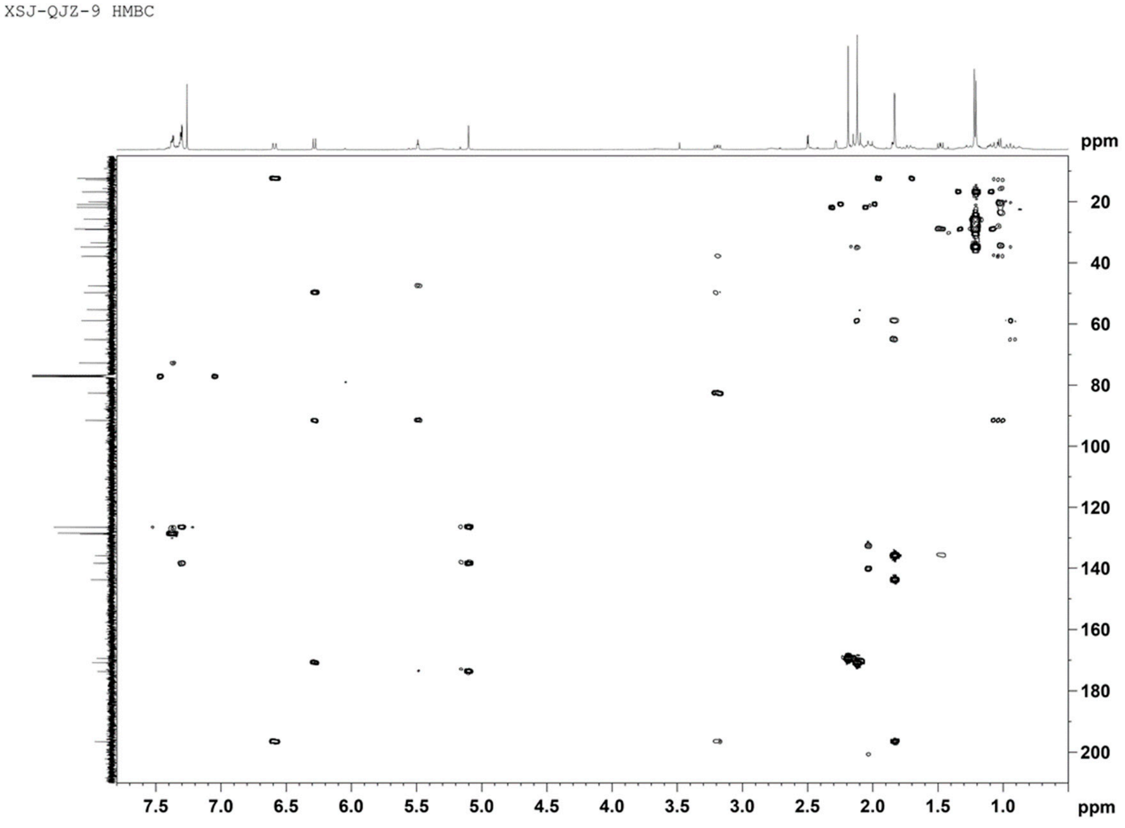


Figure S38. HMBC spectrum of compound **4** in $CDCl_3$

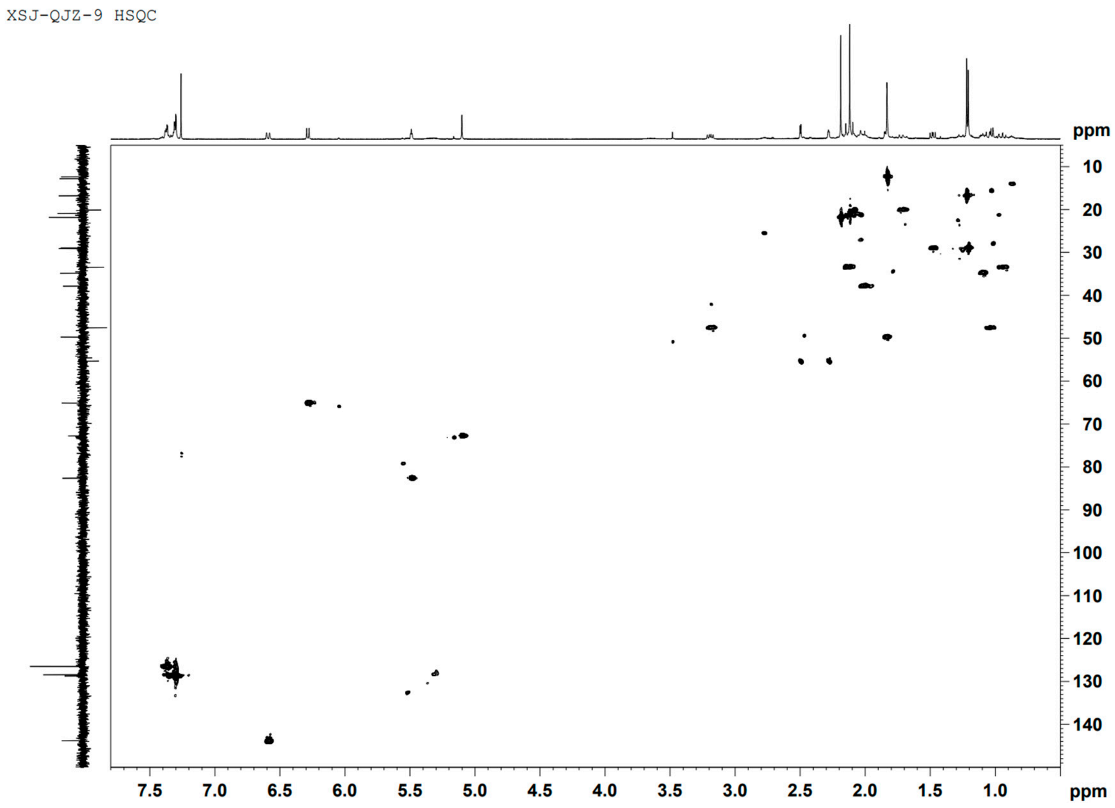


Figure S39. HSQC spectrum of compound **4** in $CDCl_3$

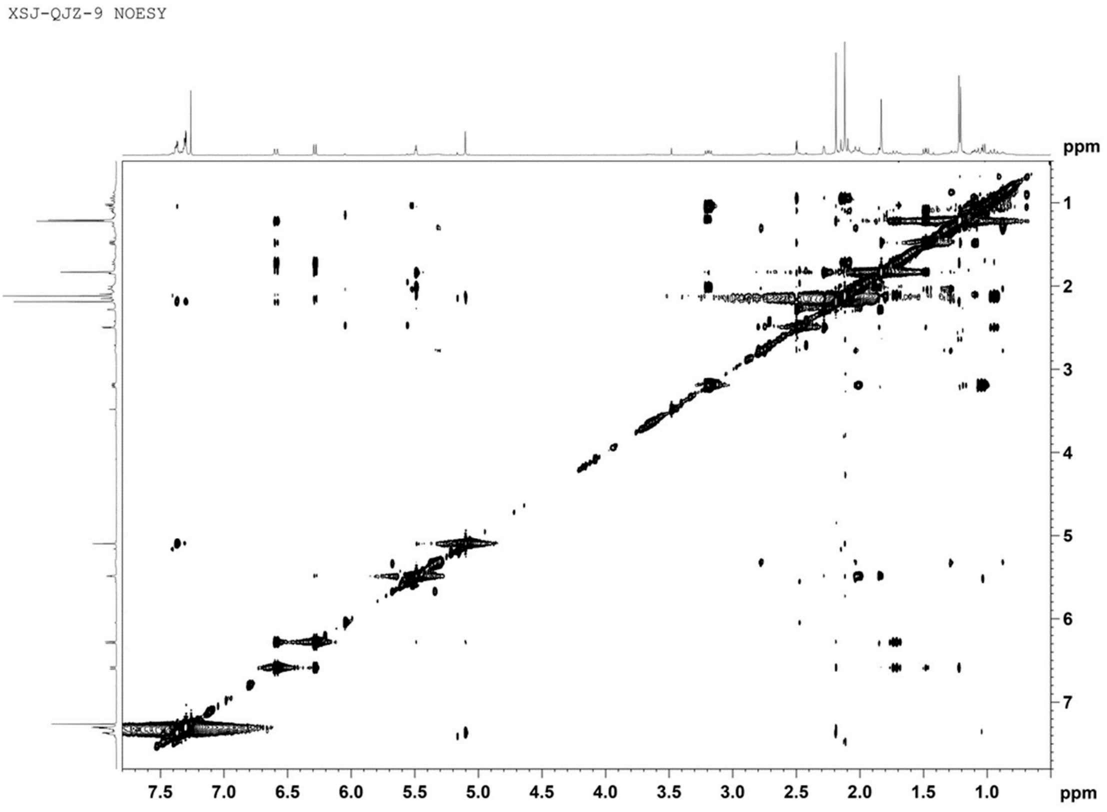


Figure S40. NOESY spectrum of compound **4** in $CDCl_3$

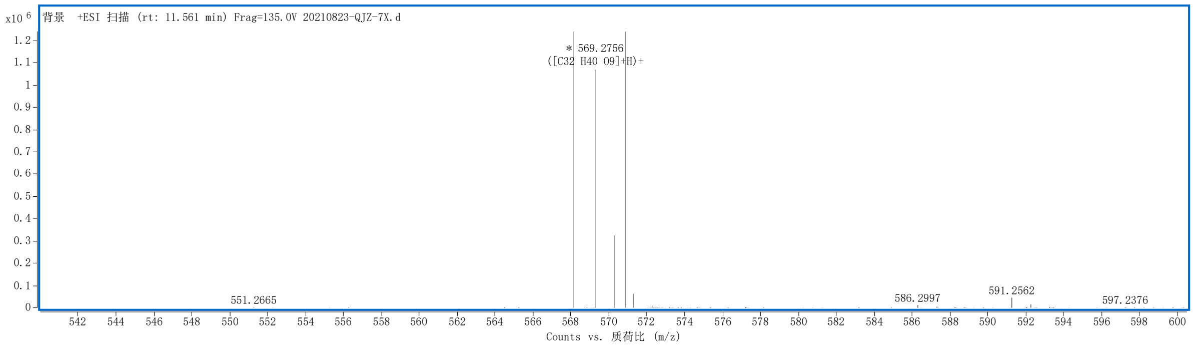


| Best | Formula(M) | Ion Formula | Tgt (m/z) | m/z | Diff (ppm) |
|---|---|---|---|---|---|
| TRUE | $C_{32}H_{40}O_9$ | $C_{32}H_{41}O_9$ | 569.2745 | 569.2756 | 1.93 |

Figure S41. HR-ESI-MS spectrum of compound **5**

**Rudolph Research Analytical**

Monday, 10/11/2021

This sample was measured on an Autopol VI, serial number 90079,
manufactured by Rudolph Research Analytical,Hackettstown,NJ.

LotID : QJZ-7
Set Temperature : 20.0
Temp Corr : OFF

| n | Average | Std.Dev. | Maximum | Minimum |
|---|---|---|---|---|
| 6 | 112.654 | 0.4365 | 112.963 | 112.037 |

| S.No | Sample ID | Time | Result | Scale | OR ° Arc | WLG | Lg.mm | Conc. | Temp. | Comment |
|---|---|---|---|---|---|---|---|---|---|---|
| 1 | QJZ-7 | 10:58:17 AM | 112.037 | SR | 0.121 | 589 | 100.00 | 0.108 | 20.1 | |
| 2 | QJZ-7 | 10:58:25 AM | 112.037 | SR | 0.121 | 589 | 100.00 | 0.108 | 20.1 | |
| 3 | QJZ-7 | 10:58:32 AM | 112.963 | SR | 0.122 | 589 | 100.00 | 0.108 | 20.1 | |
| 4 | QJZ-7 | 10:58:40 AM | 112.963 | SR | 0.122 | 589 | 100.00 | 0.108 | 20.0 | |
| 5 | QJZ-7 | 10:58:47 AM | 112.963 | SR | 0.122 | 589 | 100.00 | 0.108 | 20.0 | |
| 6 | QJZ-7 | 10:58:54 AM | 112.963 | SR | 0.122 | 589 | 100.00 | 0.108 | 20.0 | |

________________________
Signature

Figure S42. OR value of compound **5** in MeOH

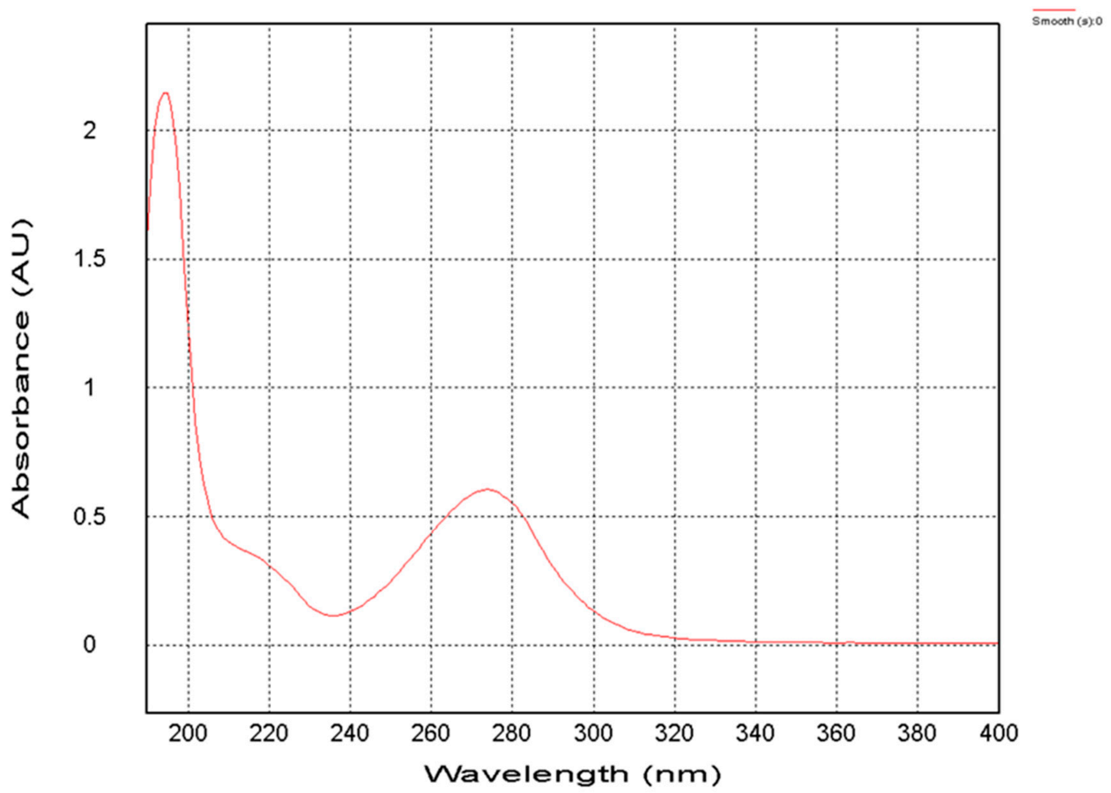


Figure S43. UV spectrum of compound **5** in MeOH

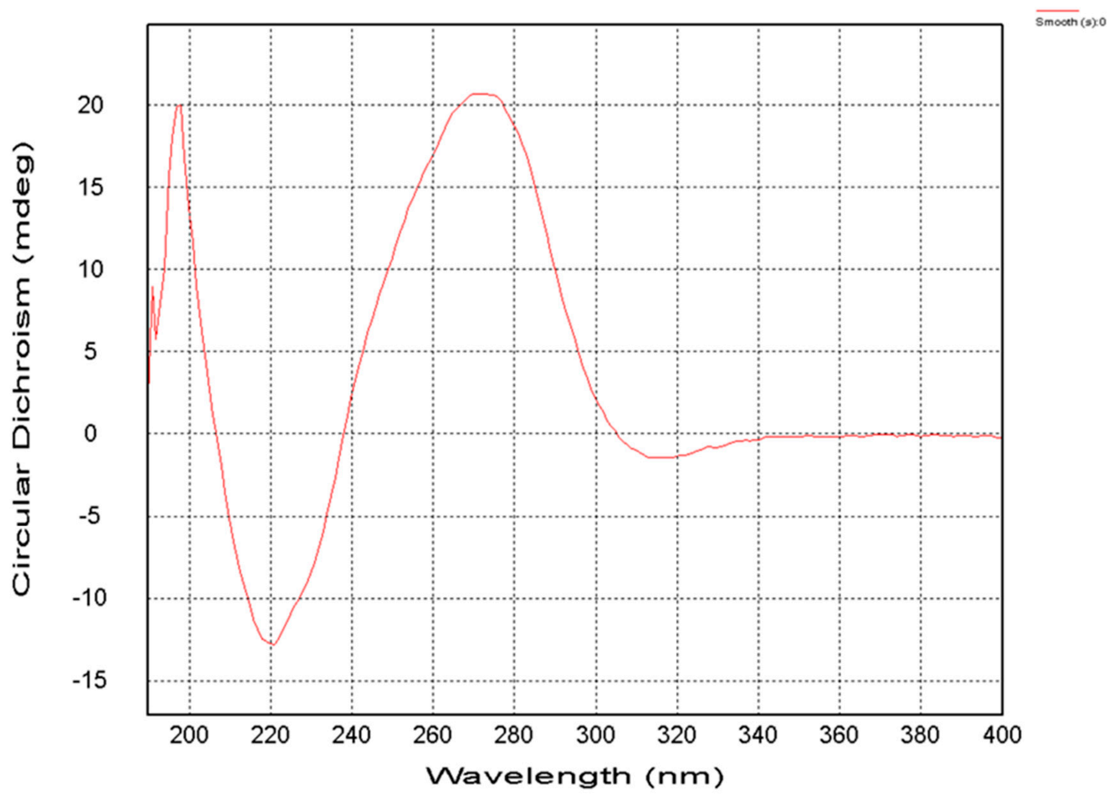


Figure S44. CD spectrum of compound **5** in MeOH

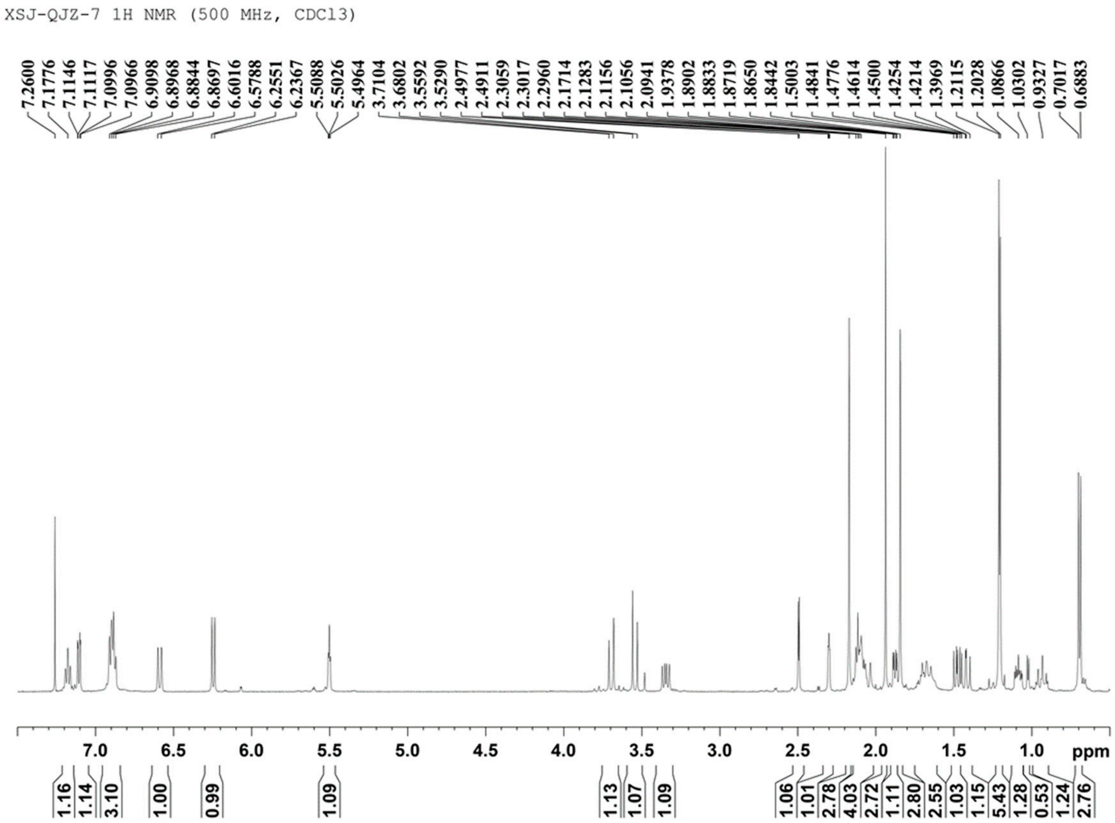


Figure S45. $^{1}$H NMR spectrum of compound **5** in $CDCl_3$

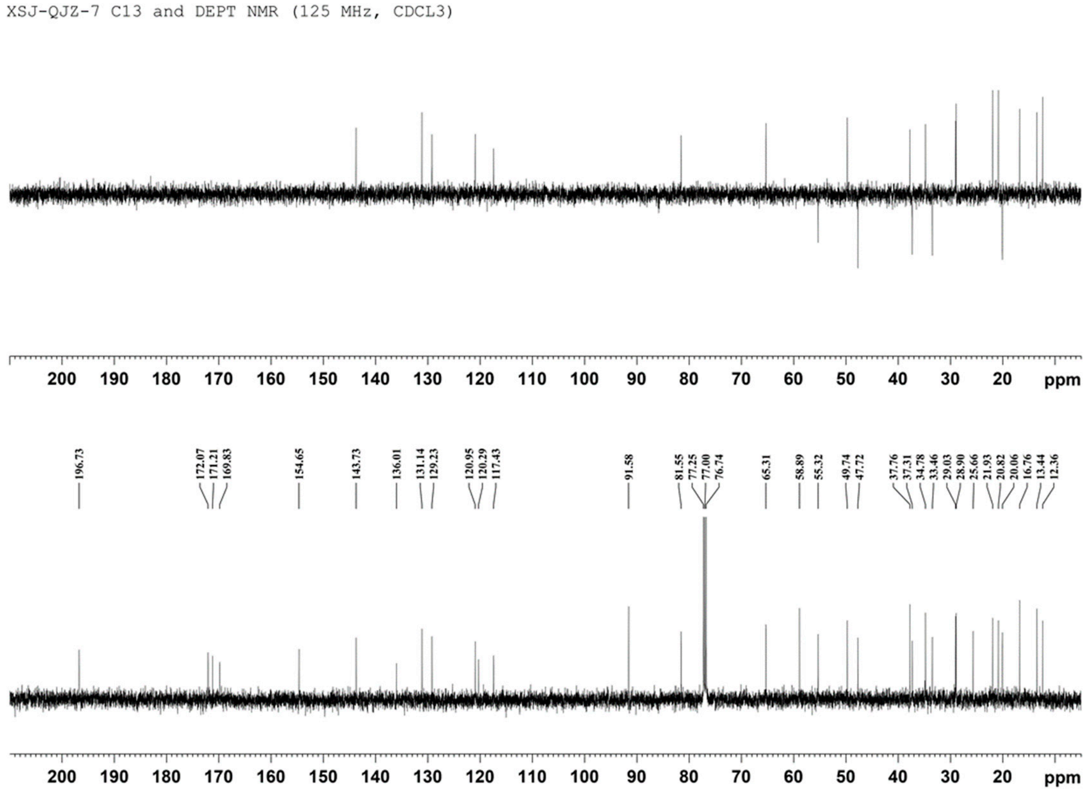


Figure S46. $^{13}$C NMR and DEPT spectrum of compound **5** in $CDCl_3$

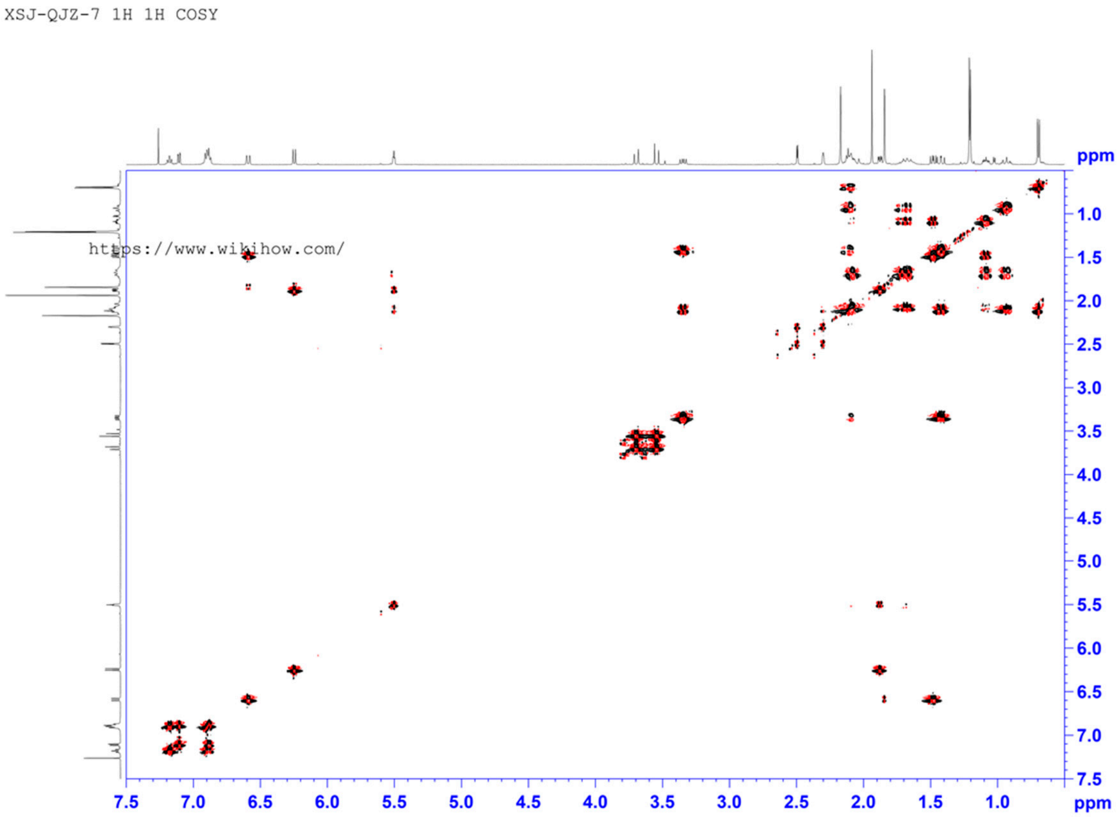


Figure S47. $^{1}H$-$^{1}H$ COSY spectrum of compound **5** in $CDCl_3$

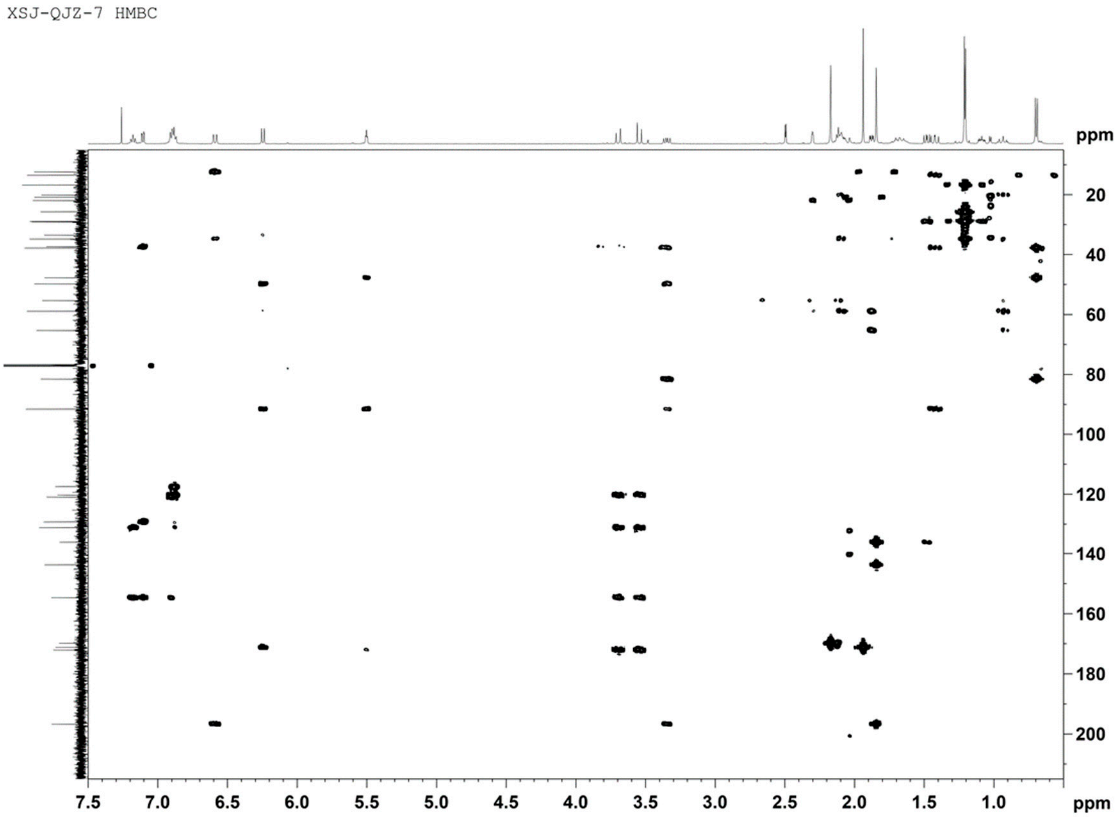


Figure S48. HMBC spectrum of compound **5** in $CDCl_3$

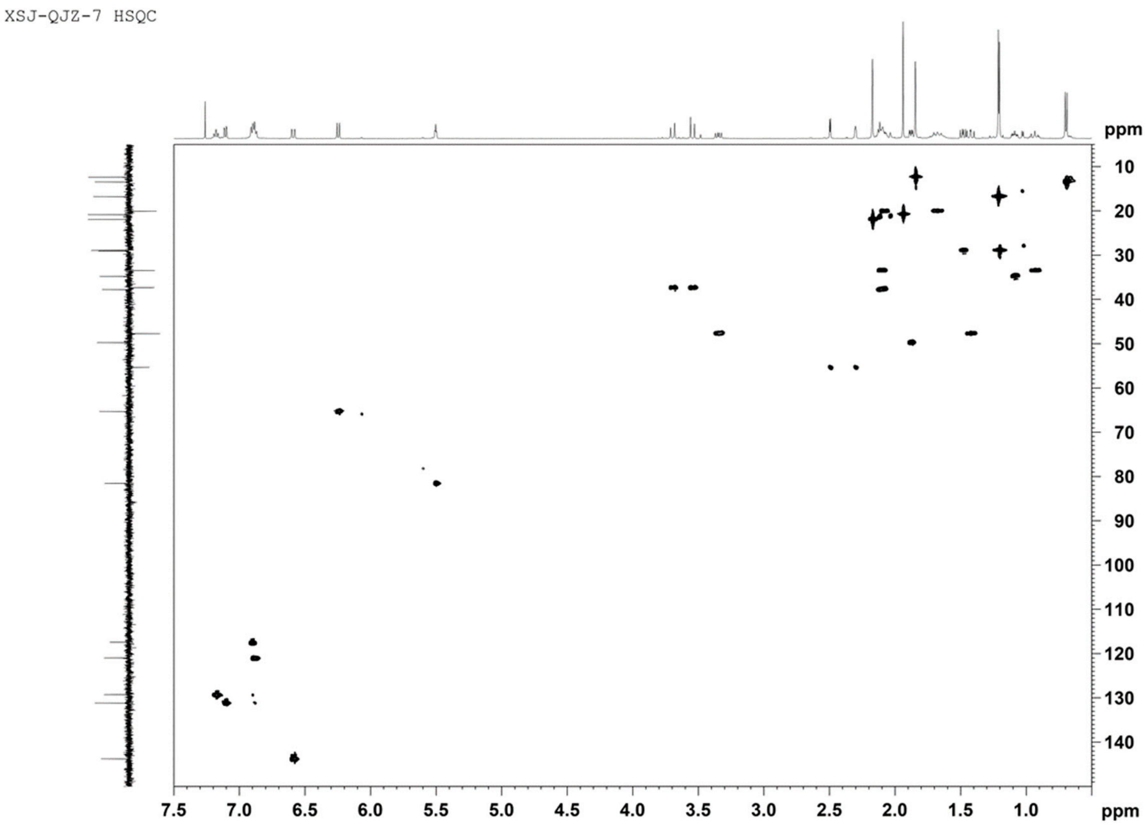


Figure S49. HSQC spectrum of compound **5** in $CDCl_3$

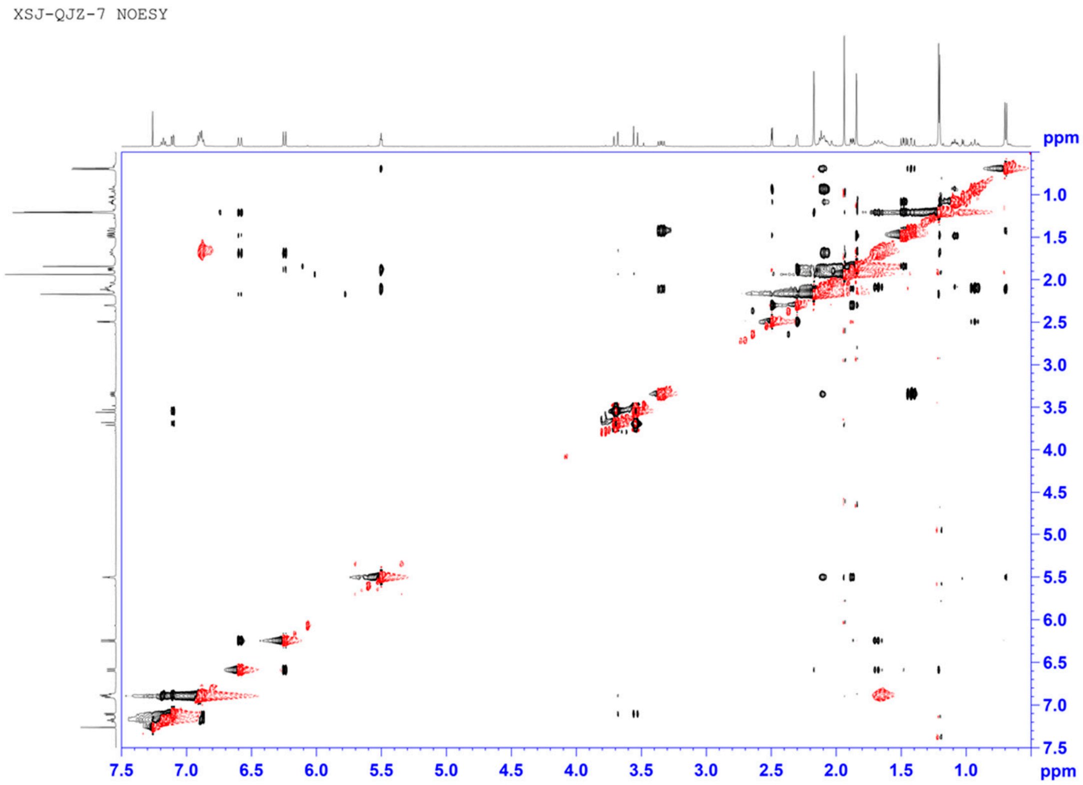


Figure S50. NOESY spectrum of compound **5** in $CDCl_3$

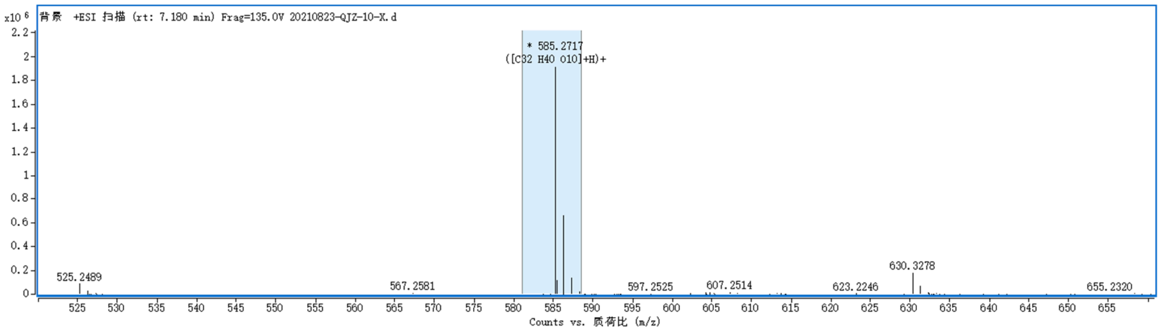


| Best | Formula(M) | Ion Formula | Tgt (m/z) | m/z | Diff (ppm) |
|---|---|---|---|---|---|
| TRUE | $C_{32}H_{40}O_{10}$ | $C_{32}H_{41}O_{10}$ | 585.2694 | 585.2717 | 3.93 |

Figure S51. HR-ESI-MS spectrum of compound **6**

**Rudolph Research Analytical**

Monday, 10/11/2021

This sample was measured on an Autopol VI, serial number 90079, manufactured by Rudolph Research Analytical,Hackettstown,NJ.

LotID : QJZ-10
Set Temperature : 20.0
Temp Corr : OFF

| n | Average | Std.Dev. | Maximum | Minimum |
|---|---|---|---|---|
| 6 | 87.143 | 0.4760 | 87.619 | 86.667 |

| S.No | Sample ID | Time | Result | Scale | OR °Arc | WLG | Lg.mm | Conc. | Temp. | Comment |
|---|---|---|---|---|---|---|---|---|---|---|
| 1 | QJZ-10 | 10:17:24 AM | 86.667 | SR | 0.091 | 589 | 100.00 | 0.105 | 20.3 | |
| 2 | QJZ-10 | 10:17:32 AM | 86.667 | SR | 0.091 | 589 | 100.00 | 0.105 | 20.2 | |
| 3 | QJZ-10 | 10:17:39 AM | 87.619 | SR | 0.092 | 589 | 100.00 | 0.105 | 20.2 | |
| 4 | QJZ-10 | 10:17:47 AM | 86.667 | SR | 0.091 | 589 | 100.00 | 0.105 | 20.2 | |
| 5 | QJZ-10 | 10:17:54 AM | 87.619 | SR | 0.092 | 589 | 100.00 | 0.105 | 20.1 | |
| 6 | QJZ-10 | 10:18:02 AM | 87.619 | SR | 0.092 | 589 | 100.00 | 0.105 | 20.1 | |

Signature

Figure S52.OR value of compound **6** in MeOH

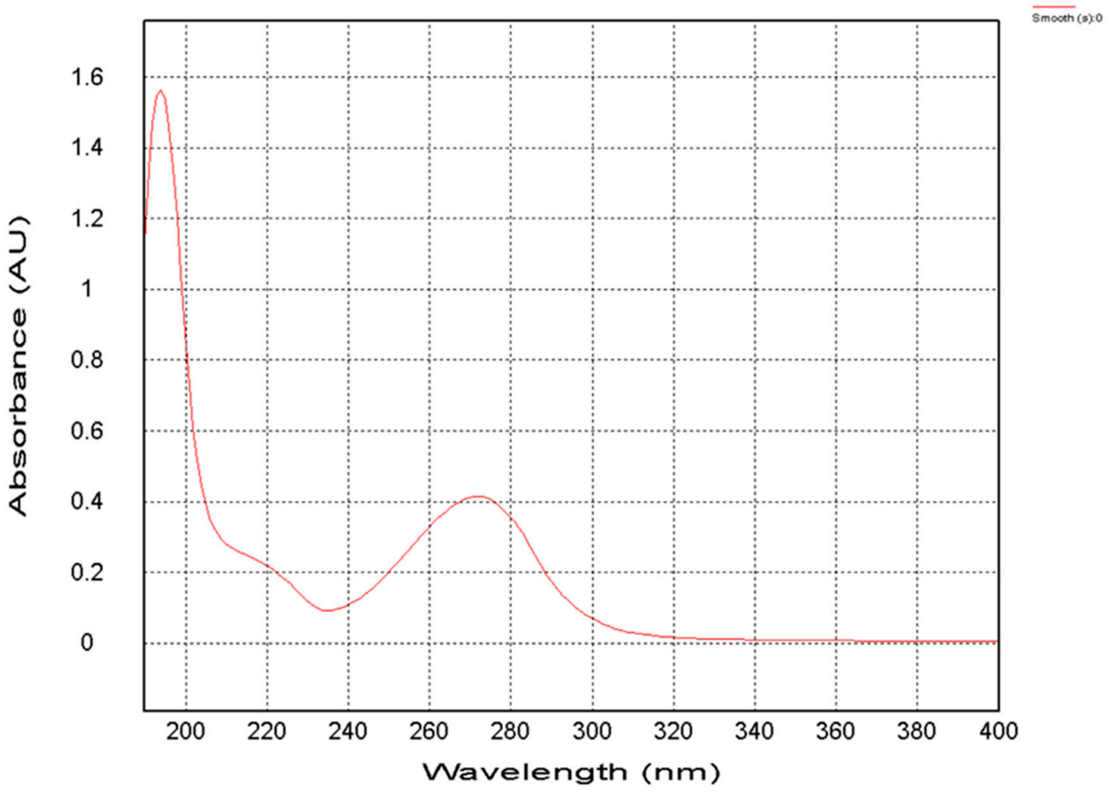


Figure S53. UV spectrum of compound **6** in MeOH

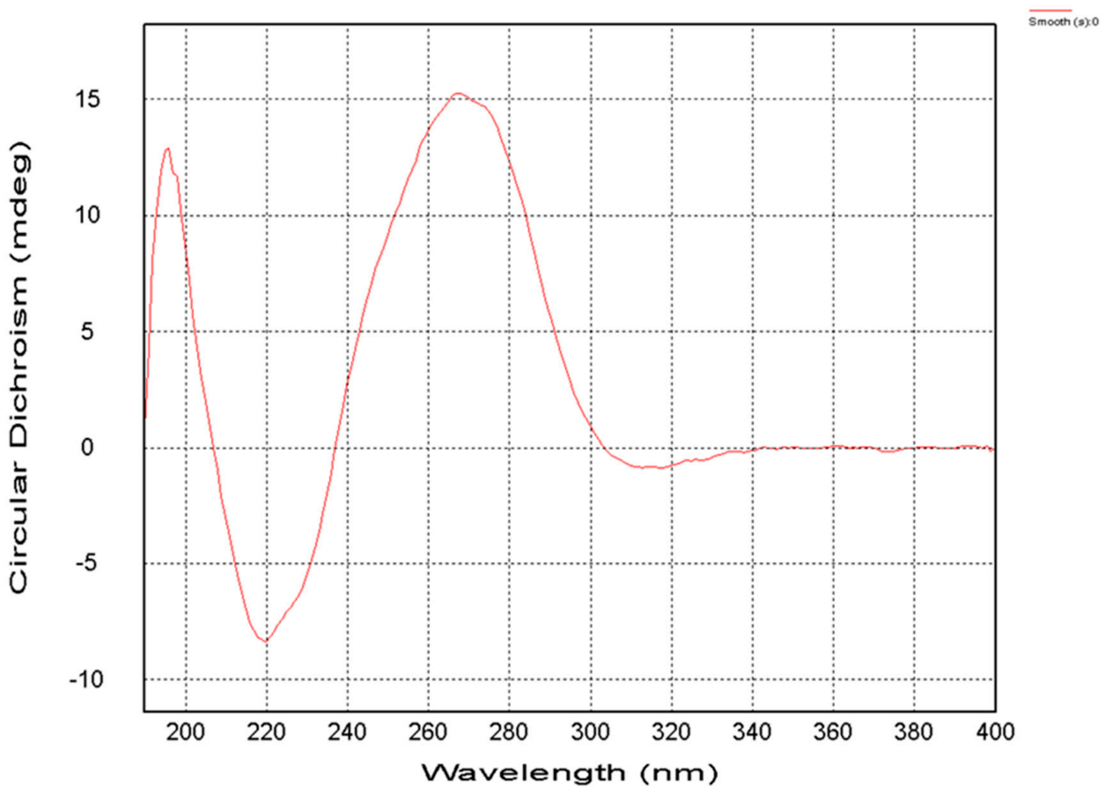


Figure S54. CD spectrum of compound **6** in MeOH

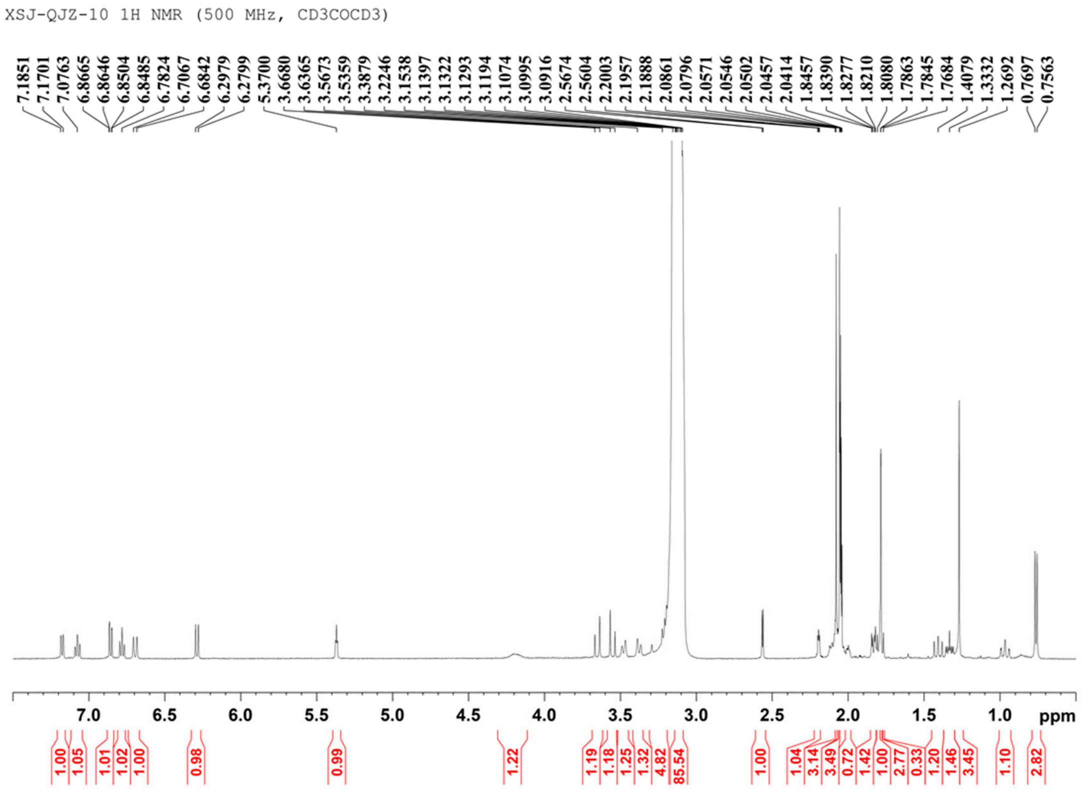


Figure S55. $^{1}H$ NMR spectrum of compound **6** in $C_3D_6O$

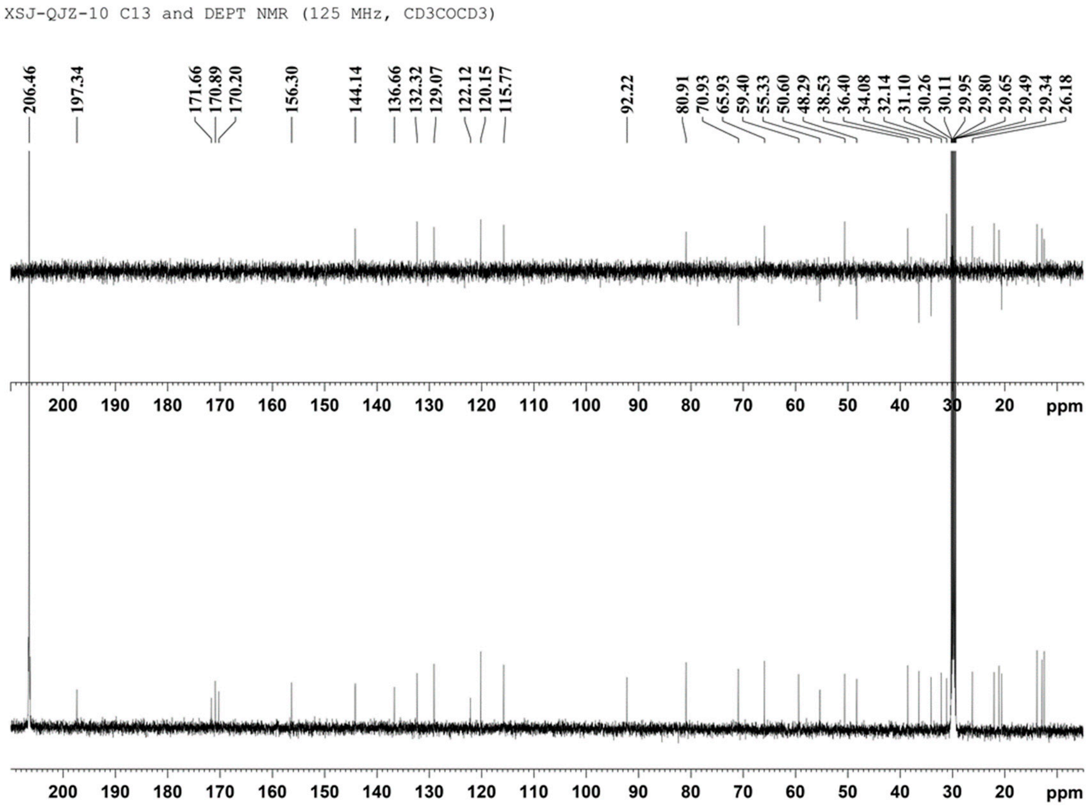


Figure S56. $^{13}C$ NMR and DEPT spectrum of compound **6** in $C_3D_6O$

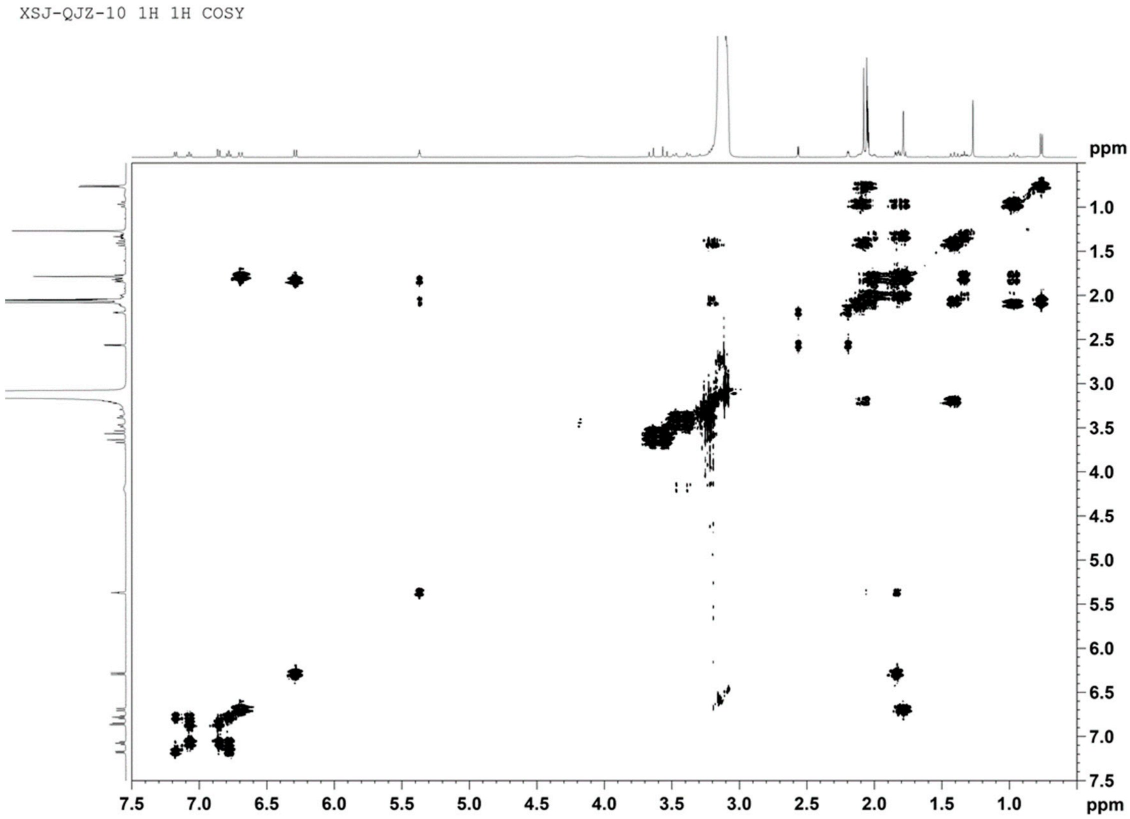


Figure S57. $^{1}H$-$^{1}H$ NMR spectrum of compound **6** in $C_3D_6O$

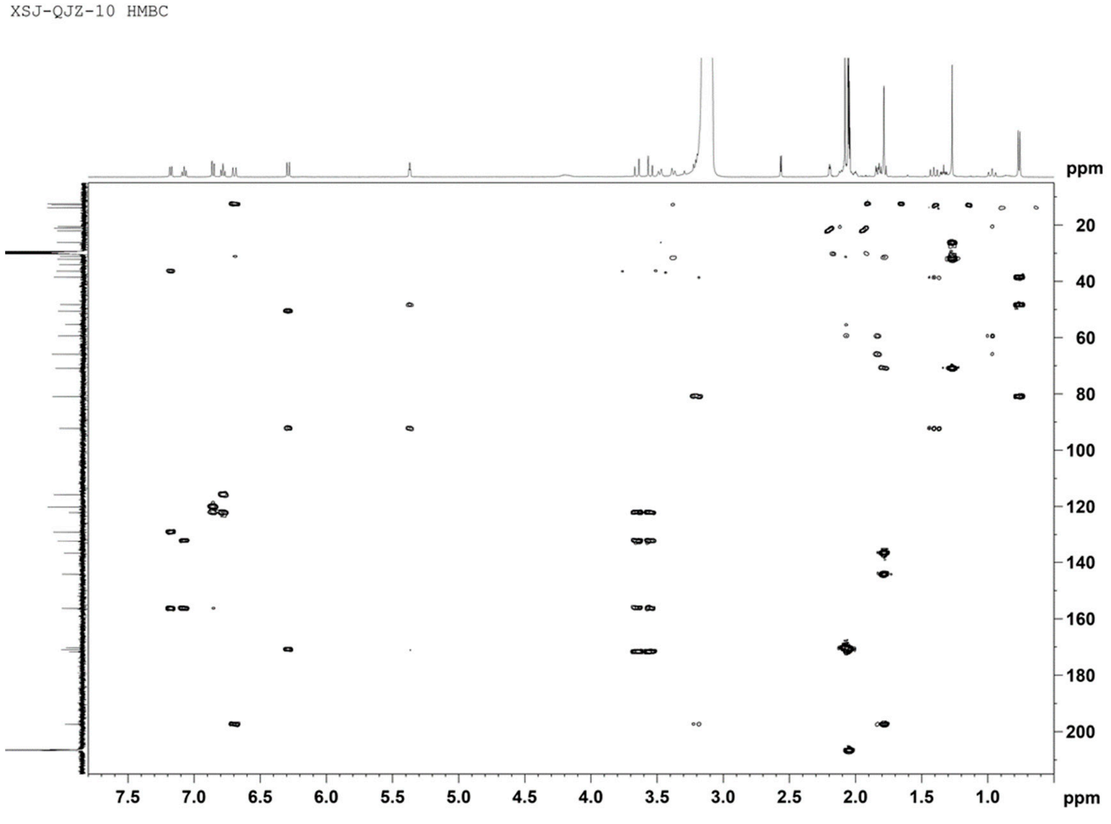


Figure S58. HMBC spectrum of compound **6** in $C_3D_6O$

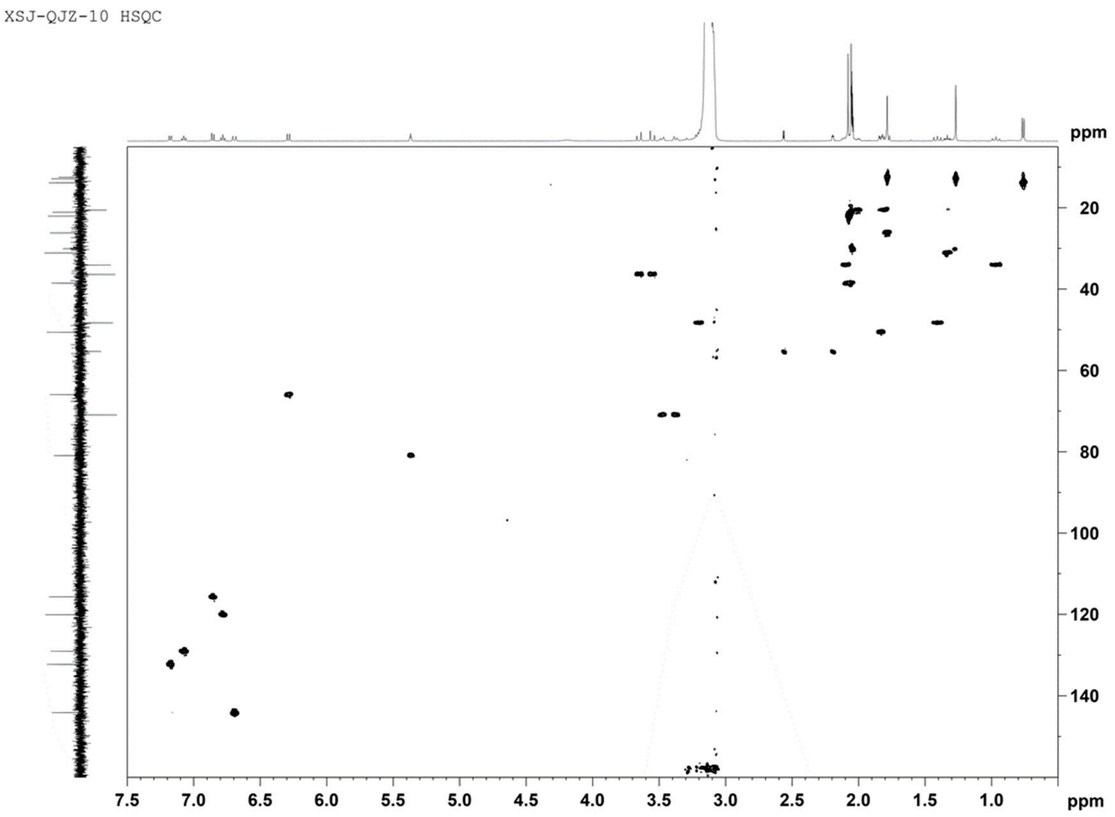


Figure S59. HSQC spectrum of compound **6** in $C_3D_6O$

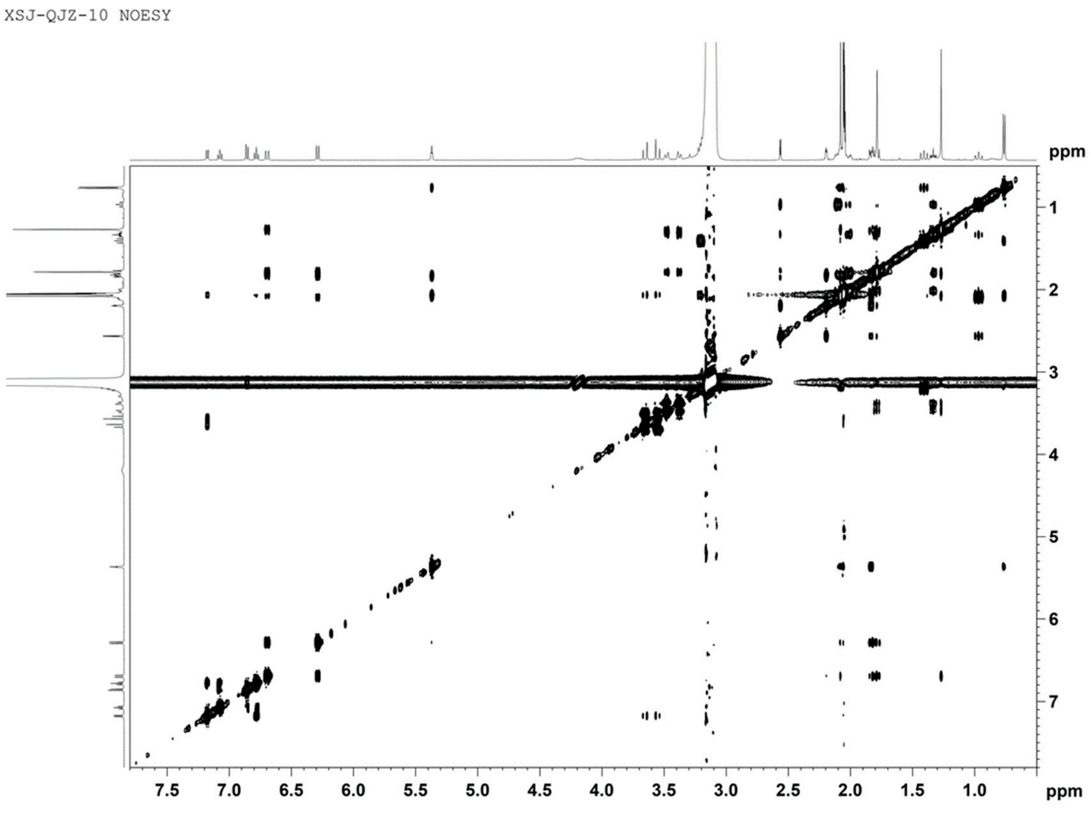


Figure S60. NOESY spectrum of compound **6** in $C_3D_6O$

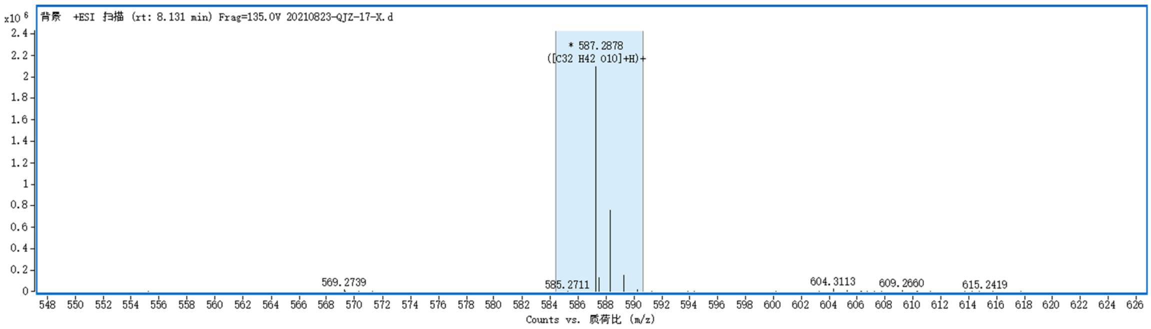


| Best | Formula(M) | Ion Formula | Tgt (m/z) | m/z | Diff (ppm) |
|---|---|---|---|---|---|
| TRUE | $C_{32}H_{42}O_{10}$ | $C_{32}H_{43}O_{10}$ | 587.2851 | 587.2878 | 4.59 |

Figure S61. HR-ESI-MS spectrum of compound **7**

**Rudolph Research Analytical**

Saturday, 10/09/2021

This sample was measured on an Autopol VI, serial number 90079, manufactured by Rudolph Research Analytical,Hackettstown,NJ.

LotID : QJZ-17
Set Temperature : 20.0
Temp Corr : OFF

| n | Average | Std.Dev. | Maximum | Minimum |
|---|---|---|---|---|
| 6 | 60.000 | 0.0000 | 60.000 | 60.000 |

| S.No | Sample ID | Time | Result | Scale | OR °Arc | WLG | Lg.mm | Conc. | Temp. | Comment |
|---|---|---|---|---|---|---|---|---|---|---|
| 1 | QJZ-17 | 04:36:50 PM | 60.000 | SR | 0.102 | 589 | 100.00 | 0.170 | 19.7 | |
| 2 | QJZ-17 | 04:36:57 PM | 60.000 | SR | 0.102 | 589 | 100.00 | 0.170 | 19.7 | |
| 3 | QJZ-17 | 04:37:05 PM | 60.000 | SR | 0.102 | 589 | 100.00 | 0.170 | 19.7 | |
| 4 | QJZ-17 | 04:37:12 PM | 60.000 | SR | 0.102 | 589 | 100.00 | 0.170 | 19.7 | |
| 5 | QJZ-17 | 04:37:20 PM | 60.000 | SR | 0.102 | 589 | 100.00 | 0.170 | 19.8 | |
| 6 | QJZ-17 | 04:37:27 PM | 60.000 | SR | 0.102 | 589 | 100.00 | 0.170 | 19.8 | |

Signature

Figure S62. OR value of compound **7** in MeOH

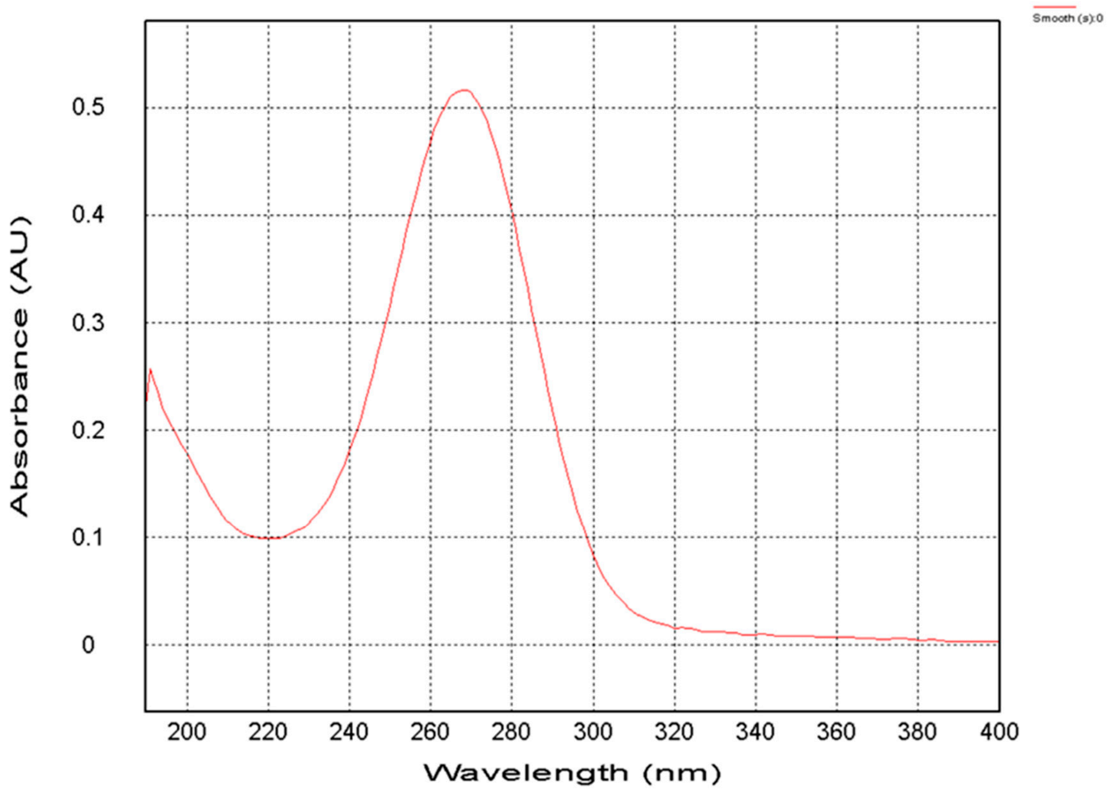


Figure S63. UV spectrum of compound **7** in MeOH

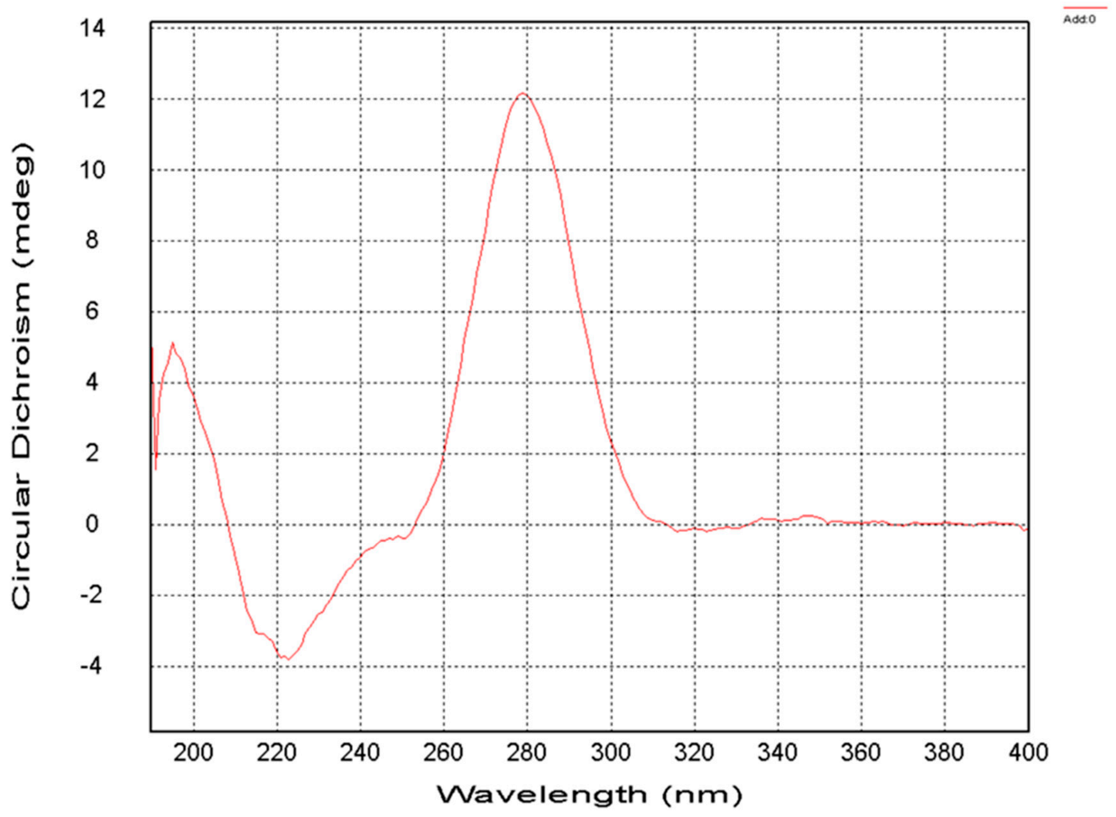


Figure S64. CD spectrum of compound **7** in MeOH

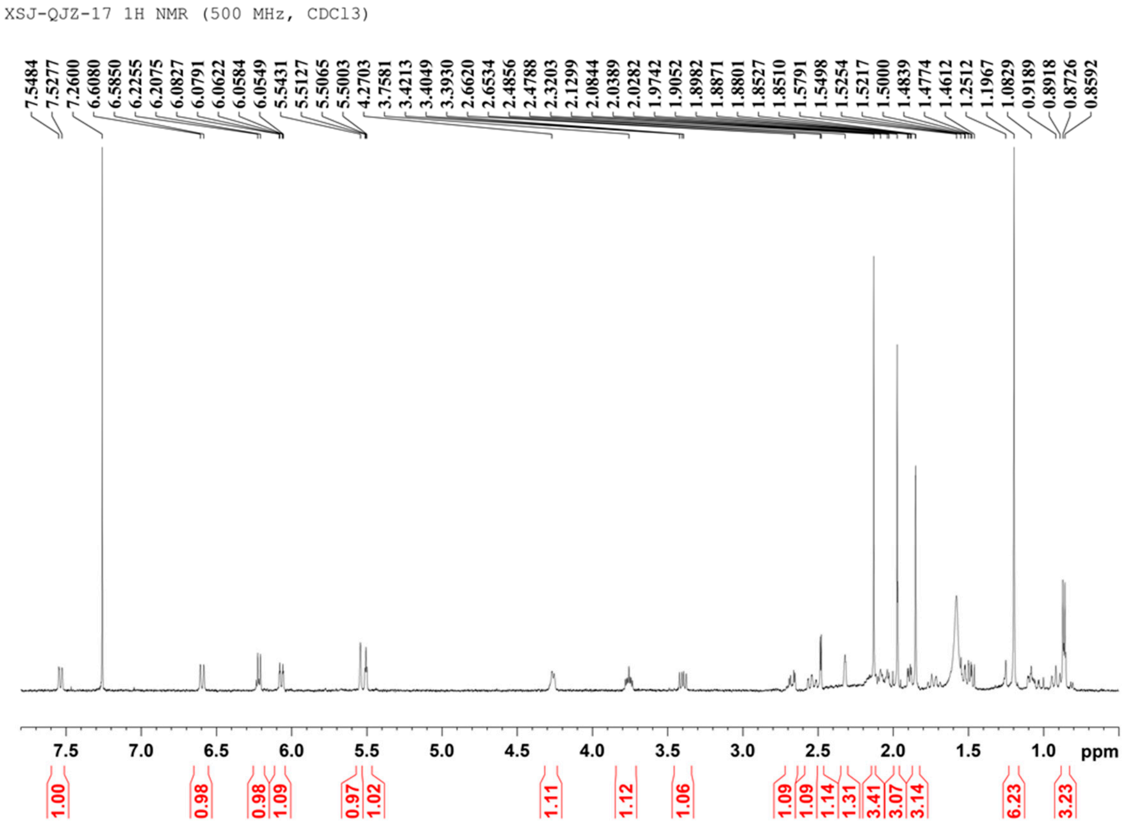


Figure S65. $^{1}$H NMR spectrum of compound **7** in $CDCl_3$

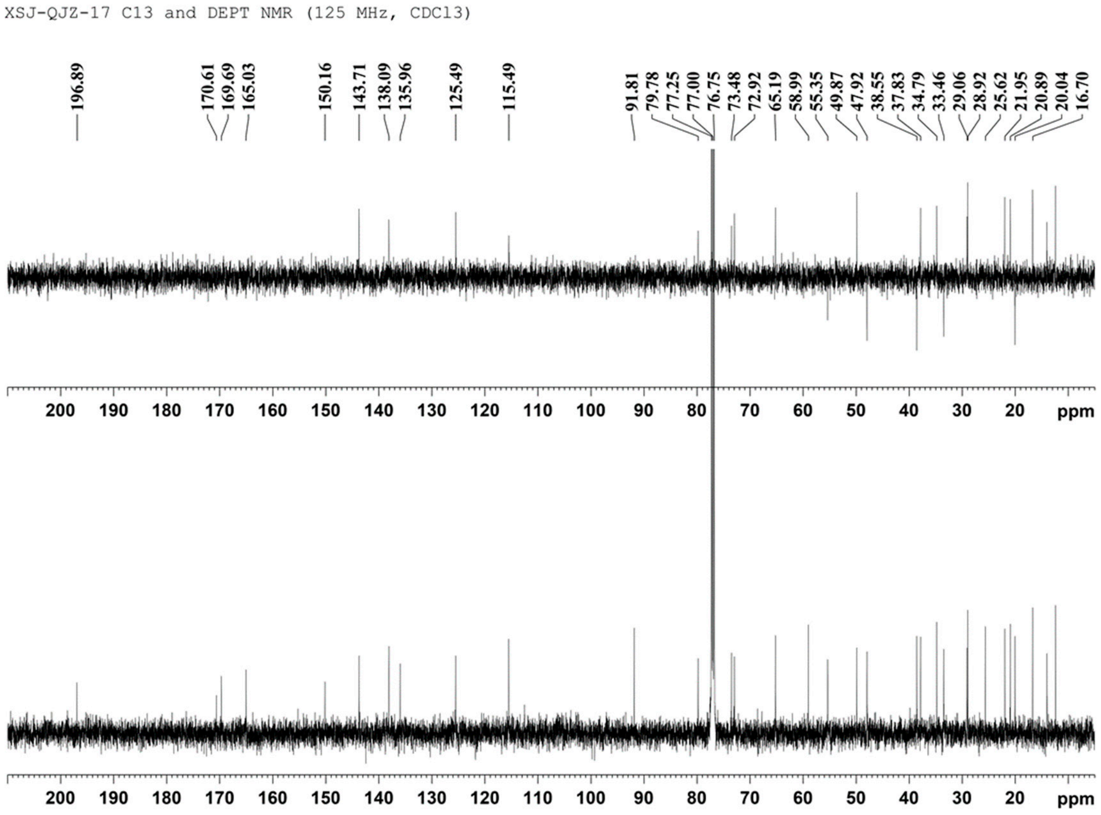


Figure S66. $^{13}$C NMR and DEPT spectrum of compound **7** in $CDCl_3$

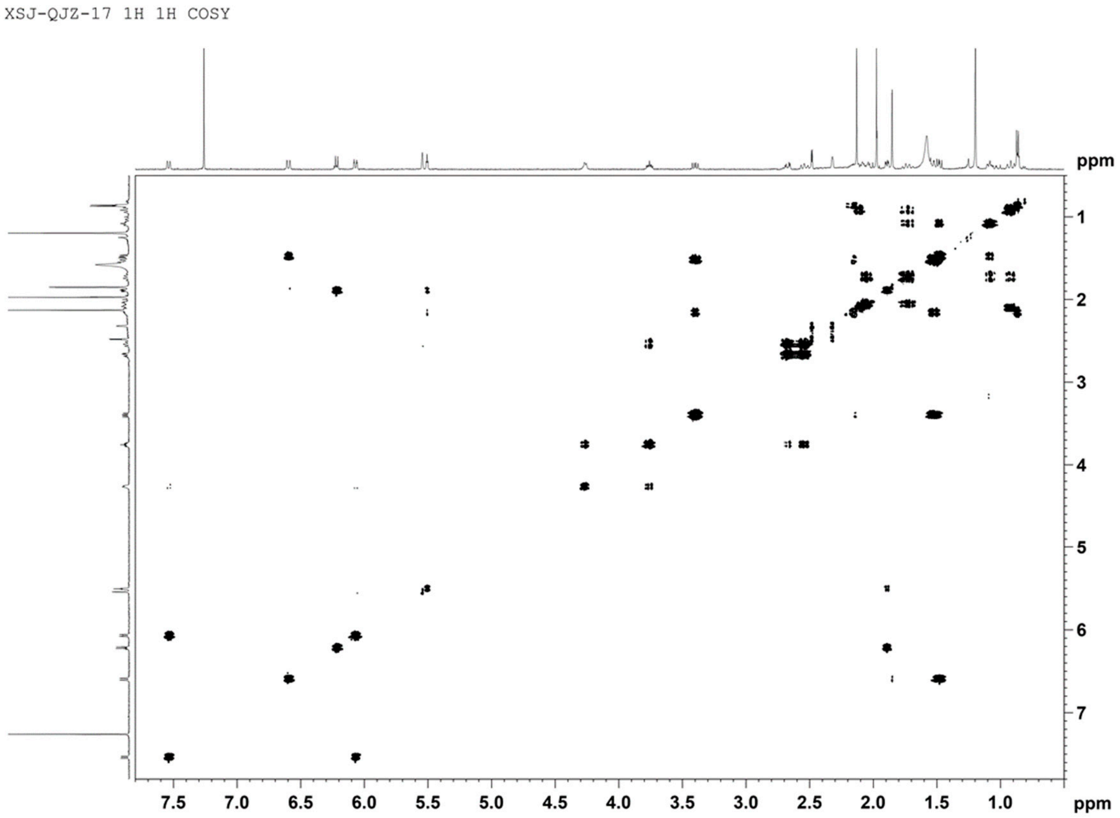


Figure S67. $^{1}$H-$^{1}$H NMR spectrum of compound **7** in $CDCl_3$

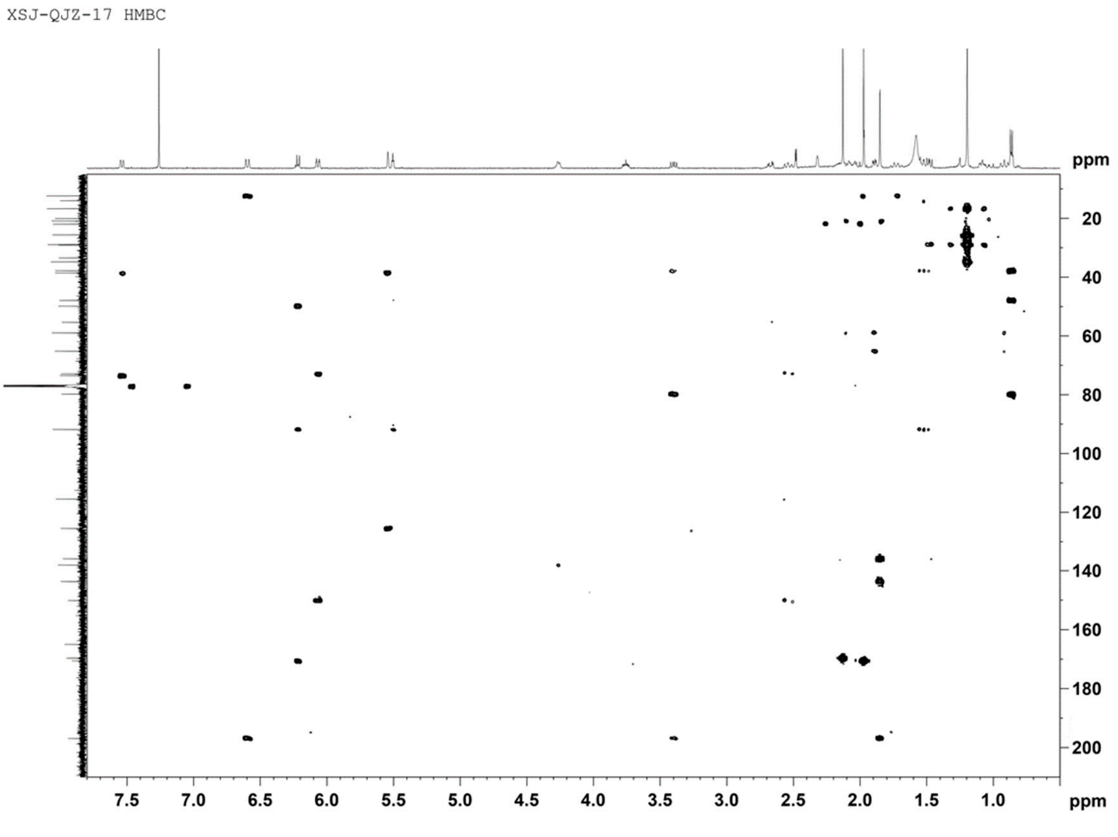


Figure S68. HMBC spectrum of compound **7** in $CDCl_3$

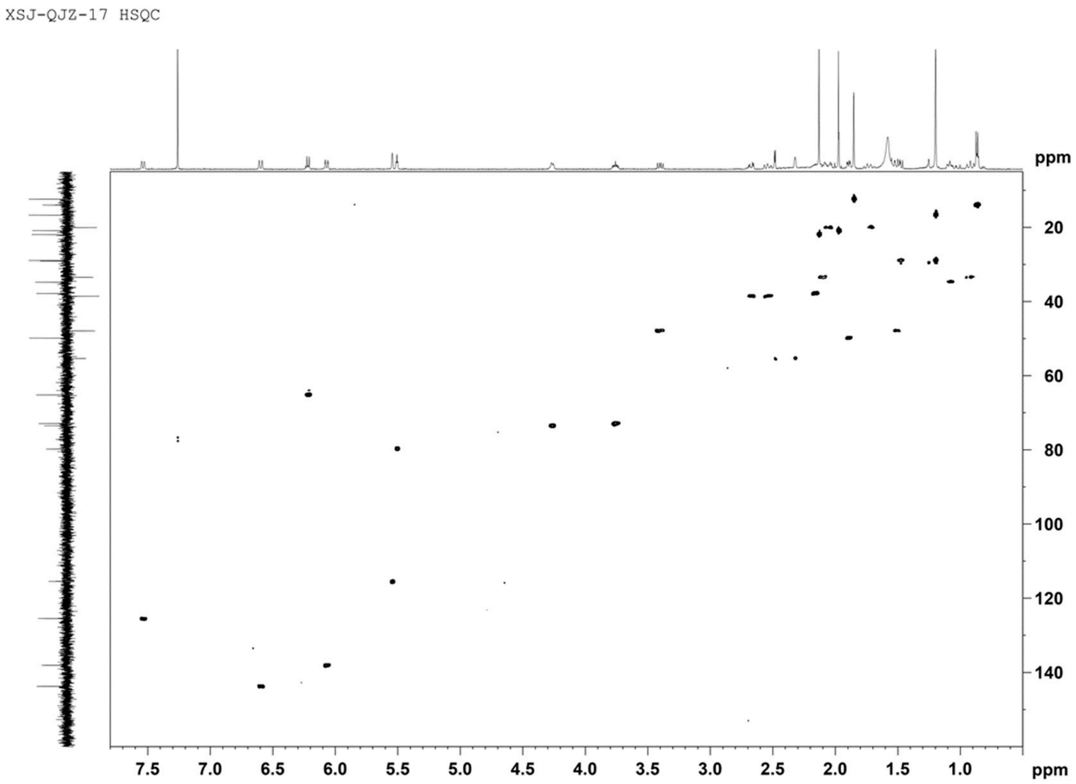


Figure S69. HSQC spectrum of compound **7** in $CDCl_3$

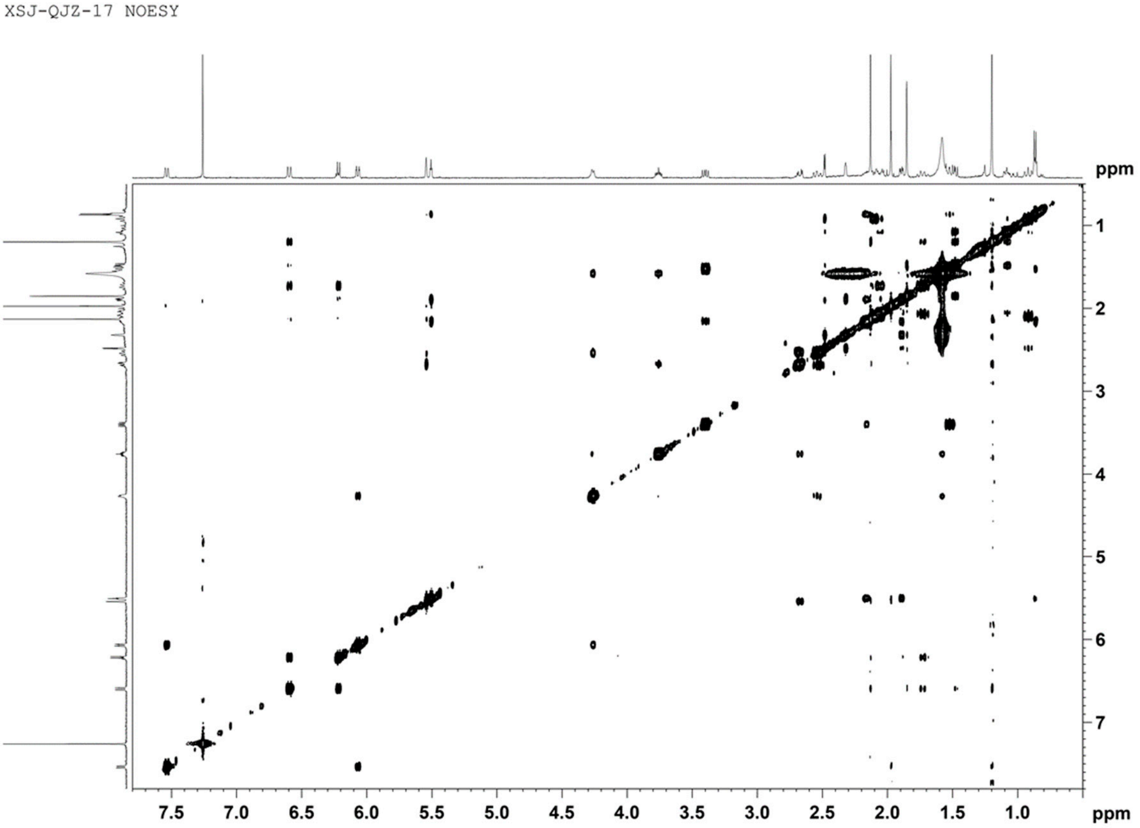


Figure S70. NOESY spectrum of compound **7** in $CDCl_3$

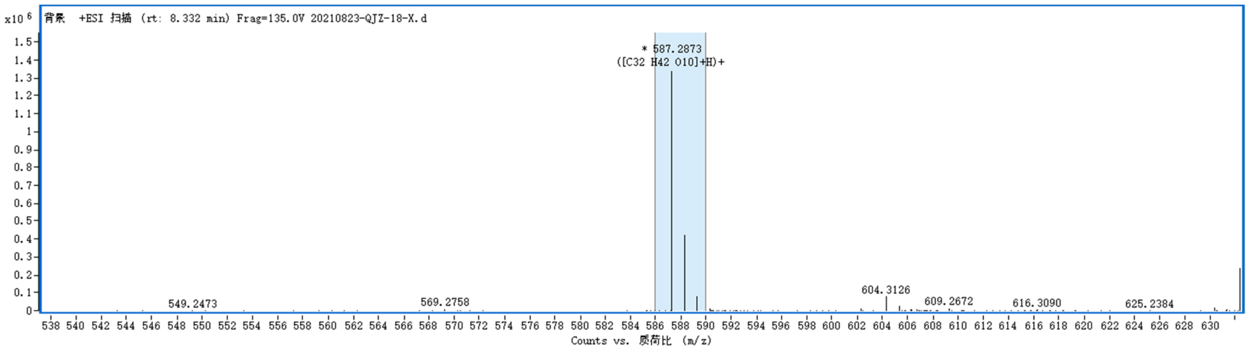


| Best | Formula(M) | Ion Formula | Tgt (m/z) | m/z | Diff (ppm) |
|---|---|---|---|---|---|
| TRUE | $C_{32}H_{42}O_{10}$ | $C_{32}H_{43}O_{10}$ | 587.2851 | 587.2873 | 3.75 |

Figure S71. HR-ESI-MS spectrum of compound **8**

**Rudolph Research Analytical**

Saturday, 10/09/2021

This sample was measured on an Autopol VI, serial number 90079, manufactured by Rudolph Research Analytical,Hackettstown,NJ.

LotID : QJZ-18
Set Temperature : 20.0
Temp Corr : OFF

| **n** | **Average** | **Std.Dev.** | **Maximum** | **Minimum** |
|---|---|---|---|---|
| 6 | 90.833 | 0.0000 | 90.833 | 90.833 |

| **S.No** | **Sample ID** | **Time** | **Result** | **Scale** | **OR °Arc** | **WLG** | **Lg.mm** | **Conc.** | **Temp.** | **Comment** |
|---|---|---|---|---|---|---|---|---|---|---|
| 1 | QJZ-18 | 04:25:19 PM | 90.833 | SR | 0.109 | 589 | 100.00 | 0.120 | 20.2 | |
| 2 | QJZ-18 | 04:25:27 PM | 90.833 | SR | 0.109 | 589 | 100.00 | 0.120 | 20.1 | |
| 3 | QJZ-18 | 04:25:34 PM | 90.833 | SR | 0.109 | 589 | 100.00 | 0.120 | 20.1 | |
| 4 | QJZ-18 | 04:25:42 PM | 90.833 | SR | 0.109 | 589 | 100.00 | 0.120 | 20.1 | |
| 5 | QJZ-18 | 04:25:49 PM | 90.833 | SR | 0.109 | 589 | 100.00 | 0.120 | 20.1 | |
| 6 | QJZ-18 | 04:25:56 PM | 90.833 | SR | 0.109 | 589 | 100.00 | 0.120 | 20.0 | |

______________________________
Signature

Figure S72. OR value of compound **8** in MeOH

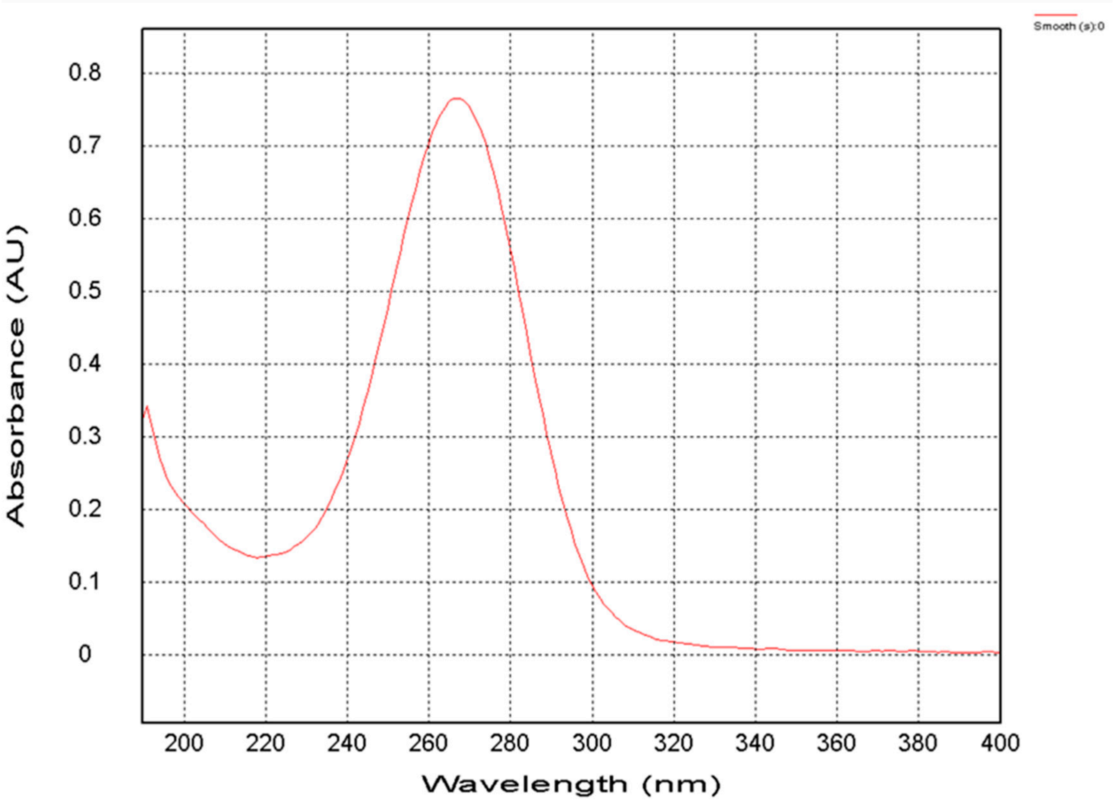


Figure S73. UV spectrum of compound **8** in MeOH

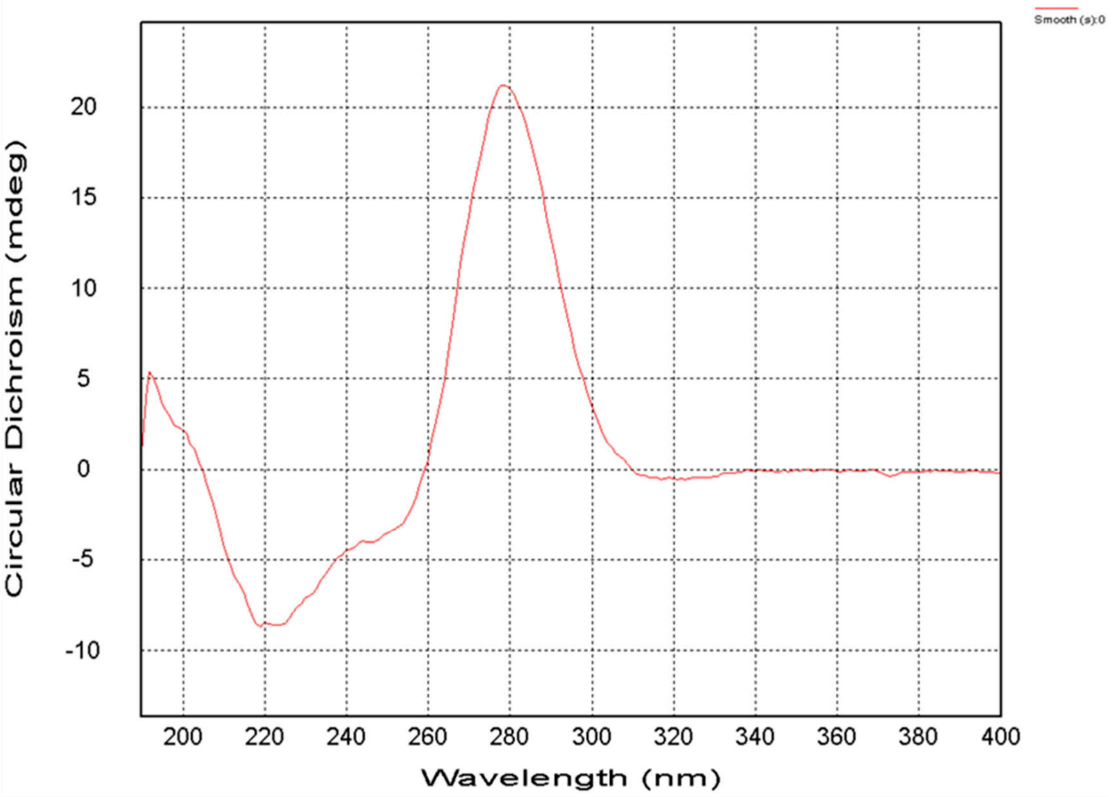


Figure S74. CD spectrum of compound **8** in MeOH

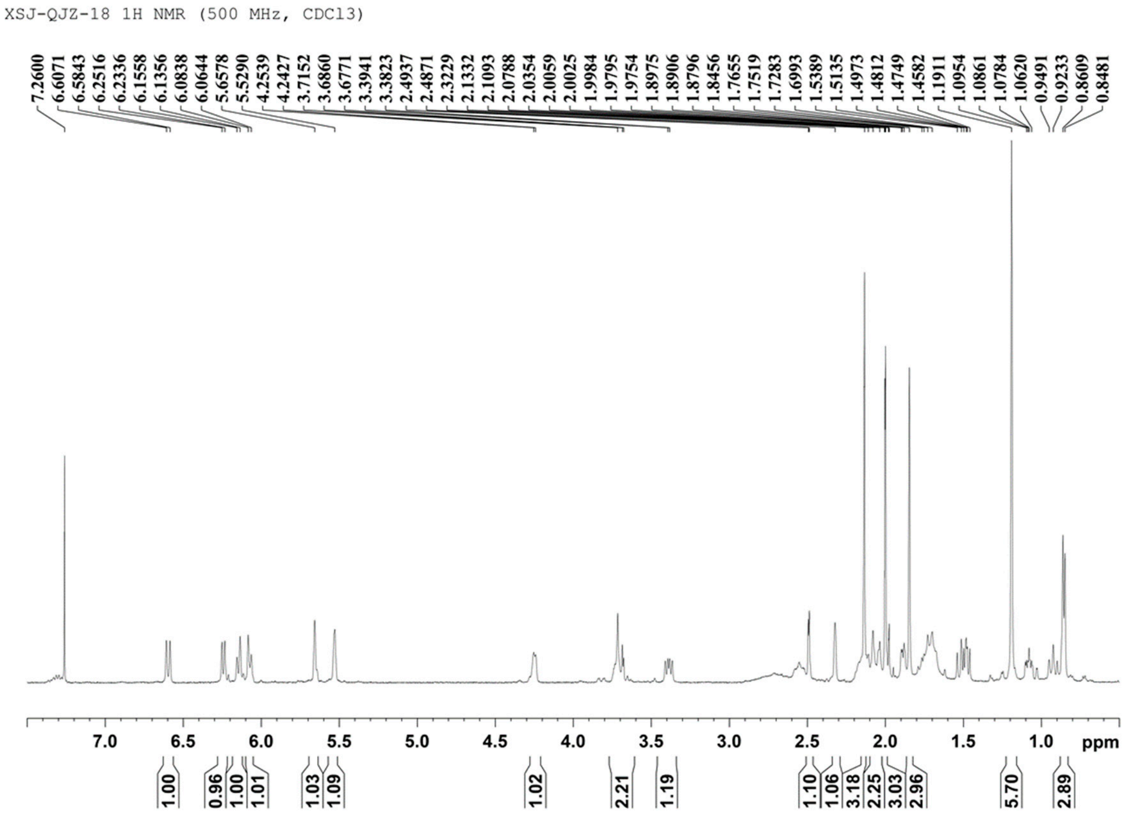


Figure S75. $^{1}$H NMR spectrum of compound **8** in $CDCl_3$

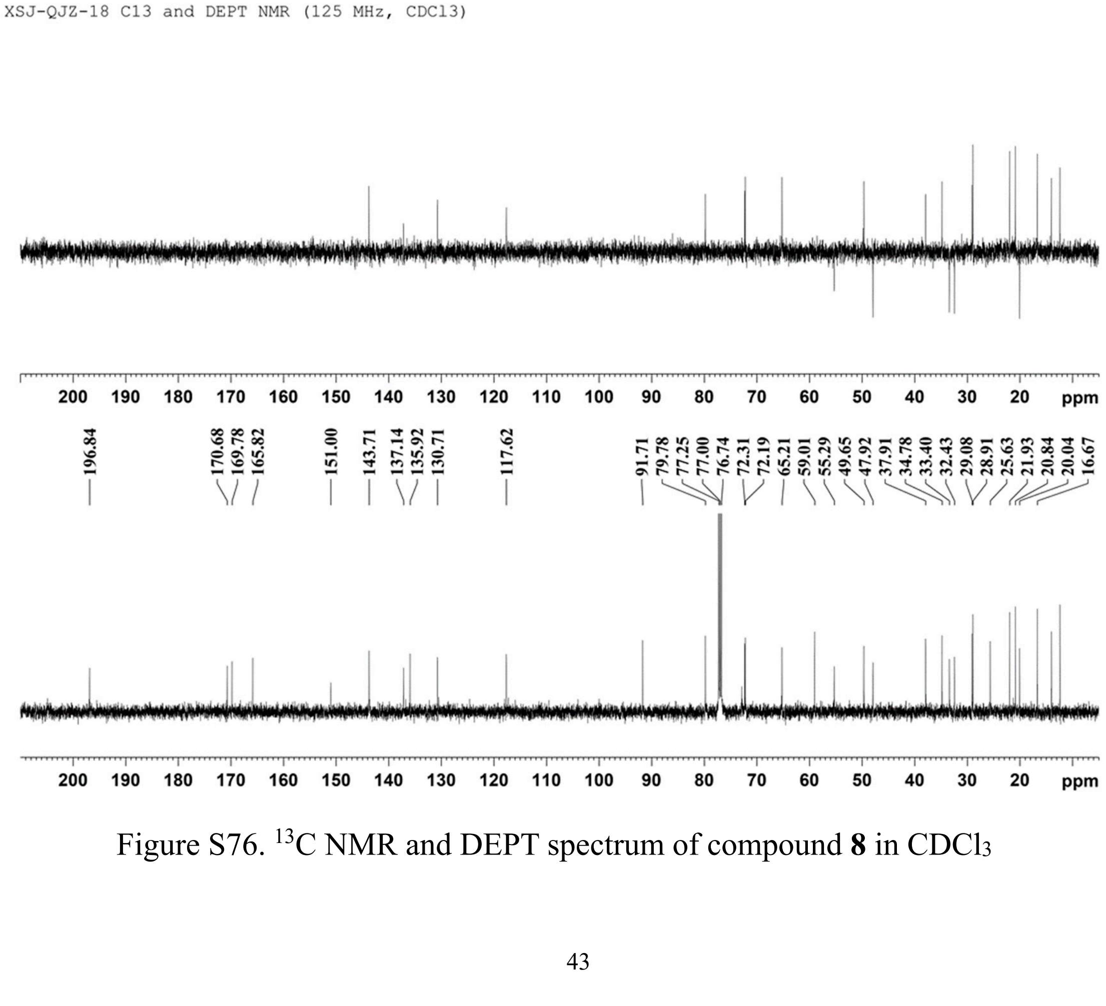


Figure S76. $^{13}$C NMR and DEPT spectrum of compound **8** in $CDCl_3$

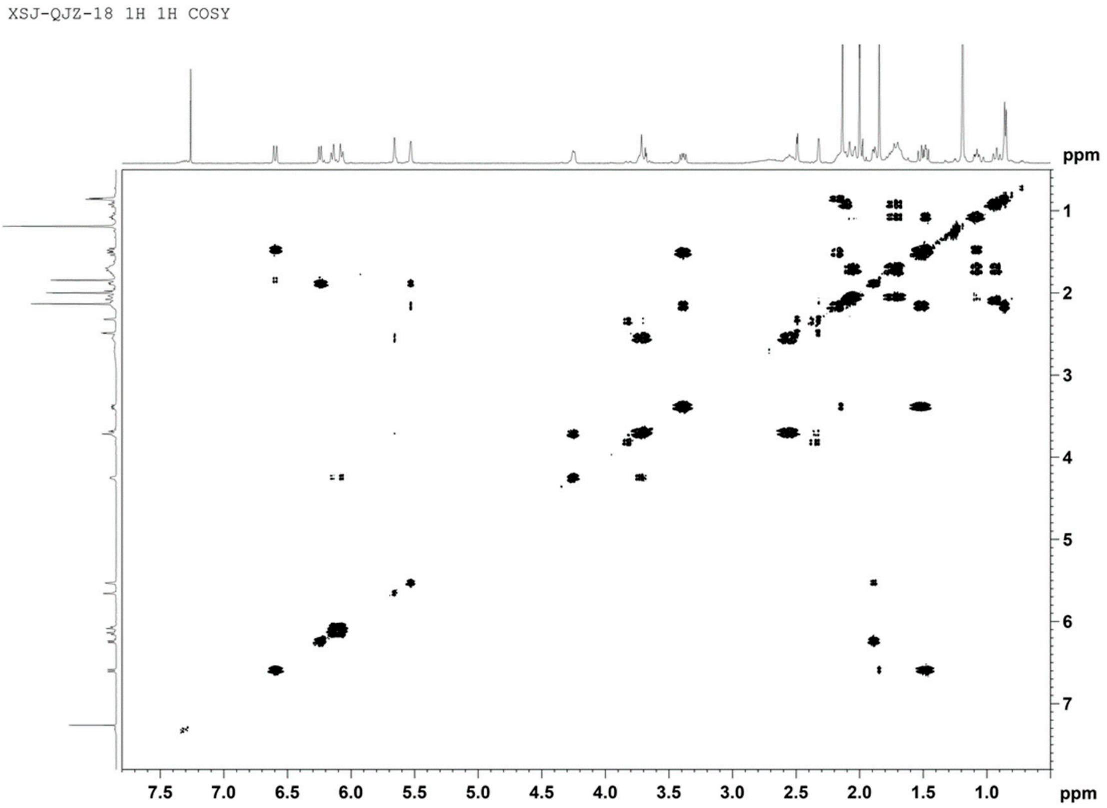


Figure S77. $^{1}H$-$^{1}H$ NMR spectrum of compound **8** in $CDCl_3$

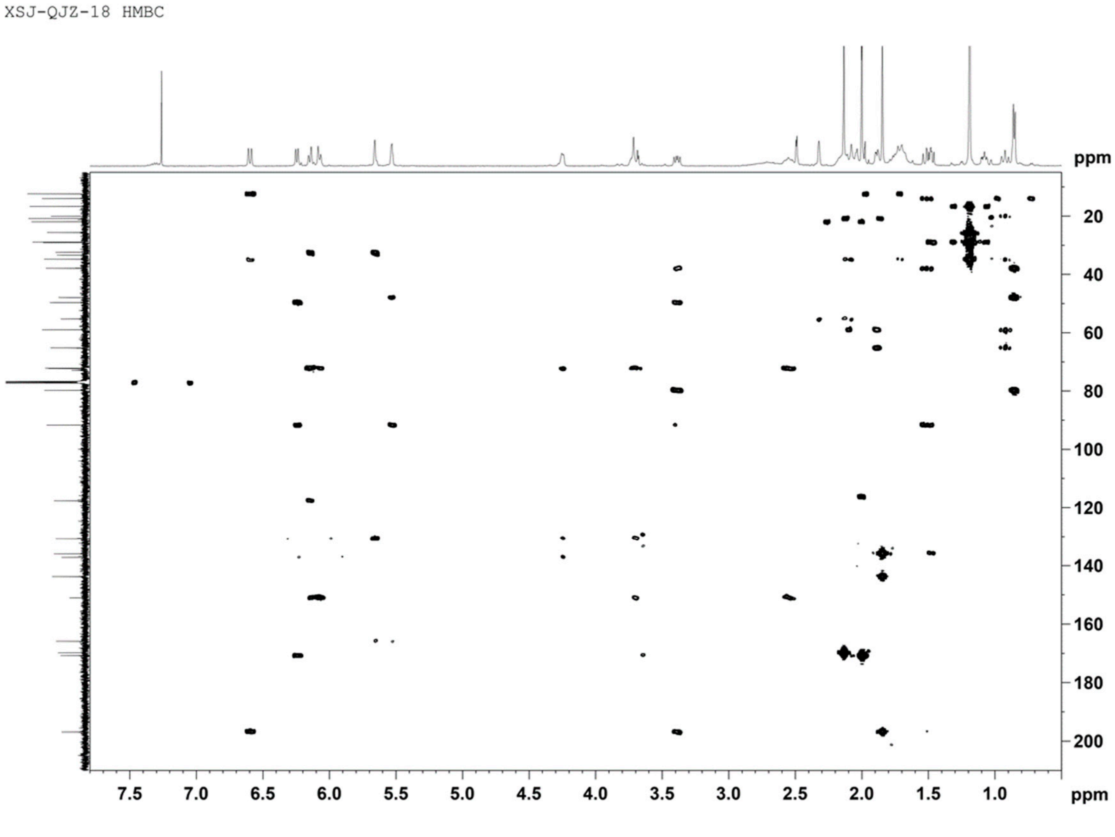


Figure S78. HMBC spectrum of compound **8** in $CDCl_3$

XSJ-QJZ-18 HSQC

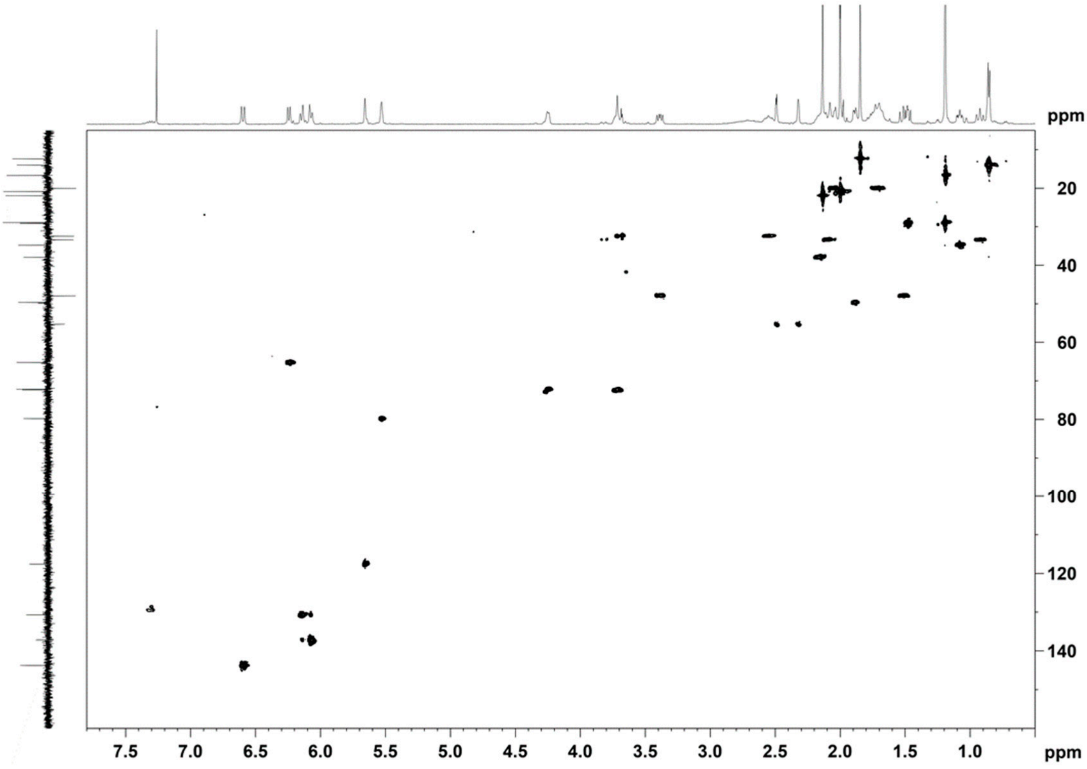


Figure S79. HSQC spectrum of compound **8** in $CDCl_3$

XSJ-QJZ-18 NOESY

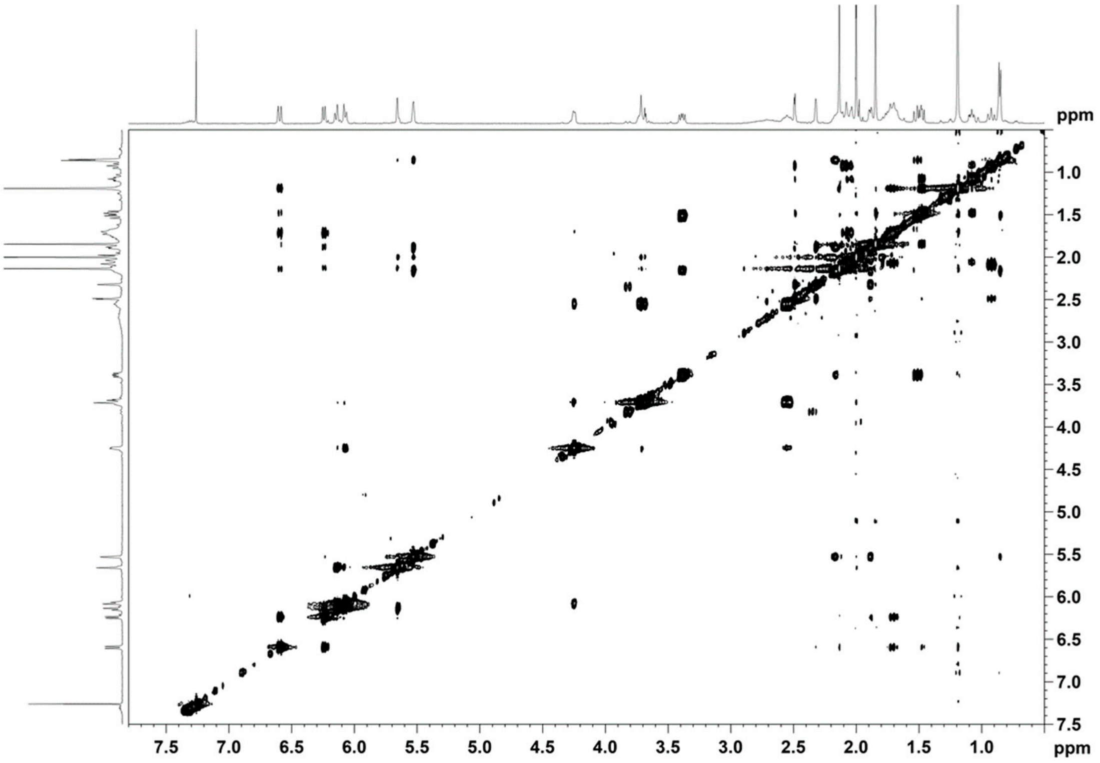


Figure S80. NOESY spectrum of compound **8** in $CDCl_3$

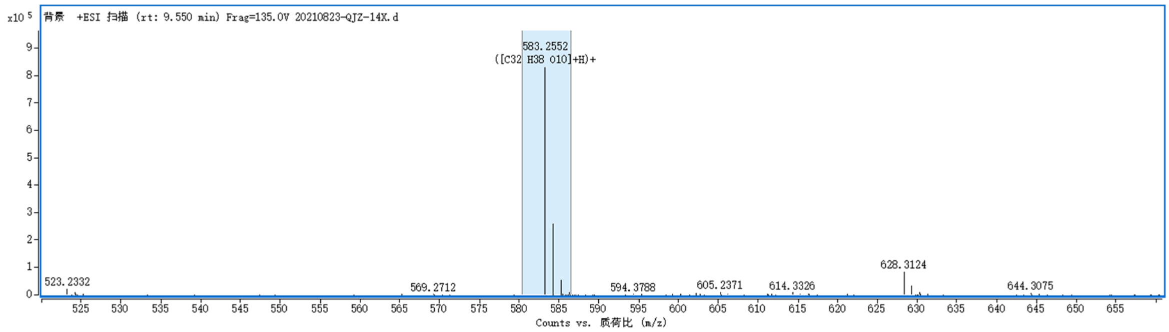


| Best | Formula(M) | Ion Formula | Tgt (m/z) | m/z | Diff (ppm) |
|---|---|---|---|---|---|
| TRUE | $C_{32}H_{38}O_{10}$ | $C_{32}H_{39}O_{10}$ | 583.2538 | 583.2552 | 2.40 |

Figure S81. HRESIMS spectrum of compound **9**

**Rudolph Research Analytical**

Saturday, 10/09/2021

This sample was measured on an Autopol VI, serial number 90079,
manufactured by Rudolph Research Analytical,Hackettstown,NJ.

LotID : QJZ-14
Set Temperature : 20.0
Temp Corr : OFF

| n | Average | Std.Dev. | Maximum | Minimum |
|---|---|---|---|---|
| 6 | 77.778 | 0.3833 | 78.049 | 77.236 |

| S.No | Sample ID | Time | Result | Scale | OR °Arc | WLG | Lg.mm | Conc. | Temp. | Comment |
|---|---|---|---|---|---|---|---|---|---|---|
| 1 | QJZ-14 | 04:47:14 PM | 77.236 | SR | 0.095 | 589 | 100.00 | 0.123 | 20.1 | |
| 2 | QJZ-14 | 04:47:22 PM | 78.049 | SR | 0.096 | 589 | 100.00 | 0.123 | 20.1 | |
| 3 | QJZ-14 | 04:47:29 PM | 77.236 | SR | 0.095 | 589 | 100.00 | 0.123 | 20.1 | |
| 4 | QJZ-14 | 04:47:37 PM | 78.049 | SR | 0.096 | 589 | 100.00 | 0.123 | 20.0 | |
| 5 | QJZ-14 | 04:47:45 PM | 78.049 | SR | 0.096 | 589 | 100.00 | 0.123 | 20.0 | |
| 6 | QJZ-14 | 04:47:52 PM | 78.049 | SR | 0.096 | 589 | 100.00 | 0.123 | 20.0 | |

Signature

Figure S82. OR value of compound **9** in MeOH

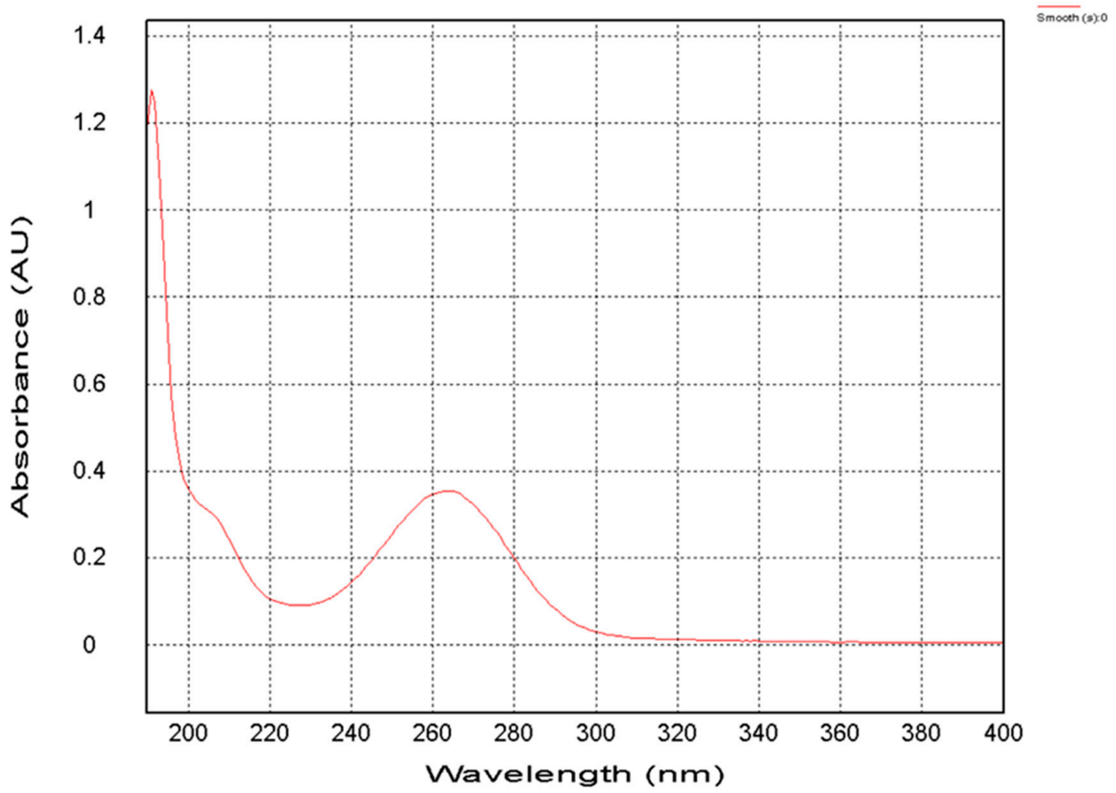


Figure S83. UV spectrum of compound **9** in MeOH

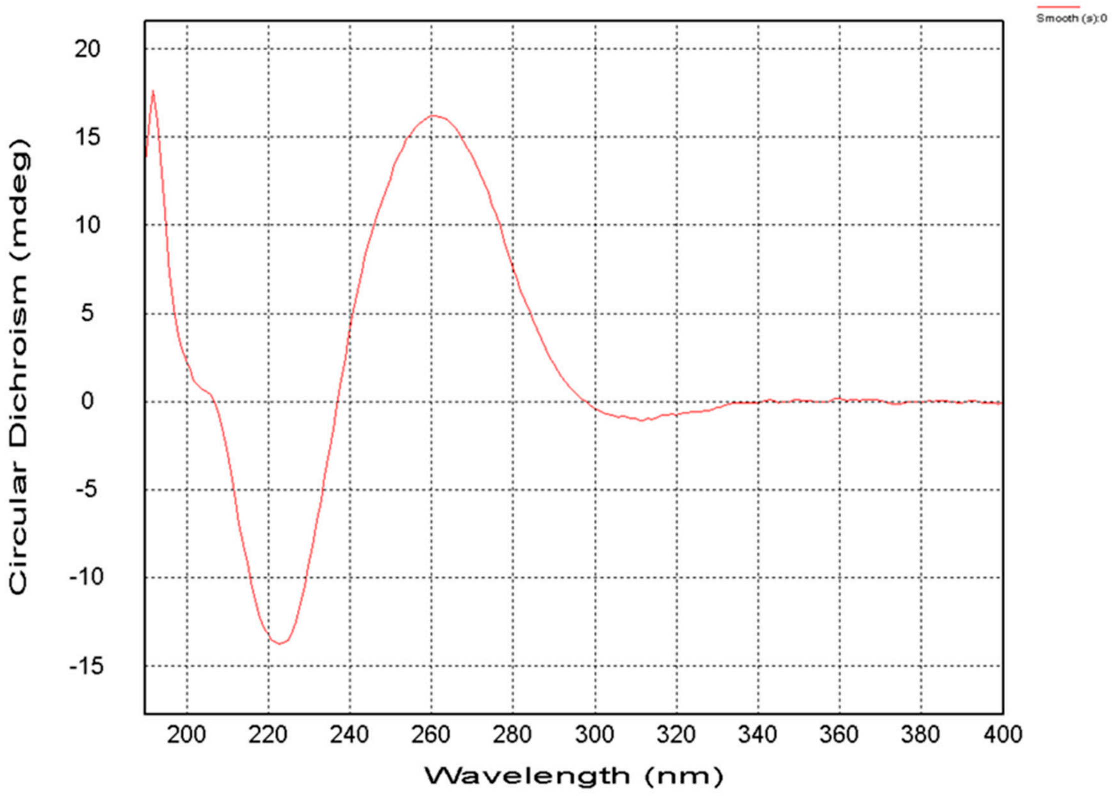


Figure S84. CD spectrum of compound **9** in MeOH

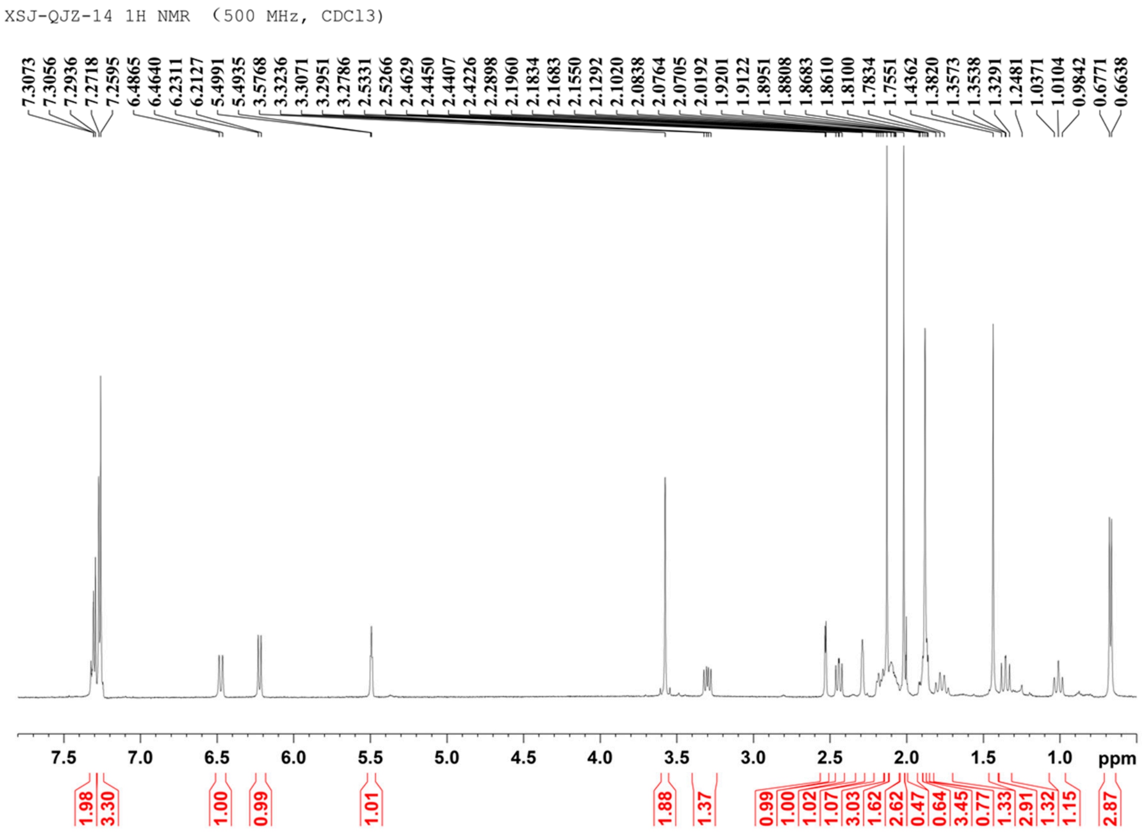


Figure S85. $^{1}$H NMR spectrum of compound **9** in $CDCl_3$

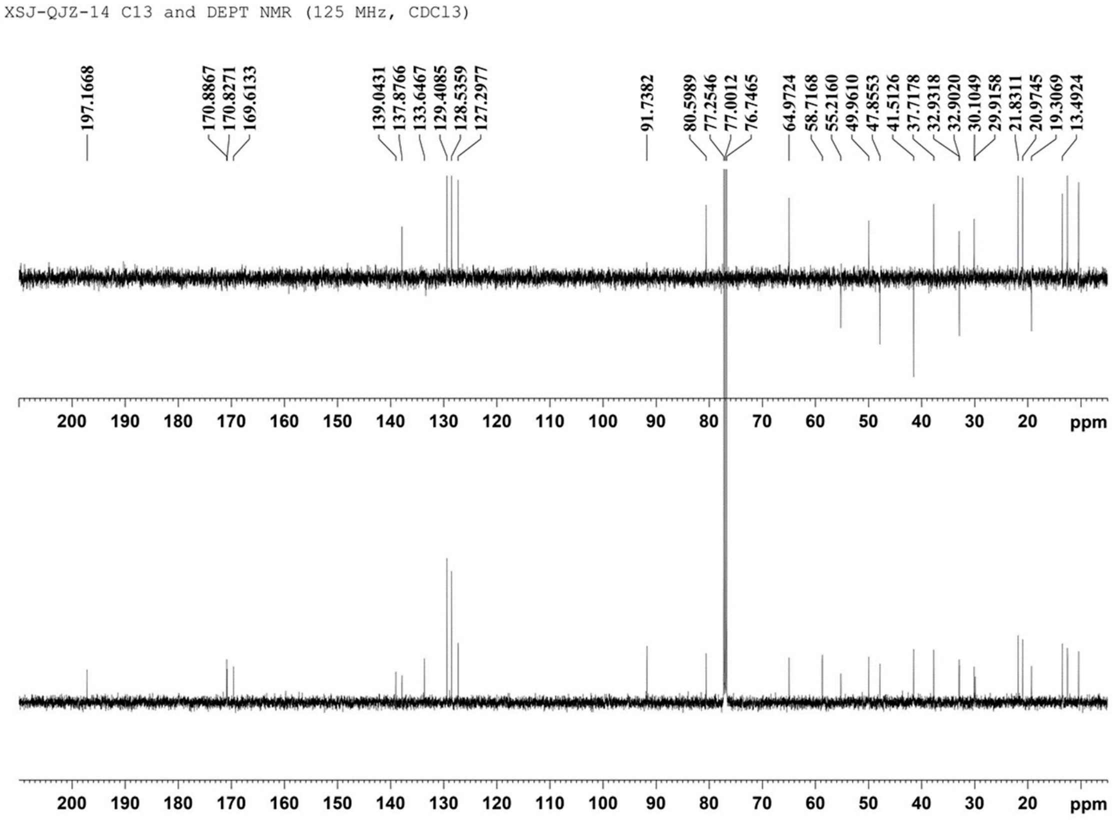


Figure S86. $^{13}$C NMR and DEPT spectrum of compound **9** in $CDCl_3$
